# Maximal lactate accumulation rate (${\dot{\mathrm{c}}}$La_max_): Current evidence and future directions for exercise testing and training

**DOI:** 10.1007/s00421-025-06022-7

**Published:** 2025-10-31

**Authors:** Oliver J. Quittmann

**Affiliations:** 1 Institute of Movement and Neurosciences, German Sport University Cologne, Cologne, Germany; 2 Exercise Science, Coaching and Performance Enhancement (E.S.C.A.P.E.) Network, Cologne, Germany

**Keywords:** Glycolysis, Anaerobic, Exercise physiology, Performance, Lactate production, VLa_max_

## Abstract

Maximal lactate accumulation rate (${\dot{\mathrm{c}}}$La_max_) has recently gained increased attention in exercise science as a parameter characterising the maximum power of glycolytic metabolism. Since a lot of sports put high demands on both oxidative as well as substrate-level phosphorylation, ${\dot{\mathrm{c}}}$La_max_ may be a promising augmentation to metabolic profiling in athletes. However, scientific examinations still demonstrate inconsistencies in terms of terminology, procedures, calculations and application that highlight the need for an extensive literature review. This review aims to summarise the current evidence of ${\dot{\mathrm{c}}}$La_max_ and provide recommendations for future research and application in practice. Findings of N = 60 accepted peer-review Journal articles in English language were extracted to highlight the origin, development, terminology, procedures, reliability, specificity, applicability and adaptability of ${\dot{\mathrm{c}}}$La_max_. It provides a critical view of this field of research to assist international colleagues who might not yet be familiar with ${\dot{\mathrm{c}}}$La_max_. It is evident that ${\dot{\mathrm{c}}}$La_max_ has spread across the globe and is already applied in various sports like (hand-)cycling, running, swimming, rowing, kayaking and paratriathlon. Sport-specific all-out sprint tests lasting 10–12 s and measurements of post-exercise lactate concentration every minute for 10 min are recommended to determine ${\dot{\mathrm{c}}}$La_max_ that demonstrates a high reliability and specificity. Whereas ${\dot{\mathrm{c}}}$La_max_ is associated with sprint performance and strength parameters, its utility to predict/simulate individual performances ≥ 1 min is still inconclusive. Future studies need to validate ${\dot{\mathrm{c}}}$La_max_ by means of enzyme activity and/or muscle fiber typology, focus on female athletes (currently 25%) and assess its adaptability to certain training regimes.

**Supplementary Information:**

The online version contains supplementary material available at 10.1007/s00421-025-06022-7.

## Introduction

Energy metabolism and its persistent resynthesis of adenosine triphosphate (ATP) is essential for skeletal muscle contractile activity. To meet the manifold ATP demands of exercise, that may increase to > 150-fold from rest (Nyberg & Jones 2022), several metabolic pathways are available that can be classified as *oxidative (‘aerobic’) and substrate-level (‘anaerobic’) phosphorylation*. Whereas oxidative phosphorylation is the major contributor for intense exercise beyond ∼ 1 min (Gastin 2001), ‘anaerobic’ pathways are particularly important for very intense efforts (lasting seconds) as well as intermittent activities (Hargreaves & Spriet 2020). In this context, lactate metabolism seems to be a link between glycolytic and oxidative metabolism and *“has risen to major importance in twenty-first century biology”* (Brooks et al. 2021). Glycolysis represents the breakdown of carbohydrates (glycogen or glucose) to ATP by restoring important coenzymes (e. g. the oxidised form of nicotinamide adenine dinucleotide, NAD^+^) and forming lactate (Barclay 2017). At the end of glycolysis, pyruvate and lactate are formed that can still be used for energy supply. As such, lactate *“enables the uncoupling of carbohydrate-driven mitochondrial energy generation from glycolysis”* (Rabinowitz and Enerbäck 2020). Outdated assumptions that lactate causes fatigue, burn or cramps are replaced by the understanding that this molecule is part of *metabolic flexibility, cell signalling and adaptation* (Brooks et al. 2021). Readers who are interested in *the historical context of lactate* from its discovery in 1780 to its paradigm shift in the 1980s and beyond, are forwarded to another well-crafted review (Ferguson et al. 2018). Given that many sports demonstrate a high reliance on glycolytic energy supply, it seems reasonable to quantify this metabolism in exercise testing.

Exercise testing allows for new mechanistic *insights into human physiology* and helps to individualise/optimise training prescription. As such, expanding the toolset of available parameters is beneficial for fundamental research as well as practical application. Parameters targeting ‘anaerobic’ abilities have already been applied and include (but are not limited to) the curvature constant of the hyperbolic power- or velocity–time relationship (Vanhatalo et al. 2016), work done above all-out end-test power (Vanhatalo et al. 2007), maximal accumulated oxygen deficit (Noordhof et al. 2010) and anaerobic speed reserve (Thron et al. 2024a). Even though these parameters have shown to be helpful in characterising athletes, these parameters seem to be surrogates of maximal ‘anaerobic’ *capacity* and do not represent the maximal *rate* of substrate-level phosphorylation—known as ‘anaerobic’ or glycolytic *power* (Vandewalle et al. 1987; Heck et al. 2003). Energy system contribution differs substantially *between* running events as well as *during* the time course of a give event (Gastin 2001). Even more striking, Gastin (2001) demonstrated that energy contribution profiles differ between endurance- and sprint-trained individuals. Hence, *augmenting the toolset of exercise testing* by means of glycolytic *power* seems promising to describe another layer of ‘anaerobic’ abilities which is of particular interest in sports demonstrating high glycolytic energy contribution. Whereas the maximal rate of oxidative phosphorylation (maximum oxygen uptake, ${\dot{\mathrm{V}}\mathrm{O}}$_2max_) has been assessed for > 100 years (Hill & Lupton 1923), the *maximal rate of glycolysis* (maximal lactate accumulation rate) has been quantified for only > 20 years.

Several review articles (especially in recent years) have already been published that covered the theoretical framework of simulating energy metabolism (Mader 2003; Wackerhage et al. 2022; Dunst et al. 2025), methodological aspects of ‘anaerobic’/glycolytic exercise testing (Heck et al. 2003; Wackerhage et al. 2025; Langley et al. 2025), reliability of maximal glycolytic power (Fernandez-Jarillo & Lomero-Arenas 2025; Langley et al. 2025) as well as performance in swimming and rowing (Olbrecht 2011; Treff et al. 2021). Since four of these nine reviews have been published in 2025, it demonstrates the urgent need for an overview of this topic that facilitates the participation of researchers unfamiliar in this area. While these review articles offer condensed information in several important aspects of this field of research, a holistic view of all of these aspects is still missing. Furthermore, a critical appraisal of these reviews offers a more objective view on maximal lactate accumulation rate in science and practice. A similar contrast between the wide-spread application and (rather) consistent procedures of ${\dot{\mathrm{V}}\mathrm{O}}$_2max_ testing and rather inconsistent procedures used to test the maximal rate of lactate accumulation has been mentioned recently (Wackerhage et al. 2025). The authors systematically searched for existing literature, highlight several problems with testing glycolytic power and provide concrete recommendations for future research and practical application. While the present author appreciates the colleagues’ intention to develop this field of research and the provided ideas and discussions, most of their arguments are grounded on a simulation model of energy metabolism that has not yet been validated by experimental studies (Mader 2003). This lack of research is addressed in their recommendations for future investigations while the few already existing studies indicating a substantial difference between simulated and experimental parameters are not referred to (Hauser et al. 2014; Wahl et al. 2017; Ji et al. 2021; Sablain et al. 2025).

This invited review aims to summarise the current evidence of *maximal lactate accumulation rate* to provide recommendations for exercise testing and training and guidelines for future research. It is dedicated to academic colleagues as well as practitioners who think about entering this topic and seek for a condensed yet scientific sound overview. This work is grounded on *N* = *60 peer-reviewed Journal articles* (excluding conference abstracts) that have been accepted in English language until October 2025 (n = 11 have already been accepted in 2025). These include 31 original articles, 12 reliability analyses, 9 reviews, 4 interventions as well as 1 supplement, 1 single-case study, 1 pilot study and 1 letter to the editor. These articles described and/or analysed maximal lactate accumulation rate in various sports with respect to different aspects. The structure of this review is designed to allow an immediate access to areas of interest as these are highlighted by the section headings. The beginning/end of every paragraph/chapter provides a summary/conclusion of the aforementioned aspects and puts these in perspective. The following 8 chapters of this review highlight the *origin, development and terminology*, *procedures and calculations*, *reliability*, *specificity*, *relationships to performance and physiology*, *adaptability* and *future directions*. Lastly, *recommendations* for investigating/applying maximal lactate accumulation rate in science/practice are provided.

## Origin, development and terminology

In 1986, Alois Mader and Hermann Heck aimed to develop a theoretical concept to explain the *metabolic origin of ‘anaerobic threshold’* (Mader & Heck 1986), which build up on Mader’s habilitation from 1984 (Wackerhage et al. 2022). Within their model, they assumed an interaction between oxidative and substrate-level phosphorylation, which are represented by their respective maximal energy rates. Whereas ${\dot{\mathrm{V}}\mathrm{O}}$_2max_ represented the power of oxidative energy supply, maximal glycolytic energy supply was expressed as *maximal lactate formation rate.* They assumed that the maximal lactate formation rate (a surrogate of maximal rate of glycolysis) ranges from 0.1 to 1.3 mmol/l/s (Mader & Heck 1986). Furthermore, it was stated that net lactate formation represents the difference between ‘gross’ lactate formation rate and the rate of lactate disappearance, with the latter being proportional to oxygen uptake. Based on a series of equations, they were able to estimate a *‘crossing point’*, at which net lactate formation equals zero—also known as maximal lactate steady-state (MLSS). Assuming a constant ${\dot{\mathrm{V}}\mathrm{O}}$_2max_, they simulated that higher maximal rates of glycolysis result in lower outcomes of MLSS and fat oxidation rate (Fat_max_, MFO). Accordingly, given a fixed maximum rate of glycolysis, MLSS and fat oxidation are assumed to increase for improvements in ${\dot{\mathrm{V}}\mathrm{O}}$_2max_.

In a simplified manner, ${\dot{\mathrm{V}}\mathrm{O}}$_2max_ and the maximal rate of glycolysis are assumed to (potentially) predict MLSS and/or fat oxidation rate (Appendix 1). Mader published an advanced model of cytosolic phosphorylation in 2003 that described the pH-dependent dynamics of energy metabolism by no less than *33 equations*. This may indicate why his work refused to get initial international resonance. He assumed that maximal glycolytic rate *“is a function of the concentration of glycolytic enzymes”* (Mader 2003). Whereas adenosine di- and monophosphate are considered to be activators of glycolysis, a low pH (high concentration of H^+^ ions) is considered to reduce glycolysis by an inhibition of phosphofructokinase (PFK) (Wackerhage et al. 2022). Even though Mader’s simulation model(s) may help to understand some principles of physiological processes, this model has not yet been validated by experimental research that applied a sound design to *independently, transparently* and *quantitatively* test its practical value in terms of predicting an individual’s MLSS, Fat_max_ and/or MFO. Since discussing the various simulation approaches of energy metabolism is beyond the scope of this review, readers who are interested in these theoretical concepts are forwarded to specific review articles (Dunst et al. 2025; Wackerhage et al. 2022). *In summary,* maximal rate of glycolysis started as a theoretical parameter to describe the origin of MLSS as used in energy simulation approaches.

The first experimental approaches to determine the maximal glycolytic flux by means of post-exercise lactate accumulation were performed by the research group of the University of Milan (Italy) that was highly influenced by the work of Rodolfo Margaria in the 1960s (Margaria et al. 1964). Readers interested in the physiological findings of the Milan research group on the energetics of muscular exercise are forwarded to the well-crafted summaries of Guido Ferretti (Ferretti 2015, 2023). They observed a linear relationship between high-intensity exercise duration and maximal post-exercise lactate concentration when intensity was kept fixed. The slope of the resulting regression lines was considered to characterise the individuals’ maximal rate of lactate accumulation (Grassi et al. 1995). This is in contrast to the terminology used by Mader and Hack who referred to a theoretical/cellular rate of lactate formation/production (Mader & Heck 1986; Heck et al. 2003). However, there are similarities between schools as both refer to *“maximal lactic power”* (Heck et al. 2003; Ferretti 2023). In contrast to the *Milano-approach*, Alois Mader argues that a single sprint of 15-s and the corresponding post-exercise lactate kinetics should be appropriate to determine maximal lactate accumulation rate (Wackerhage et al. 2022), that was done in the early 2000s (Poffé et al. 2024).

**Fig. 1:**
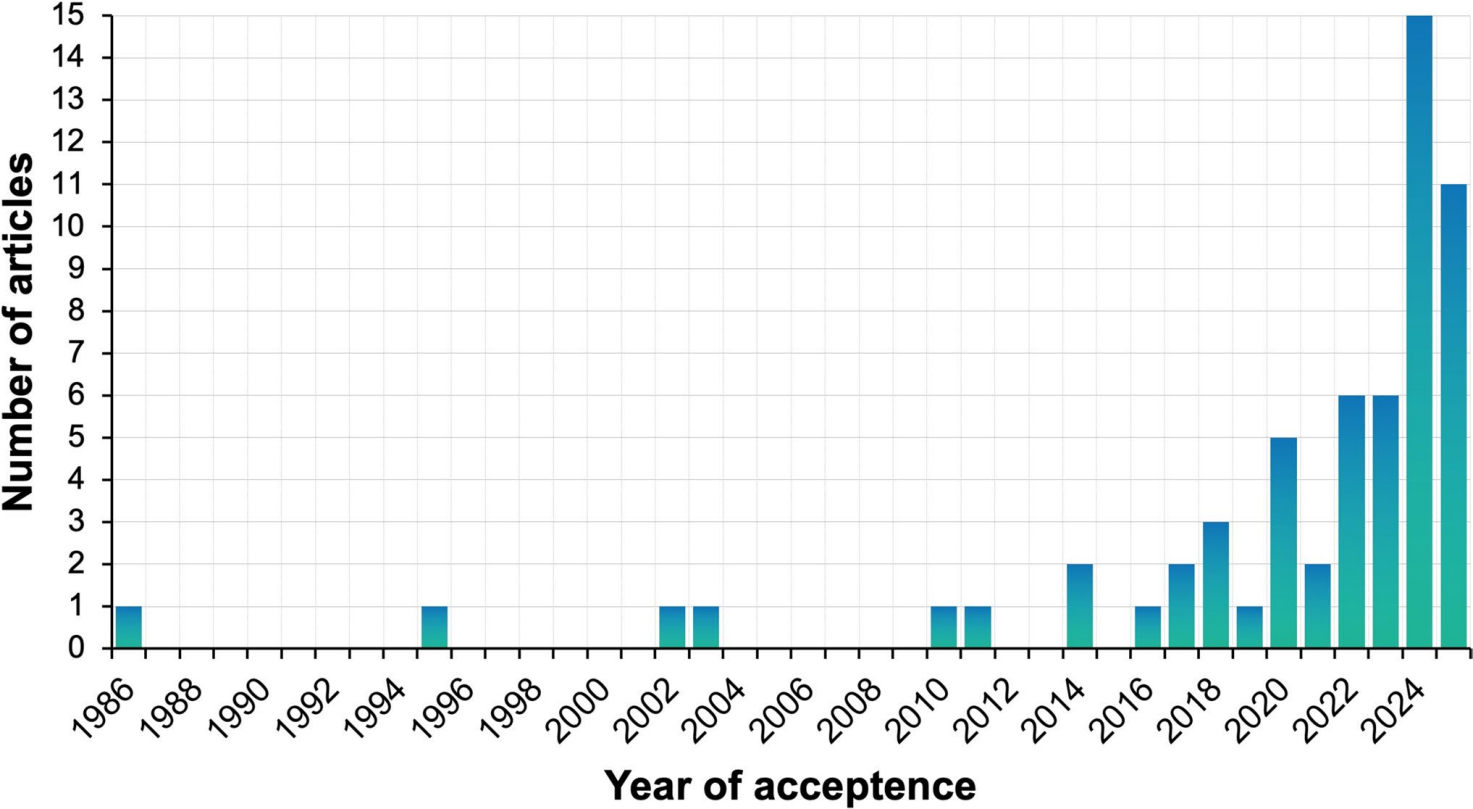
Number of accepted peer-reviewed Journal articles per year (until October 2025)

Several articles mentioned that maximal lactate accumulation rate has gained *increased scientific attention* in the international literature (Treff et al. 2021; Held et al. 2023; Langley et al. 2024, 2025; Pohl et al. 2024; Meixner et al. 2025b; Wackerhage et al. 2025; Fernandez-Jarillo & Lomero-Arenas 2025; Micke et al. 2025). However, this admittedly plausible assumption has not yet been verified by *empirical* evidence. Hence, a detailed analysis of the existing literature in terms of articles per year as well as the origin of affiliations by country was performed. In the last 4 years, accepted journal articles on maximal lactate accumulation rate have increased substantially (Fig. 1). From the early 2000s until 2011, articles remained mostly theoretical with the exception of a crossover intervention in swimming (Sperlich et al. 2010). The year 2014 demarcates the beginning of an increased scientific examination of this parameter that demonstrated a rapid incline in 2024 (n = 15 articles). To highlight the distribution of research across the globe, all authorships were checked for their affiliation and counted per country. This augments a previous review that only analysed first-authorships, and found that German researcher covered 22/27 articles (> 81%) (Langley et al. 2025), which seems to display only a fragment of the bigger picture.

From a total of N = 260 authorships, n = 178 (*68.5%*) were traced back to Germany, which highlights the original origin and consequently limited expansion of this parameter. The remaining n = 82 authorships originated from *Belgium* (n = 11, 4.2%), *South Korea* (n = 11, 4.2%), *Japan* (n = 9, 3.5%), *United States of America* (n = 8, 3.1%), *United Kingdom* (n = 7, 2.7%), Italy (n = 6, 2.3%), *Greece* (n = 5, 1.9%), *Poland* (n = 5, 1.9%), *Brazil* (n = 4, 1.5%) *Norway* (n = 3, 1.2%), *Switzerland* (n = 3, 1.2%), *Estonia* (n = 3, 1.2%), *New Zealand* (n = 2, 0.8%), *Sweden* (n = 2, 0.8%), *Spain* (n = 2, 0.8%) and *Austria* (n = 1, < 0.4%). The distribution of affiliations is even more striking when analysing the first and second half of (each n = 30) articles separately. In the first subsample (until March 2023), the proportion of German affiliations was more than *80%* (110/136), whereas a German contribution of less than *55%* (68/124) was observed in the second half (until October 2025). It is a good development that maximal lactate accumulation rate has recently *started to spread across the globe*. Accordingly, this review aims to encourage scientists from various countries/laboratories to participate in this field of research and share their expertise/perspectives.

In contrast to ${\dot{\mathrm{V}}\mathrm{O}}$_2max_, that demonstrates a rather high consistency in terms of terminology (maximal oxygen uptake/consumption) and abbreviations (${\dot{\mathrm{V}}\mathrm{O}}$_2max_/${\dot{\mathrm{V}}\mathrm{O}}$_2_max) (Nolte et al. 2023), there is a high inconsistency regarding maximal lactate accumulation rate (Appendix 2) (Langley et al. 2025). A total of *six categories* were identified for the terminology of this parameter. Firstly, a total of n = 16 articles used something similar to *maximal rate of glycolysis* (Mader & Heck 1986; Mader 2003; Heck et al. 2003; Hauser et al. 2014; Adam et al. 2015; Nitzsche et al. 2020; Wawer et al. 2020; Wackerhage et al. 2022; Quittmann et al. 2022a; Yang et al. 2023; Harnish et al. 2023; Pohl et al. 2024; Clark & Macdermid 2025; Meixner et al. 2025a, b; Sablain et al. 2025). Secondly, (in particular earlier) articles (n = 13) described it as *maximal lactate production rate* (Mader & Heck 1986; Heck et al. 2003; Hauser et al. 2014; Adam et al. 2015; Wahl et al. 2017; Nitzsche et al. 2018b; Hommel et al. 2019; Ji et al. 2021; Wackerhage et al. 2022; Haase et al. 2024; Langley et al. 2024; Poffé et al. 2024; Teixeira et al. 2022). It appeared obvious that these two terminologies (especially the latter) were frequently used in articles that focused on simulating energy metabolism. Hence, it is recommended to differentiate terminology by means of a *theoretical* parameter (used in simulations and refers to the muscle level) and an *empirical* parameter that is measured in experiments on the whole-body level.

The most frequently used terminology (n = 37) referred to this parameter as (something like) *maximal lactate accumulation rate* (Sperlich et al. 2010; Manunzio et al. 2016; Quittmann et al. 2018, 2020, 2021a, b, 2022a, b; Nitzsche et al. 2020; Zwingmann et al. 2020; Treff et al. 2021; Dunst et al. 2023a, b; Schünemann et al. 2023; Yang et al. 2023, 2024; Mavroudi et al. 2023; Held et al. 2023; Harnish et al. 2023, 2024; Thron et al. 2024b; Langley et al. 2024, 2025; Meixner et al. 2024a, b, 2025a; Reinpõld et al. 2024; Sengoku et al. 2024; Porter and Langley 2025; Keller & Wahl 2025; Sablain et al. 2025; Grassi et al. 1995; Fischer et al. 2025; Haase et al. 2025; Fernandez-Jarillo & Lomero-Arenas 2025; Wackerhage et al. 2025; Micke et al. 2025). This term takes into account that—during whole-body activities and capillary blood measurements—there is a systemic turnover of lactate that is simultaneously released and taken up by various tissues, with skeletal muscles being the (by far) most influential (van Hall 2010; Ferretti 2023). Hence, referring to an experimental parameter as lactate *production* rate seems to be misleading. In contrast, *accumulation* rate implies that there is a change in lactate *concentration*. However, in few articles (n = 3), the authors seemed to stress this aspect by using another (fourth) category as *rate of blood lactate concentration* (Nitzsche et al. 2018a; Meixner et al. 2024a, b). However, *maximal lactate accumulation rate* is far more common and implies the same.

The fifth (n = 13) and sixth (n = 7) of terminology categories described this parameter as *glycolytic/lactic power* (Heck et al. 2003; Quittmann et al. 2018, 2020; Treff et al. 2021; Schünemann et al. 2023; Held et al. 2023; Haase et al. 2024; Thron et al. 2024b; Langley et al. 2024, 2025; Reinpõld et al. 2024; Sengoku et al. 2024; Keller & Wahl 2025) and *anaerobic/glycolytic capacity* (Olbrecht 2011; Zwingmann et al. 2020; Wawer et al. 2020; Ji et al. 2021; Harnish et al. 2023; Wagner et al. 2024; Harnish & Miller 2023), respectively. Whereas *capacity* referrers to *“the sum of all work that can be gained from energy stored in chemical form”*, *power* is characterised by *“the maximal metabolic rates of the different energy transfer systems”* (Heck et al. 2003). As such, practitioners can refer to this parameter as anaerobic, glycolytic or (at best) *lactic power*, but should *avoid* mentioning the term *capacity* in this context. However, future studies may examine the interplay between ‘anaerobic’ power and capacity in deliberately trained athletes.

*In summary,* a differentiation in terminology between a *theoretical* parameter used in simulations (*maximal rate of glycolysis*) and an *empirical* parameter used in experiments (*maximal lactate accumulation rate*) is recommended, while the latter may also be called *glycolytic/lactic power*. Analogous to the distinction between *maximal* and *peak* oxygen uptake (as a measure of oxidative power) (Poole and Jones 2017), there is reason to discuss the application in the field of lactate accumulation rate as well. We could agree to use the term *maximal* lactate accumulation rate if we assume that the procedures used are suitable to detect a maximum rate (as far as possible). If there is a reasonable that the procedure does *not* allow to detect a *maximal* rate, but and we still want to provide the recorded outcomes, we could use the term *peak* lactate accumulation rate to imply that we believe that other conditions (e. g. a shorter test time and/or a higher blood sampling rate) might have led to higher (more accurate) results (see Chapter 3). Whereas terminology appears to be quite consensual, the use abbreviations is still a (highly) debatable topic.

A total of *23 different abbreviations* were found within the N = 60 articles (Appendix 2). The most frequently used abbreviations were $\dot{V}$*La*_*max*_ (n = 12), *VLa*_*max*_ (n = 12), *νLa*_*max*_ (n = 8), $\dot{c}$*La*_*max*_ (n = 5), $\dot{V}$*Lamax* (n = 4), and VLamax (n = 3), whereas VLamax, ${\dot{\mathrm{v}}}$La_max_, ^ν^_La.max_ and dLa/dt_max_ were used twice. The remaining n = 13 abbreviations were only used once. Given the high heterogeneity, there is an urgent need for standardised reporting in this field of research. Even though the 13th letter of the Greek alphabet (nu, ν) is used is various articles, this review does *not* recommend its use, since it is a) referred to as specific volume and b) easily mistaken for the small (or even capital) letter ‘v’. This already happened in past as Mader himself introduced VLa_max_ in his review (Mader 2003). Since ‘v’ refers to a (mechanical) velocity, the use of this letter does *not* seem to be applicable in this context. In contrast, Wackerhage et al. (2025) argue that ‘*v*_*max*_’ (in italics) represents the *“maximum velocity”* of oxidative and substrate level phosphorylation (Wackerhage et al. 2025). However, maximal oxygen uptake is usually not abbreviated ‘*v*O_2_max’, which makes it difficult to argue for a similar abbreviation of glycolytic metabolism. Probably due to the analogy to its oxidative counterpart (${\dot{\mathrm{V}}\mathrm{O}}$_2max_), ${\dot{\mathrm{V}}}$La_max_ has been used in the international literature since 2014 (Hauser et al. 2014). Despite its frequent appearance in international literature (even by the author of this invited review), the use of this abbreviation is misleading since maximal lactate accumulation rate does *not* refer to a change in *volume* over time (${\dot{\mathrm{V}}}$), but a change in *concentration* over time.

Hence, our research group had to find another, more appropriate way to abbreviate maximal lactate accumulation rate. Since the small letter ‘c’ is recommended as the standard abbreviation for *concentration* in specific author guidelines (Molecular and Cellular Biology 2023), and the *change* in concentration *over time* is intended, the abbreviation $\dot{c}$*La*_*max*_ has been used in several articles (Quittmann et al. 2022a; Thron et al. 2024b; Sengoku et al. 2024; Keller & Wahl 2025; Fischer et al. 2025). The present author believes that this abbreviation provides an adequate indication of *experimentally-derived* maximal lactate accumulation rate while *avoiding* misconceptions when using ‘ν’, ‘v’ or even ‘${\dot{\mathrm{V}}}$’. However, the author is fully aware that ${\dot{\mathrm{c}}}$La_max_ was introduced by his own group and he declares no interest in dictating the scientific nomenclature. This is just a well-meant recommendation that is still open for discussion. While $\dot{c}$*La*_*max*_ is recommended for describing *maximal lactate accumulation rate* (empirical settings), the use of *dLa/dt*_*max*_ is recommended in simulation-based settings for indicating the (theoretical) *maximal rate of glycolysis* (lactate production). As of now, these abbreviations will be used accordingly in the remaining manuscript while focussing on (empirical) ${\dot{\mathrm{c}}}$La_max._

## Procedures and calculations

Aside from rather common oxidative exercise testing of ${\dot{\mathrm{V}}\mathrm{O}}$_2max_ and running economy for example, glycolytic/’anaerobic’ exercise tests and parameters are controversially discussed. As mentioned by Paul B. Gastin,”*[t]he assessment of anaerobic energy release during exercise is much less precise than the assessment of aerobic energy release […]”* (Gastin 2001). This is in contrast to Mader, who stated that *“details of the parameters […] can be measured in various experimental situations”*, Heck and colleagues mentioned that *“lactic power cannot be measured directly with simple methods*” (Mader 2003; Heck et al. 2003). Challenges in measuring ${\dot{\mathrm{c}}}$La_max_ have already been highlighted in previous articles (Treff et al. 2021; Wackerhage et al. 2022; Meixner et al. 2025b; Wackerhage et al. 2025). As mentioned earlier, measuring (whole-body) lactate concentration does not allow to certainly quantify ‘pure’ lactate production, since *lactate formation, shuttling and uptake* are taking place simultaneously (van Hall 2010; Brooks et al. 2021). Another challenge is to find a type of sprint exercise that is long enough (several seconds) to attain a *high reliance on glycolysis* while being short enough in order to *minimise oxidative energy contribution*. While citing a German reference of Alois Mader, Heck et al. (2003) recommended a 10-s all-out sprint test and recording post-exercise lactate concentration to calculate ${\dot{\mathrm{c}}}$La_max_ according to Eq. 1:

1 $${\dot{\mathrm{c}}\mathrm{La}}_{{{\mathrm{max}}}} { = }\frac{{{\mathrm{La}}_{{{\mathrm{max}}}} - {\mathrm{La}}_{{{\mathrm{pre}}}} }}{{{\mathrm{t}}_{{{\mathrm{test}}}} - {\mathrm{t}}_{{{\mathrm{PCr}}}} }} = \, \frac{{\Delta{{{\mathrm{La}}}} }}{{{\mathrm{t}}_{{{\mathrm{lac}}}} }},$$

whereas ${\dot{\mathrm{c}}}$La_max_ = maximal lactate accumulation rate; La_max_ = maximal post-exercise lactate concentration (measured every minute from the first to ninth minute post-exercise with participants remaining as still as possible); La_pre_ = lactate concentration immediately before the start of the test (that should be ≤ 1.5 mmol l^−1^ and used as an average of 2–3 samples in close temporal proximity); ΔLa = maximal increase in post-exercise lactate concentration; t_lac_ = time equivalent to account energy resynthesis from lactate accumulation; t_test_ = duration of the all-out sprint test (that should be ∼ 10–12 s) and t_PCr_ = time equivalent to account for energy resynthesis from phosphocreatine (phosphagenous time).

In other words, ${\dot{\mathrm{c}}}$La_max_ is calculated as the ratio between the maximal increase in post-exercise lactate concentration (ΔLa) and the time equivalent to account for lactic energy supply (t_lac_). It is important to note that the scientific literature started to refuse to use of *‘alactic time’* or *‘time for which no lactate formation is assumed’,* as this is barely justified by actual energy metabolism (Yang et al. 2023). Instead, Yang et al. introduced the time equivalent to account for energy resynthesis from phosphocreatine known as phosphagenous time (t_PCr_). This nomenclature is also recommended for science and practice, even though there are different methods to derive t_PCr_ experimentally (see Chapter 3.5).

Since maximal lactate accumulation rate has already been determined in N = 49 articles covering *cycling* (n = 30), *running* (n = 7), *swimming* (n = 5), *rowing* (n = 3), *isokinetic force tests* (n = 3), *handcycling* (n = 2), *kayaking* (n = 2) and *paratriathlon* (n = 1), the following chapters provide a condensed overview of their current evidence in different categories (Table 1). These categories represent the chronological order of the test procedure staring with the *Preparation* and *Equipment*, followed by *Warm-up* and *Test characteristics* and finally discussing the *Calculation* of ${\dot{\mathrm{c}}}$La_max_. In the latter, different approaches to derive/standardise t_PCr_ are discussed. By doing so, this review largely augments previous reviews that already summarised the experimental setup and reference data of n = 22 articles with all-out durations ≤ 20 s (Wackerhage et al. 2025) and n = 27 articles with seven different exercise modalities (Langley et al. 2025). However, the latter exploit a very similar structure/approach that allows for a good comparison to the findings described in this very chapter.

**Table 1:** Procedures, calculations and reference values for maximal lactate accumulation rate

Article	Sport	n	Participants	Test	Phosphagenous time (t_PCr_) [s]	Comments	${\dot{\mathrm{x}}}$ ± SD (range) [mmol l^−1^ s^−1^]
Grassi et al. (1995)	**Cycling**	**6** (f = 0)	Healthy white males	**Exercise at 200% of PPO** (5-, 15-, 25-, 35-, 45-s or until exhaustion, slope of the time–ΔL relationship, sea-level + altitude)	**Not applied**	Tests performed at sea-level (SL) as well as ∼ 1 and 4 weeks at high altitude of a 35-day sojourn at 5050 m	**0.25 ± 0.05** (SL) (0.20–0.32) **0.17 ± 0.05** (AL) (0.09–0.24)
Poffé et al. (2024)	**Cycling**	**29** (f = 10)	Professional (4), amateur (10) and recreational (17) cyclists	**15-s all-out sprint** (isokinetic @ 130 rpm, 12′ WU @ females: 1.5 or males: 2.0 W/kg)	**Individual** (t_P3.5%_)	Based on two **diploma theses** submitted in Germany in 2003 and 2004 by Weber and Kleinschmidt, respectively Used to calculate MLSS	**0.56 ± 0.15** (all) **0.50 ± 0.15** (f) **0.59 ± 0.15** (m)
Hauser et al. (2014)	**Cycling**	**13** (f = 0)	[…] with different endurance levels	**15-s all-out sprint** (isokinetic @130 rpm, 12′ WU)	**Individual** (t_P3.5%_)	Used to calculate MLSS	**0.91 ± 0.18** (0.67–1.39)
Adam et al. (2015)	**Cycling**	**23** (f = 6)	Amateur cyclists (sports students)	**15-s all-out sprint** (isokinetic @130 rpm, 12′ WU)	**Individual** t_P3.5%_ = 4.35 ± 0.72	**ICC = 0.90** (3–6 d, CV = 6%, LoA ± 0.12)	**0.70 ± 0.14** (0.35–0.98)
Manunzio et al. (2016)	**Cycling**	**4** (f = 0)	Experienced cyclists/triathletes	**15-s all-out sprint** (isokinetic @ 120 rpm,10′ WU)	**Individual** (t_Pmax_)	**Reduction** over the course of the season	**0.54 ± 0.16** (pre-training)
Wahl et al. (2017)	**Cycling**	**19** (f = 4)	Healthy/non-smoking triathletes/cyclists	**15-s all-out sprint** (isokinetic @ 120 rpm, SRM ergo.)	**Individual** (t_P3.5%_)	Used to calculate MLSS	**0.68 ± 0.12** (0.48–0.99)
Nitzsche et al. (2018a)	**Cycling/force test**	**14** (f = 0)	Trained participants from various sports	**15-s all-out sprint** (isokinetic @130 rpm, Lode ergo.) **10 all-out reps of leg flex./ext** (isokinetic @ 180° s^−1^, only left leg)	**Individual** t_P3.5%_ = 5.3 ± 0.9_C_ t_P3.5%_ = 6.7 ± 2.0_F_	**Sig. difference** between tests Non sig. correlation between tests results	**0.81 ± 0.09** (C) (0.67–0.98) **0.28 ± 0.09** (F) (0.15–0.46)
Hommel et al. (2019)	**Cycling**	**30** (f = 0)	Amateur cyclists (sport students)	**15-s all-out sprint** (isokinetic @130 rpm, 12′ WU)	**Individual** (t_P3.5%_)	**Sig. reduction** after 2 weeks of sprint interval training	**0.75 ± 0.18** (pre-training)
Quittmann et al. (2021a)	**Cycling**/**handcycling**	**18** (f = 3)	Competitive triathletes	**15-s all-out sprint** (isokinetic @130 rpm, start @ 2.0 and 0.5 N/kg in C and HC)	**Individual** t_Pmax_ = 2.87 ± 0.66_C_ t_Pmax_ = 3.05 ± 0.69_HC_	**ICC = 0.87** (C) (7 days, LoA ± 0.14) **ICC = 0.83** (HC) (7 days, LoA ± 0.11)	**0.52 ± 0.14** (C) (0.23–0.77) **0.32 ± 0.10** (HC) (0.13–0.51)
Ji et al. (2021)	**Cycling**	**10** (f = 0)	Sub-elite middle- and long-distance runners	**30-s all-out sprint** (isokinetic @ 120 rpm, 10′ WU)	**Fixed** (5.5)	Used to calculate anaerobic threshold in running	**0.39 ± 0.09** (0.31–0.55)
Dunst et al. (2023a)	**Cycling**	**9** (f = 0)	Elite track cycling sprinters	**3-, 8-, 12- + 60-s all-out sprints** (9-kg flywheel @ 120 rpm, rolling start from 20 rpm, 6-s @ ≥ 160 rpm for force–velocity profiling)	**Individual** t_PCr_ = 2.09 ± 0.41 (based on profile)	No sig. correlation to maximal pedalling frequency/cadence **Sig. positive correlation** to time constant of oxygen desaturation Desaturation kinetics reflect phosphagenous contribution	**0.95 ± 0.18** (∼ 0.88–1.40)
Dunst et al. (2023b)	**Cycling**	**9** (f = 0)	Elite track cycling sprinters	**3-, 8- + 12-s all-out sprints** (9-kg flywheel @ 120 rpm, rolling start from 20 rpm, force–velocity profiling)	**Various** t_Pmax_ = 3.57 ± 0.51 t_Ff_ = 2.18 ± 0.26	**Sig. difference** between calculation approaches Higher lactate accumulation with longer duration	∼ **0.95 ± 0.18** (∼ 0.88–1.40)
Yang et al. (2023)	**Cycling**	**30** (f = 0)	National level track cyclists	**15-s all-out sprint** (15.48 ± 0.16 s) (isokinetic @ 120 rpm,10′ WU; oxidative contribution by applying the “PCr–La^−^–O_2_ method”)	**Various** t_Pmax_ = 1.75 ± 0.59 t_P3.5%_ = 3.28 ± 1.08 t_Pmax_ + t_Oxi_ = 2.24 ± 0.84	**Sig. differences** and correlations between calculation methods Oxidative energy contribution of ∼ 3%	**0.85 ± 0.12**_Pmax_ **0.97 ± 0.18**_P3.5%_ **0.88 ± 0.13**_Pmax+Oxi_
Harnish& Miller (2023)	**Cycling**	**15** (f = 0)	Competitive male cyclists/triathletes	**15-s all-out sprint** (10′ WU @∼ 100 W, portable analyser, fingertip, Wahoo Kickr)	**Not mentioned**	Transdermal carnosine gel **fails to improve** repeated wingate performance	**0.74 ± 0.31**
Harnish et al. (2023)	**Cycling**	**30** (f = 12)	Healthy participants (18–50 yrs.)	**15-s all-out sprint** (10′ WU @∼ 30–100 W, portable analyser, fingertip, Wahoo Kickr)	**Individual** t_P3.5%_ = 4.1 ± 1.5	**ICC = 0.66** (4–7 days, CV = 19%, LoA ± 0.36)	**0.63 ± 0.24** (0.26–1.28) **0.62 ± 0.15** (f) (0.26–1.21) **0.71 ± 0.26** (m) (0.29–1.28)
Haase et al. (2024)	**Cycling**	**13** (f = 0)	Trained participants from various sports	**10-s all-out sprint** (isokinetic @ 90, 110, 130, 150 and 170 rpm; Excalibur Sport)	**Fixed** (3.0) 90: t_Pmax_ = 2.65 ± 0.58 110: t_Pmax_ = 3.42 ± 0.60 130: t_Pmax_ = 4.31 ± 0.70 150: t_Pmax_ = 5.32 ± 0.64 170: t_Pmax_ = 6.62 ± 1.15	**Differences in ΔLa:** 90 rpm: 4.38 ± 1.01 mmol/l 110 rpm: 5.35 ± 0.94 mmol/l 130 rpm: 6.02 ± 1.10 mmol/l 150 rpm: 6.14 ± 1.03 mmol/l 170 rpm: 6.57 ± 1.00 mmol/l	**0.63 ± 0.14** (90) **0.76 ± 0.13** (110) **0.86 ± 0.16** (130) **0.88 ± 0.15** (150) **0.94 ± 0.14** (170)
Langley et al. (2024)	**Cycling**	**15** (f = 0)	(well-)trained/physically active	**all-out sprint (10-, 15- and 30-s)** (with metabolic cart, 12′ WU @ 1.5 W/kg, Wattbike Pro B, 15′)	**Individual** (t_P3.5%_)	- non-significant correlations to (relative) peak power output - different energy contribution	**0.86 ± 0.17** (10) **0.68 ± 0.18** (15) **0.45 ± 0.07** (30)
Meixner et al. (2024a)	**Cycling**	**50** (f = 20)	Experienced cyclists	**15-s all-out sprint** (isokinetic @130 rpm, start @ 30 rpm, WU @ 1.5·bm for 10′)	**Various** t_Pmax_ = 2.29 ± 0.81 t_P3.5%_ = 3.40 ± 1.30 t_inter_ = 3.5	**ICC = 0.87**_Pmax_ (2–14 d, CV = 12%, LoA ± 0.14) **ICC = 0.79**_P3.5%_ (2–14 d, CV = 26%, LoA ± 0.20) **ICC = 0.91**_inter_ (2–14 d, CV = 3%, LoA ± 0.13)	**0.49 ± 0.13**_Pmax_ **0.53 ± 0.14**_P3.5%_ **0.54 ± 0.13**_inter_
Meixner et al. (2024b)	**Cycling**	**50** (f = 20)	Experienced cyclists	**15-s all-out sprint** (isokinetic @130 rpm, start @ 30 rpm, WU @ 1.5·bm for 10′)	**Fixed** (3.5)	Similar amount of work per mmol of lactate in both sexes 1 mmol/l ≈ 12 J/kg_FFM_	**0.54 ± 0.13** (all) **0.47 ± 0.09** (f) **0.58 ± 0.16** (m)
Archacki et al. (2024)	**Cycling**	**62** (f = 31)	Competitive endurance (n = 34) and speed-power athletes (n = 28)	**15-s all-out sprint** (isokinetic, Cyclus 2,’ WU + brief sprits; oxidative contribution by applying the “PCr–La^−^–O_2_ method”)	**Not applied** (3.5 for re-calculation)	Energy system contribution appears to have a **similar metabolic effect **between males and female athletes […] with similar sport-related adaptations	**0.38 ± 0.14** (f) **0.45 ± 0.07** (m) (endurance) **0.52 ± 0.08** (f) **0.58 ± 0.09** (m) (speed-power) (re-calculated)
Reinpõld et al. (2024)	**Cycling**	**32** (f = 0)	Experienced cyclists, 16 juniors (Jun), 16 seniors (Sen)	**30-s all-out sprint** (isokinetic @ 110 rpm, Cyclus 2, lactate photometer plus)	**Individual** (t_Pmax_) Jun: 2.00 ± 1.09 Sen: 2.99 ± 1.40	**Sig. neg. correlation** to mean response time and time delay of oxygen desaturation kinetics	**0.43 ± 0.08** (Jun) **0.43 ± 0.10** (Sen)
Harnish et al. (2024)	**Cycling**	**28** (f = 12)	healthy/active	**15-s all-out sprint** (10′ WU @∼ 30–100 W, portable analyser, fingertip, Wahoo Kickr)	**Various** t_Pmax_ or fixed (5.0)	**ICC = 0.47P**_max_ (4–7 days, CV = 18.1%) **ICC = 0.64**_**fix**_ (4–7 days, CV = 16.6%)	**0.60 ± 0.20**_Pmax_ **0.68 ± 0.24**_fix_
Porter & Langley (2025)	**Cycling**	**13** (f = 0)	Developmental level/trained cyclists	**All-out sprint (10-, 15- and 30-s)** (with metabolic cart, 12′ WU @ 1.5 W/kg, Wattbike Pro B, 15′)	**Individual** (t_P3.5%_) **2.56 ± 1.10** (10) **2.86 ± 1.26** (15) **2.53 ± 0.06** (30)	**Sig. high correlations** to oxygen desaturation kinetics Sig. correlation to peak power (except for the 15-s sprint)	**0.83 ± 0.15** (10) **0.67 ± 0.13** (15) **0.43 ± 0.06** (30)
Fischer et al. (2025)	**Cycling**	**36** (f = 13)	Young triathletes	**15-s all-out sprint** (isokinetic @120 rpm, WU: 10′ @ 2 W/kg, with < 3″ accel. @5–7′, seated)	**Fixed** (3.5)	Did not significantly contribute to power at lactate threshold	**0.48–0.10**
Sablain et al. (2025)	**Cycling**	**13** (f = 3)	Physically active males	**15-s all-out sprint** (isokinetic @120 rpm, WU: 10′ with < 3″ accel. @5–7′, seated)	**Individual** t_P3.5%_ = 1.17 ± 0.11	**ICC = 0.80** (4 cons. days, CV = 7% LoA ± 0.14), used to calculate MLSS	**0.48 ± 0.10**
Haase et al. (2025)	**Cycling**	**22** (f = 0)	Trained male athletes	**10-s all-out** isokinetic @130 rpm, WU @ 0.5·bm for 10′ @ 60–800 rpm)	**Fixed** (3.0)	Sprit power is **strongly associated** with ΔLa Relative metrics may enhance precision	**0.79 ± 0.10** (all) **0.83 ± 0.10** (HP) **0.75 ± 0.09** (LP)
Clark & Macdermid (2025)	**Cycling**	**11** (f = 0)	(inter-)nationally competitive endurance cyclists	**15-s all-out sprint** (isokinetic @ 130 rpm, WU: 12′ @ 1.5 W/kg, 5-s all-out, 10′ @ 50 W, portable analyser)	**Individual** (t_P3.5%_)	**Sig. correlation** to sprint power No sig. correlation to 1-min power output Beneficial for estimating respiratory compensation point	**0.52 ± 0.13** (∼ 0.36–0.77)
Meixner et al. (2025a)	**Cycling**	**25** (f = 5)	Trained cyclists/triathletes	**15-s all-out** (isokinetic @ 130 rpm, WU 10′ @ 1.5 W/kg, Cyclus 2)	**Fixed (3.5)** (used for re-calculation, not mentioned in text)	**No sig. effect of creatine** monohydrate on ΔLa with highly individual response (–1.5 to + 2.0 mmol/l)	**∼** **0.69 ± 0.19** (re-calculated)
Micke et al. (2025)	**Cycling/rowing**	**152** (f = 65)	n = 95 trained rowers (f = 50) n = 57 trained cyclists (f = 15)	**15-s all-out** (isokinetic @ 130 rpm, WU 10′ @ < 2 mmol/l, Cyclus 2) **20-s all-out sprint** (FCC, Concept II D, 10′ WU)	**Various** t_Pmax_ in cycling 4.0 in rowing	**Rowers lower **than cyclists **Males higher** than females Large effect of alternative determination approaches	**0.62 ± 0.12** (all) **0.59 ± 0.14** (f) **0.63 ± 0.11** (m) (cycling) **0.61 ± 0.12** (all) **0.59 ± 0.15** (f) **0.62 ± 0.11** (m) (cycling, pol) **0.60 ± 0.12** (all) **0.57 ± 0.13** (f) **0.61 ± 0.11** (m) (cycling, 5′) **0.30 ± 0.11** (all) **0.25 ± 0.05** (f) **0.36 ± 0.12** (m) (rowing) **0.30 ± 0.11** (all) **0.25 ± 0.06** (f) **0.35 ± 0.12** (m) (rowing, pol) **0.28 ± 0.10** (all) **0.23 ± 0.05** (f) **0.34 ± 0.12** (m) (rowing, 5′)
Quittmann et al. (2021b)	**Cycling**/**running**	**18** (f = 3)	Competitive triathletes	**15-s all-out sprint** (isokinetic @130 rpm, start @ 2.0 and 0.5 N/kg) **100-m all-out sprint** (t_test_ = 13.86 ± 1.47 s)	**Various** t_P3.5%_ = 4.44 ± 0.77_C_ t_P3.5%_ = 3.37 ± 0.54_R_ (t_Pmax_ = 2.69 ± 0.51_C_) (t_Pmax_ = 2.19 ± 0.32_R_)	**ICC = 0.90** (C) (7 days, TE = 9%, LoA ± 0.15) **ICC = 0.87** (R) (7 days, TE = 9%, LoA ± 0.16)	**0.60 ± 0.15** (C) (0.35–0.96) **0.71 ± 0.16** (R) (0.27–0.86) (t_P3.5%_-based)
Quittmann et al. (2020)	**Running**	**16** (f = 5)	Competitive runners	**100-m all-out sprint** (indoor track, standardised WU, laser velocity guard and timing lights, t_test_ = 13.86 ± 1.47 s)	**Various** t_Pmax_ = 2.08 ± 0.23 t_P3.5%_ = 3.15 ± 0.35 t_inter_ = 3.33 ± 0.15	**ICC = 0.91/0.93** (t_P3.5%_) (no/with fam., 2 days, TE = 7–8%, LoA ± 0.15) **ICC = 0.90/0.96** (t_inter_) (no/with fam., 2 d, TE = 5–7%, LoA ± 0.15/ ± 0.10)	**0.67 ± 0.16**_Pmax_ (0.24–0.84) **0.76 ± 0.20**_P3.5%_ (0.26–1.09) **0.75 ± 0.18**_inter_ (0.27–0.95)
Wawer et al. (2020)	**Running**	**73** (f = 12)	Undergraduate sports students	**8- /10- /12- /14-s all-out sprint** (Part I: an indoor track, 3 sprints per day over one t_test_ for 8 days Part II: performed for 10 and 12 s on a non-motorised treadmill [NMT] on consecutive days)	**Individual** t_Pmax_ = 1.99 ± 0.51_8_ t_Pmax_ = 2.36 ± 1.41_10_ t_Pmax_ = 2.05 ± 0.43_12_ t_Pmax_ = 2.00 ± 0.39_14_ t_Pmax_ = 2.65 ± 0.61_NMT10_ t_Pmax_ = 3.12 ± 0.29_NMT12_	**ICC = 0.89** (8, CV = 10%) **ICC = 0.82** (10, CV = 13%) **ICC = 0.92** (12, CV = 9%) **ICC = 0.84** (14, CV = 11%) **ICC = 0.76** (_NMT10_, CV = 8%) **ICC = 0.79** (_NMT12_, CV = 6%)	**0.64 ± 0.22** (8) **0.56 ± 0.19** (10) **0.60 ± 0.21** (12) **0.59 ± 0.19** (14) **0.83 ± 0.22**_NMT10_ **0.91 ± 0.18**_NMT12_
Quittmann et al. (2022b)	**Running**	**44** (f = 15)	Trained endurance atletes, 24 runner, 20 triathletes	**100-m all-out sprint** (indoor track, standardised WU, t_test_ = 13.90 ± 1.35 s, females: 15.39 ± 1.14 s, males: 13.14 ± 0.58 s)	**Interpolated** (t_inter_) 3.31 ± 0.12 (all) 3.44 ± 0.10 (f) 3.24 ± 0.05 (m)	**Sig. augmentation** of the Joyner model for 5000-m time **Sig. negative correlation** to fractional utilisation **Sig. positive correlation **to the ‘finishing kick’ (last 200 m)	**0.67 ± 0.16** (all) (0.26–1.05) **0.55 ± 0.13** (f) (0.26–0.80) **0.74 ± 0.14** (m) (0.47–1.05)
Thron et al. (2024a, b)	**Running**	**34** (15)	Adolescent/young sprinters to middle-distance runners	**100-m all-out sprint** (indoor or outdoor track, radar gun and timing lights, standardised WU, t_test_ = 13.43 ± 0.81 s in females and 11.86 ± 0.46 s in males)	**Individual** (t_Pmax_)	**Sig. correlation** to maximal sprinting speed and anaerobic speed reserve (r = 0.74) **Sig. differences** between disciplines and sexes	**0.92 ± 0.20** (100) **0.83 ± 0.16** (400) **0.71 ± 0.13** (800) **0.88 ± 0.19** (m) **0.73 ± 0.13** (f)
Wagner et al. (2024)	**Running**	**15** (f = 4)	National level Skimo athletes (Tier 3)	**80-m all-out sprint** (t_test_ = 11.5 ± 0.7 s, outdoor track, WU: 5′ easy + 2 starts of 10–15 m)	**Fixed** (3.5)	**No sig. correlations** to Skimo (sprint) performance	**0.7 ± 0.2** (0.6–0.9)
Pohl et al. (2024)	**Running**	**21** (f = 8)	Sports students	**15-s all-out sprint** (on a track, signal horn, 10′ WU incl. mobilization, activation and acceleration exercises)	**Interpolated** t_inter_ = 3.4	Acute bursts in glucose levels** do not predict** the magnitude in lactate increases **Avoid glucose containing beverages** immediately before sprint testing	**0.59 ± 0.09**_La−_ **0.51 ± 0.01**_La+_ **0.53 ± 0.10**_CHO−_ **0.54 ± 0.10**_CHO+_ **0.57 ± 0.10**_CHOa_ (0.46–0.77)
Sperlich et al. (2010)	**Swimming**	**26** (f = 13)	Competitive swimmers (9–11 years)	**100-m time trial** (t_test_ = 86 ± 10 s)	**Fixed** (2.0)	5-week crossover **intervention** (HIIT vs. HVT)	**0.05 ± 0.03** (pre-training)
Teixeira et al. (2022)	**Swimming**	**15** (f = 0)	Competitive male swimmers	**100- /200- /400-m time trial** (t_test_ = 66 ± 6, 150 ± 18, 330 ± 41 s; Ysi stationary analyser)	**Fixed** (4.0/8.0)	**No significant effect** of photobiomodulation applied prior swimming	**0.20 ± 0.05**_PBM_ **0.20 ± 0.04**_PLA_ **0.21 ± 0.04**_CON_ (100-m time trial) **0.09 ± 0.03**_PBM_ **0.08 ± 0.02**_PLA_ **0.08 ± 0.02**_CON_ (200-m time trial) **0.04 ± 0.01**_PBM_ **0.04 ± 0.01**_PLA_ **0.03 ± 0.01**_CON_ (400-m time trial)
Mavroudi et al. (2023)	**Swimming**	**14** (f = 6)	Highly-trained/elite swimmers (sprinters)	**25- /35- /50-m sprints** (t_test_ = 11.75 ± 1.38, 17.76 ± 2.04 and 26.78 ± 3,21 s, respectively; portable analyser, 50-m pool)	**Various** t_inter_ = 3.5 for t_test_ = 10–15 (+ 0.5 for t_test_ + 5) t_PCr_ = 0 t_PCr_ = 1.5 t_inter_	**Sig. correlation to speed **within each trial Highest correlation in the longest trial	**0.75 ± 0.18** (25) **0.54 ± 0.18** (35) **0.49 ± 0.16** (50) (based on t_inter_) **0.52 ± 0.11** (25) **0.39 ± 0.12** (35) **0.40 ± 0.13** (50) (based on t_PCr_ = 0)
Sengoku et al. (2024)	**Swimming**	**17** (f = 0)	Competitive/well-trained swimmers	**20-m all-out sprint** (in-water start without push-off, t_test_ = 11.5 ± 0.4 s, portable analyser, 50-m pool)	**Fixed** (3.0)	**ICC = 0.913** (4 days, n = 11) **Sig. correlation** to 50-m time and load-v-profiles	**0.63 ± 0.14**
Keller & Wahl (2025)	**Swimming**	**24** (f = 24)	Adolescent swimmers (national level)	**20-s all-out sprint** (∼ 15 min individualised WU, in-water start with push-off, acoustic and tactile signal)	**Fixed** (4.0)	**Moderate correlation** to 50-m velocity and **high correlations** to dryland strength **Negatively** associated with lactate threshold	**0.35 ± 0.12** (0.16–0.58)
Schünemann et al. (2023)	**Rowing**	**10** (f = 3)	National level rowers (U 23)	**10-s all-out sprint** (FCC, Concept II C, 100 W WU)	**Interpolated** t_inter_ = 2.95	**Sig. correlation** to glycolytic work distribution and mechanical power output	**0.45 ± 0.14** (0.25–0.66)
Held et al. (2023)	**Rowing**	**17** (f = 8)	Trained/competitive rowers	**20-s all-out sprint** (FCC, Concept II D, 10′ WU)	**Fixed** (4.0)	**ICC = 0.85** (7 days, LoA ± 0.09) R^2^ = 66% to mean power output	**0.28 ± 0.10** (all) (∼ 0.13–0.43) **0.23 ± 0.06** (f) **0.35 ± 0.10** (m)
Quittmann et al. (2018)	**Handcycling**	**12** (f = 0)	Competitive (national level) triathletes	**15-s all-out sprint** (isokinetic @140 rpm, start @ 20 Nm and 20 rpm)	**Individual** t_P3.5%_ = 2.56 ± 0.86	**Sig. positive correlation** to peak sprint power output **Sig. negative correlation** to maximal step test power	**0.45 ± 0.11** (0.27–0.63)
Quittmann et al. (2022a)	**Paratriathlon**	**1** (f = 0)	Member of the national team	**25-m all-out sprint** (S) (50-m indoor pool, floating) **15-s all-out sprint** (HC) (isokinetic @130 rpm) **110-m all-out sprint** (WR) (outdoor track, still start)	**Fixed** (3.0)	**Decrease** from 0.56 to 0.36 mmol/l/s over 2 years in handcycling	**0.52 ± 0.12** (HC)
Zwingmann et al. (2020)	**Kayaking**	**8** (f = 0)	Elite national canoe polo team players	**15-s all-out sprint** (@ FCC, kayaking ergo., 10′ WU)	**Fixed** (3.5)	Used to calculate MLSS in kayaking	**0.58 ± 0.10** (0.44–0.73)
Meixner et al. (2025b)	**Kayaking**	**15** (f = 6)	Elite/national U21 canoe polo players	**15-s all-out sprint (KE)** (@ FCC, kayak ergo., 5′ WU) **40- /50-m all-out on-water (OW)** (own kayak, fem. 40-m, 7.5 °C, fem. 17.0 ± 0.9, males 16.8 ± 1.0 s)	**Fixed** (3.5)	**Sig. higher rate** during OW (d = 0.22, LoA ± 0.30) High correlation to on-water velocity (**r = 0.84**) and moderate between procedures (**r = 0.68**)	**0.40 ± 0.16** (KE) (∼ 0.18–0.61) **0.51 ± 0.19** (OW) (∼ 0.22–0.81)
Nitzsche et al. (2018b)	**Force test**	**32** (f = 0)	Trained participants from various sports	**8/16 all-out reps of leg flex./ext** (isokinetic @ 210° s^−1^, unilateral)	**individual** t_P3.5%_ = 1.1 ± 1.5 (8) t_P3.5%_ = 5.8 ± 3.6 (16)	reliability analyses: **r = 0.72** (8, LoA ± 0.11) **r = 0.68** (16, LoA ± 11)	**0.27 ± 0.11** (8) **0.26 ± 0.07** (16)
Nitzsche et al. (2020)	**Force test**	**24** (f = 0)	Strength-trained participants	**10 all-out reps leg flex./ext. (15-s)** (isokinetic @ 180° s^−1^, only left leg)	**fixed** (3.0)	6-week parallel **intervention** (HVLL vs. LVHL)	**0.26 ± 0.09** (pre-training)

Articles are listed by sports

*AL* after 4 weeks at altitude (5050 m), *bm* body mass [kg], *C* cycling, *f* female participants, *CHOa* acute carbohydrate intake of 36 g and 500 ml preceding the warm-up, *CHO−* chronic carbohydrate intake of ≤ 1 g/kg body weight for 3 days, *CHO +* chronic carbohydrate intake of ≥ 9 g/kg body weight for 1 day, *CON* control condition, *CV* coefficient of variability, *F* based on isokinetic force tests, *FCC* freely chosen cadence, *FFM* fat-free mass, *HC* handcycling, *HIIT* high-intensity interval training, *HP* high relative peak power (> 14.09 W/kg), *HVLL* high-volume low-load training, *HVT* high-volume training, *ICC* intra-class correlation coefficient, *KE* kayak ergometer, *La−* resting lactate concentration ≤ 1.5 mmol/l, *La +* resting lactate concentration ≥ 2.5 mmol/l, *LBHL* low-volume high-load training, *LoA* limits of agreement (± 1.96 SD), *LP* low relative peak power (< 14.09 W/kg), *m* male participants, *NMT* non-motorised treadmill, *OW* on-water (kayak sprint test), *PBM* photobiomodulation, *PLA* placebo condition, *pol* polynomial-based approach, *PPO* peak power output in a graded exercise test, *R* running, *rpm* revolutions per minute, *S* swimming, *SD* standard deviation, *SL* sea-level, *TE* typical error between trials, *t*_*Ff*_ time span up to the first systematic deviation from fatigue-free force–velocity profile, *t*_*inter*_ interpolated phosphagenous time, *t*_*PCr*_ time equivalent to account for energy resynthesis from phosphocreatine (phosphagenous time), *t*_*Oxi*_ time equivalent for oxidative energy contribution, *t*_*PCr+Oxi*_ phosphagenous time added by a time equivalent for oxidative energy contribution (t_Oxi_), *t*_*Pmax*_ time to attain peak power output, *t*_*P3.5%*_ time when power output decreased by 3.5%, *t*_*test*_ period of the performed exercise test, *WR* wheelchair racing, *WU* warm-up, $\overline{x}$ mean value, *ΔLa* difference in post-exercise lactate concentration between maximum and pre-exercise lactate concentration, _*3.5%*_ based on the time when power decreased by 3.5%, _*inter*_ based on interpolated phosphagenous time, *5*′ five-minute post-exercise approach, _*Pmax*_ based on the time to attain peak power output

### Preparation

*How* should athletes *prepare* in the days/hours preceding ${\dot{\mathrm{c}}}$La_max_ testing in terms of training and nutrition? Most of the articles followed rather general guidelines of exercise testing in terms of *avoiding* strenuous workouts like high-intensity interval training (HIIT) and heavy strength training to ensure that maximal performance can be achieved. In terms of nutritional strategies, a very interesting pilot study accessed the effect of five different conditions that manipulated *acute and chronic carbohydrate availability* as well as *pre-exercise lactate concentration* (see Chapter 3.3) on ${\dot{\mathrm{c}}}$La_max_ testing in running (Pohl et al. 2024). A carbohydrate intake of ≤ 1 g/kg/day (low carb) in the 3 days preceding the test significantly reduced ${\dot{\mathrm{c}}}$La_max_ (when compared to baseline values, p < 0.05) which was probably caused by (partially) reduced glycogen stores. In contrast, following a carbohydrate-rich diet of ≥ 9 g/kg/day (high carb) on the day preceding the test did not (significantly) affect measures of ${\dot{\mathrm{c}}}$La_max_ (p = 0.08) (Pohl et al. 2024). *It can be concluded* that athletes should follow their *normal* (or slightly carbohydrate-rich) diet in the days preceding the test. Future studies should assess how different training regimes within these days affect ${\dot{\mathrm{c}}}$La_max_ and its components.

Just recently, the effect of creatine monohydrate supplementation on was examined in ${\dot{\mathrm{c}}}$La_max_ in n = 25 trained cyclists/triathletes (Meixner et al. 2025b). In this non-randomised, placebo-controlled crossover trial, participants consumed 4 daily doses of 5 g creatine monohydrate (or maltodextrin as placebo) in the 5 days preceding the sprint tests. While 15-s work significantly increased with creatine supplementation (d = 0.944), the increase in post-exercise lactate concentration (ΔLa) was unaffected (d = − 0.055). However, given the highly individual response in ΔLa (from about − 1.5 to + 2.0 mmol/l), the authors concluded that the supplementation of creatine monohydrate should (at least) be considered in conjunction with 15-s all-out sprints (Meixner et al. 2025b).

Similarly, applying photobiomodulation in advance to sprint tests in a placebo-controlled study demonstrated no effect on ${\dot{\mathrm{c}}}$La_max_ (and ${\dot{\mathrm{c}}}$La_peak_) in n = 15 male swimmers (Teixeira et al. 2022). Analogously, the effect of other ergogenic aids like *caffein, bicarbonate and nitrate* should be assessed in future studies. As for now, the use of these supplements should be *avoided* in close temporal proximity to ${\dot{\mathrm{c}}}$La_max_ testing in order to ensure standardisation between and within individuals.

### Equipment

Depending on the type of sport or—more precisely—exercise modality, different equipment is needed/used. Most frequently, all-out sprint tests are performed on a (stationary) ergometer, as applicable in (hand-)cycling, rowing, kayaking or even isokinetic (unilateral) strength testing. The ergometers used in (hand-)cycling were *Cyclus 2* (n = 15), *SRM* (n = 6), *Lode Excalibur Sport* (n = 6), as well as *Wattbike Pro* and *Wahoo Kickr* (n = 3, respectively). Recently, the use of sufficient ergometers was discussed in an *letter to the editor* (Yang et al. 2024). The authors argued that ergometers that allow *isokinetic mode should be preferred* over ergometers with linear resistances (e. g. Wattbike Pro), as these are more likely to attain high cadences (120–130 rpm) and maximal power output which is considered necessary for ‘traditional’ determinations of t_PCr_ (see Chapter 3.5). Given that even linear resistance ergometers may still attain maximal cadences of > 130 rpm (Langley et al. 2024), it should—in this instance—*not* be a major concern. To ensure immediate force transmission on the pedals, the use of cycling-specific cleated shoes was recommended over the mere use of straps (Yang et al. 2024). However, differences in terms of reliability have not yet been investigated.

Despite the use of appropriate ergometers, a suitable *lactate analyser* (most frequently Biosen C-Line) is crucial for ${\dot{\mathrm{c}}}$La_max_. It was recently show that even stationary analyser may display average differences of − 32% (Biosen vs. Ysi) that may be corrected in post by using adequate regressions (Mentzoni et al. 2024). This concrete example affects the results of a previous study, that reported quite low values of ${\dot{\mathrm{c}}}$La_max_ (Teixeira et al. 2022). Whereas portable analyser are affordable and handy tools to assess lactate intensity domain during training sessions, their precision was found to be around ± 0.4 mmol/l (Mentzoni et al. 2024). Hence, using these devices for exercise testing will (probably) decrease reliability and long-term interpretation of ${\dot{\mathrm{c}}}$La_max_ measurements (Harnish et al. 2023). However, using a portable analyser did not hinder Japanese colleagues to demonstrate excellent reliability (ICC = 0.913) in swimming when the average value of two analysers was used (Sengoku et al. 2024). With this procedure, even portable analysers seem to reliably estimate ${\dot{\mathrm{c}}}$La_max_. In the *field* (e. g. track or pool), portable analysers might be a convenient augmentation to check whether La_pre_ is ≤ 1.5 mmol/l when *actual* blood samples are analysed *later* in the lab. To summarise, a *reliable ergometer and (stationary) lactate analyser are mandatory* to measure ${\dot{\mathrm{c}}}$La_max_ at an appropriate level of precision.

### Warm-up

It was recently shown that consuming 500 ml of glucose-containing beverage (36 g of carbohydrates) ∼ 5 min before starting the warm-up may increase La_pre_ (p < 0.05) in the following sprint test without significantly affecting ${\dot{\mathrm{c}}}$La_max_ (Pohl et al. 2024). However, given the high inter-individual variability, it is recommended to *avoid* acute carbohydrate intake ∼ 30 min preceding the sprint.

Reporting of the performed warm-up in scientific literature ranged from very precise/detailed descriptions (Pohl et al. 2024) to articles without *any* specification (Sperlich et al. 2010; Wahl et al. 2017; Nitzsche et al. 2018a, 2020; Grassi et al. 1995). An overview of all warm-up specifications is provided in the Supplementary Material (Appendix 3). In the earliest attempts of quantifying ${\dot{\mathrm{c}}}$La_max_, cycling for 12 min at an intensity of 1.5 (females) or 2.0 W/kg (males) followed by a passive rest for 10 min was performed before ${\dot{\mathrm{c}}}$La_max_ testing (Poffé et al. 2024). In order to *activate* the recruitment of larger motor units and *prime* phosphagenous and glycolytic metabolism (MacIntosh et al. 2000; Tomaras & MacIntosh 2011; Ozkaya 2013), researchers started to implement short bursts of high-intensity in in (hand-)cycling and running warm-ups in three different ways: Either by performing a *single sprint* of 3–6 s in cycling (Adam et al. 2015; Hommel et al. 2019; Dunst et al. 2023a, b; Langley et al. 2024; Porter & Langley 2025; Clark & Macdermid 2025), *several accelerations within* the warm-up (Wawer et al. 2020; Quittmann et al. 2021a; Pohl et al. 2024; Sablain et al. 2025) or *2–3 maximal starts in the end* (5–15 m) of the warm-up (Quittmann et al. 2020, 2021b, 2022a; Wagner et al. 2024; Archacki et al. 2024).

Interestingly, ${\dot{\mathrm{c}}}$La_max_ warm-ups in swimming, rowing and kayaking were exclusively applied at low-intensity or in an individualised fashion. Aside from the warm-up itself, the implementation of active/passive rest preceding the sprint ranged from 1 to 10 min. From anecdotal evidence of testing sprinters in running, we observed that performing three maximal starts may lead to lactate concentrations ≥ 4 mmol/l (especially in highly glycolytic athletes) which may not be ≤ 1.5 mmol/l after only 5 min of passive recovery (Taoutaou et al. 1996). Hence, implementing several accelerations/starts in the warm-up is recommended as long as active/passive rest can be extended until La_pre_ ≤ 1.5 mmol/l. However, the effect of different types of warm-ups on ${\dot{\mathrm{c}}}$La_max_ (and its components) has not yet been examined systematically. Hence, scientific colleagues are *encouraged* to compare low-intensity only warm-ups against 2–3 accelerations as well as high-intensity warm-ups. However, La_pre_ should still be matched to follow the common criterion of ≤ 1.5 mmol/l (Pohl et al. 2024). Aside from the determination of ${\dot{\mathrm{c}}}$La_max_, the participants’ perceived *‘readiness’* to sprint should be another criterion. The author assumes that 2–3 accelerations/sprints of high- or sprint-intensity lead to higher perceived *‘readiness’* with marginal effects on ${\dot{\mathrm{c}}}$La_max_ determination.

### Test characteristics

In contrast to performing all-out sprint tests to determine the maximal rate of blood lactate accumulation, the first experimental approach made use of the relationship between exercise duration and post-exercise lactate concentration at a fixed intensity of two-times peak power output (Grassi et al. 1995). Due to the (almost) linear relationship between exercise times (ranging between 5 and > 45 s) and lactate concentration, the slopes of the individual regressions were identified as ${\dot{\mathrm{c}}}$La_max_. As such, the approach of Grassi et al. (1995) is the only example that did not apply any variant of Eq. (1). Accordingly, mean values in their experiments attained 0.25 ± 0.05 mmol/l/s at sea-level in n = 6 participants and were significantly affected by altitude. Around the same time, Alois Mader suggested to apply all-out sprints of 10–15 s durations to determine ${\dot{\mathrm{c}}}$La_max_, as recently highlighted in another review (Wackerhage et al. 2025). These have been performed for > 2 decades as a practical and time-efficient tool.

In general, sprint test for determining ${\dot{\mathrm{c}}}$La_max_ can be characterised by their application of a *standardised time, distance or number of repetitions*. Depending on exercise modality, the usage of these applications varies. Whereas testing in (hand-)cycling, rowing and kayaking exclusively applied a fixed *time* (10–30 s), ${\dot{\mathrm{c}}}$La_max_ tests in running, swimming and paratriathlon applied *both*—a fixed time and distance. A fixed number of *repetitions* (8–16 reps) was exclusively applied in isokinetic force tests (Nitzsche et al. 2018a, b, 2020). Even though fixed-time and -distance approaches have demonstrated high reliability (see Chapter 4), the choice between these two methods affects the *measurement, calculation and interpretation* ${\dot{\mathrm{c}}}$La_max_. It was found that glycolytic energy contribution is ∼ 25 ± 7% in 100-m sprint running (Park et al. 2021) and ∼ 35 ± 7% in 15-s all-out cycling (Yang et al. 2023). To put it in a nutshell for cycling: *“When considering the methods used for maximal sprints on a cycle ergometer, there are differences in test mode (isokinetic, non-isokinetic with fixed load/breaking force), test duration (10 s, 15 s, 30 s) and pedaling frequency (120 rpm or 130 rpm in isokinetic mode, maximal in non-isokinetic mode) […]”* (Haase et al. 2024). Hence, researches and practitioners should be *aware* of these aspects and select their procedures accordingly with respect to *accuracy and feasibility*.

In a recent review, a total of six problems were highlighted with regards to ${\dot{\mathrm{c}}}$La_max_ testing (Wackerhage et al. 2025). Firstly, the authors mention that PFK (and hence glycolysis) are unlikely to be fully activated during all-out exercise in vivo. Secondly, measuring post-exercise lactate concentration will underestimate the *‘true’* maximal rate of lactate accumulation. Thirdly, these authors argue that there is no criterion (analogous to levelling-off in ${\dot{\mathrm{V}}\mathrm{O}}$_2max_ testing) to indicate whether ${\dot{\mathrm{c}}}$La_max_ has been reached. Fourthly, lactate clearance is not taken into consideration when determining ${\dot{\mathrm{c}}}$La_max_ making it only a net measure of lactate accumulation. Fifthly, in (most of) the tests, oxidative phosphorylation and energy contribution is not considered. And lastly, accounting for a certain t_PCr_ appears to be individual and error-prone (Wackerhage et al. 2025). As the authors *“see no way to resolve the six problems”*, they recommend to call this experimental parameter a *‘peak’*, but not a *‘maximal’* rate. However, this seems to be a matter of perspective. With regards to the cellular processes underlying muscle contraction, there seem to be fundamental problems when analysing post-exercise whole-body lactate concentration. But only within the field of *experimental* (whole-body) setting, one could argue that—under the given possibilities and with all the empirical limitations—a *maximal* rate refers to the best possible estimate, which is why ${\dot{\mathrm{c}}}$La_max_ is used thoughout this article.

#### Test duration/distance

Test duration or distance should be rather short to *avoid* an inflated *oxidative contribution* and/or *pH-dependent PFK suppression* (Heck et al. 2003; Wackerhage et al. 2022, 2025). The 15-s all-out sprint test is the by far most frequently used duration (n = 28) even though several (n = 6) studies used a 10-s sprint as recommended > 20 years ago by Heck et al. (2003) (Wawer et al. 2020; Schünemann et al. 2023; Haase et al. 2024; Langley et al. 2024; Porter and Langley 2025; Haase et al. 2025). In cycling, comparing sprint test durations of 10, 15 and 30 s demonstrated significantly different outcomes in ${\dot{\mathrm{c}}}$La_max_ that were highest in the 10-s all-out (Langley et al. 2024; Porter & Langley 2025). Whereas a significant difference between 10- (0.86 ± 0.17 mmol/l/s) and 30-s sprinting (0.45 ± 0.07 mmol/l/s) appeared to be predictable (d = 3.15), a surprisingly high difference (d = 1.07) was also observed between ${\dot{\mathrm{c}}}$La_max_ in 10- and 15-s (0.68 ± 0.18 mmol/l/s) all-out sprints (Langley et al. 2024). These findings were closely replicated in a follow-up study of the same group (Porter & Langley 2025).

Similarly in swimming, ${\dot{\mathrm{c}}}$La_max_ derived from various sprint test *distances* differed significantly between 25- (0.75 ± 0.18 mmol/l/s), 35- (0.54 ± 0.18 mmol/l/s) and 50-m (0.49 ± 0.16 mmol/l/s) all-outs, even though a *portable* analyser was used (Mavroudi et al. 2023). In contrast, Corinna Wawer et al. (2020) demonstrated rather *low* differences in ${\dot{\mathrm{c}}}$La_max_ between 8- (0.64 ± 0.22 mmol/l/s), 10- (0.56 ± 0.19 mmol/l/s), 12- (0.60 ± 0.21 mmol/l/s) and 14-s (0.59 ± 0.19 mmol/l/s) sprint running on the track that attained a similar (good to excellent) reliability (ICC = 0.82–0.92) in undergraduate sports students. Whereas the highest ${\dot{\mathrm{c}}}$La_max_ was observed in the 8-s sprint, reliability was best in the 12-s sprint (Wawer et al. 2020). Similarly in isokinetic force tests, performing 8 (0.27 ± 0.11 mmol/l/s) or 16 repetitions (0.26 ± 0.07 mmol/l/s) resulted in a similar ${\dot{\mathrm{c}}}$La_max_ at a similar reproducibility (limits of agreement ± 0.11 mmol/l/s) (Nitzsche et al. 2018a).

*In the light of these findings,* previous measurements obtained from swimming time trials of 100 (Sperlich et al. 2010; Teixeira et al. 2022) or even 200 and 400 m (Teixeira et al. 2022) as well as 30-s all-out cycling (Ji et al. 2021; Reinpõld et al. 2024) should *clearly* be considered as $\dot{c}$*La*_*peak*_ rather than ${\dot{\mathrm{c}}}$La_max_. Similar considerations have to be made when using 20-s all-out sprint tests (Held et al. 2023; Keller & Wahl 2025; Micke et al. 2025) that appear to be *too long* for determining ${\dot{\mathrm{c}}}$La_max_.

*In summary,* recent findings indicate that ${\dot{\mathrm{c}}}$La_max_ should be derived from all-out sprint tests lasting *10–12 s* by using a *fixed-duration or -distance* approach. Noteworthy, this is in accordance with another review on methodology (Langley et al. 2025). This may ensure that maximal glycolytic contribution is attained while limiting the influence of pH-dependent inhibition and increased oxidative contribution. Performing sprint tests ≥ 15 s is *not* recommended and should be accompanied by the label ${\dot{\mathrm{c}}}$La_peak_ (instead of ${\dot{\mathrm{c}}}$La_max_). However, this is in contrast to the nomenclature recommended by Wackerhage et al. (2025) who applied to suffix *‘peak’ “to denote that this is the measured peak rate but not necessarily the maximal possible rate”* (Wackerhage et al. 2025). Depending on the respective exercise modality, this still offers quite a variety of possibilities for practitioners in exercise testing. In order to *improve* standardisation between participants and investigations, *fixed-time approaches are preferred* over fixed-distance approaches.

#### Test mode

Whereas the vast majority of studies (n = 25) used *isokinetic* test mode (most frequently at 120 or 130 rpm) for detecting ${\dot{\mathrm{c}}}$La_max_ in (hand-)cycling, some (n = 7) articles did not apply this mode (Dunst et al. 2023a, b; Harnish et al. 2023, 2024; Langley et al. 2024; Porter & Langley 2025; Harnish & Miller 2023). This is of particular interest as the effect of pedalling frequency on ${\dot{\mathrm{c}}}$La_max_ (and its components) has extensively been examined in (n = 14) trained participants (Haase et al. 2024). The participants performed isokinetic 10-s all-out sprints tests at *five* different cadences (90, 110, 130, 150 and 170 rpm) in randomised order. The time course of post-exercise-lactate concentration was modelled by a bi-exponential 3-parameter model (Haase et al. 2024). La_pre_ was similarly low between conditions (∼ 0.80–90 mmol/l) which might be due to the fairly low warm-up intensity (10 min at 0.5 W/kg). For higher pedalling frequencies, peak and mean power decreased significantly (from 1157 ± 164 to 776 ± 340 W and 748 ± 121 to 351 ± 178 W, respectively), whereas an increase in ${\dot{\mathrm{c}}}$La_max_, La_max_, ΔLa, and the time to reach peak power (t_Pmax_) maximal La_max_ was observed (Haase et al. 2024). However, the numerically highest peak power output (1212 ± 185 W) was observed in the 110 rpm condition (Haase et al. 2024).

Assuming a fixed t_PCr_ of 3 s, the reported ${\dot{\mathrm{c}}}$La_max_ differed numerically between 90 (0.63 ± 0.14 mmol/l/s), 110 (0.76 ± 0.13 mmol/l/s), 130 (0.86 ± 0.16 mmol/l/s), 150 (0.88 ± 0.15 mmol/l/s) and 170 rpm (0.94 ± 0.14 mmol/l/s). Since average differences between conditions were remarkably lower at ≥ 130 rpm, Haase et al. (2024) concluded that *“pedalling frequencies of at least 130 rpm or higher are necessary to reach”* ${\dot{\mathrm{c}}}$La_max_. Since most (n = 19) previous articles in (hand-)cycling already applied a cadence of ≥ 130 rpm and only a few studies (n = 7) used a pedalling frequency of 120 (Manunzio et al. 2016; Wahl et al. 2017; Ji et al. 2021; Yang et al. 2023; Sablain et al. 2025; Fischer et al. 2025) or 110 rpm (Reinpõld et al. 2024), it seems that research mostly follows this recommendation. However, these findings are hardly relevant for running, swimming, rowing or kayaking, in which the number and frequency of steps/strokes cannot be standardised.

Another aspect of test mode—that is way less discussed in the literature—is the exact *starting procedure* of the sprint test. First, it has to be *reported* whether the participants demonstrate a *‘rolling start’* (pedals already moving) (Dunst et al. 2023b; Sablain et al. 2025; Haase et al. 2024) or if they start from a *still* position. Aside from that, the ergometer’s power measurement can start *immediately* (Quittmann et al. 2021a, b) or *when a certain cadence* of 20 (Quittmann et al. 2018) or 30 rpm (Meixner et al. 2024a, b) *is exceeded*. This is particularly important when starting from a still position as there might be a duration of ∼ 1.0–1.5 s in which power equals zero but test time is already elapsing. This should be accounted for by adjusting t_test_ and subtracting the time of zero values. Since this seems quite inconvenient, starting the measurement at a low-cadence (if possible ≥ 1 rpm) is recommended for starting in a still position. Lastly, the *initial resistance* of the all-out sprint test is barely reported but *should be considered* as it might influence the power-time curve which is particularly important when using t_PCr_ = t_Pmax_ (Martin et al. 1997). Previous articles using the Cyclus 2 ergometer reported an initial resistance of 2.0 N/kg (Quittmann et al. 2021a, b) or 0.085 and 0.075 kg per kg body weight (Archacki et al. 2024) (males and females, respectively) in cycling, whereas 20 N (Quittmann et al. 2018) and 0.5 N/kg (Quittmann et al. 2021a) have been reported in handcycling. Future research could examine the effect of initial resistance on t_Pmax_ and ${\dot{\mathrm{c}}}$La_max_ and examine its interaction with pedalling frequency.

#### Blood sampling

Since ${\dot{\mathrm{c}}}$La_max_ is mainly derived from post-exercise lactate measurements (Eq. 1), the consideration of appropriate blood sampling is *crucial*. Aside from using an appropriate analyser (see Chapter 3.2), the *site* of blood samples should be considered. Whereas most studies collected blood samples from the *earlobe*, some (n = 9) articles reported that the used the participants’ *fingertip* (Mavroudi et al. 2023; Harnish et al. 2023, 2024; Langley et al. 2024; Reinpõld et al. 2024; Sengoku et al. 2024; Porter & Langley 2025; Sablain et al. 2025; Harnish & Miller 2023). With the only advantage that athletes can test themselves, collecting blood samples from the fingertip is *not* recommended as it is way more sensitive (hurts more), more prone to sweat contamination, increases the likelihood of bloodstains in the lab (worse hygiene) and results in higher and less reliable measures of lactate concentration (Zhong et al. 2024).

*Occasion* of blood sampling seem to be quite heterogeneous across studies (see Table 2) which was already highlighted in a previous review (Langley et al. 2025). A differentiation of lactate concentration at arrival and after warm-up was reported in n = 8 articles (Quittmann et al. 2018, 2020, 2021a, b, 2022a, b; Wagner et al. 2024; Pohl et al. 2024). Even though these measurements (typically) are not used for calculating ${\dot{\mathrm{c}}}$La_max_, these may be of particular interest in future studies examining the effect of different warm-ups. Since La_pre_ demonstrates insufficient reliability when collected as a single sample (Quittmann et al. 2020, 2021a, b; Harnish et al. 2023; Sablain et al. 2025), several studies used the average of two (Hauser et al. 2014; Hommel et al. 2019; Langley et al. 2024; Meixner et al. 2024a, b, 2025a, b; Sengoku et al. 2024; Porter & Langley 2025; Clark & Macdermid 2025) or even three (Poffé et al. 2024) blood samples that were collected in close temporal proximity. Interestingly, one study in swimming managed the applied double-sampling *throughout* pre- and post-exercise with a portable analyser (Sengoku et al. 2024).

**Table 2:** Blood sampling occasions among peer-reviewed articles and recommendations

Article	Pre-exercise			Post-exercise [min]																					
	Ar	WU	Pre	0	0.5	1	1.5	2	2.5	3	4	5	6	7	8	9	10	11	12	13	14	15	20	25	30
Grassi et al. (1995)			×			×				×		×		×											
Poffé et al. (2024)			×××	×		×		×		×	×	×	×	×	×	×	×								
Sperlich et al. (2010)			×			×				×		×		×			×								
Hauser et al. (2014)			××	×		×		×		×	×	×	×	×	×	×									
Adam et al. (2015)			×	×		×		×		×	×	×	×	×	×	×									
Manunzio et al. (2016)			×			×		×		×	×	×	×	×	×	×	×								
Wahl et al. (2017)			×			×		×		×	×	×	×	×	×	×	×								
Nitzsche et al. (2018a, b)			×	×	×	×	×	×	×	×	×	×	×	×	×	×									
Quittmann et al. (2018)	×	×	×	×		×		×		×	×	×	×	×	×	×	×								
Hommel et al. (2019)			××	×		×		×		×	×	×	×	×	×	×									
Quittmann et al. (2020)	×	×	×	×		×		×		×	×	×	×	×	×	×	×								
Nitzsche et al. (2020)			×	×	×	×	×	×	×	×	×	×	×	×	×	×									
Quittmann et al. (2021a, b)	×	×	×	×		×		×		×	×	×	×	×	×	×	×								
Zwingmann et al. (2020)			×			×		×		×	×	×	×	×	×	×	×								
Wawer et al. (2020)			×			×		×		×	×	×	×	×	×	×	×								
Ji et al. (2021)			×			×		×		×	×	×	×	×	×	×	×								
Quittmann et al. (2022a, b)	×	×	×	×		×		×		×	×	×	×	×	×	×	×								
Teixeira et al. (2022)			×			?				?		?		?		?		?		×		×			
Dunst et al. (2023a, b)			×	×		×				×		×		×			×					×	×	×	×
Schünemann et al. (2023)			×			×		×		×	×	×	…												
Yang et al. (2023)			×			×		×		×	×	×	×	×	×	×	×								
Mavroudi et al. (2023)*#			×		×		×		×	×	×	×	…												
Harnish & Miller (2023)*#			×			×				×		×		…											
Held et al. (2023)			×	×		×		×		×	×	×	×	×	×	×	×	×	×	×	×	×			
Harnish et al. (2023)*#			×			×				×		×		×		…									
Haase et al. (2024)+			×	×	×	×	×	×	×	×	×	×	×	×	×	×	×	×	×	×	×	×	×	×	×
Thron et al. (2024a, b)			×			×		×		×	×	×	×	×	×	×	×								
Wagner et al. (2024)	×	×××	×	×		×		×		×	×	×	×	×	×	×	×								
Langley et al. (2024)*			××			×		×		×	×	×	×	×	×	×	×	×	×	×	×	×			
Meixner et al. (2024a, b)	×		××	×		×		×		×	×	×	×	×	×										
Reinpõld et al. (2024)*#			×			×		×		×	×	×	…												
Archacki et al. (2024)			×							×	×	×	×	×	×	×	×								
Pohl et al. (2024)	×	×	×			×		×		×	×	×	×	×	×	×									
Sengoku et al. (2024)*#			××			××		××		××	××	××	…												
Harnish et al. (2024)*#			×			×				×		×		×		…									
Porter & Langley (2025)*			××			×		×		×	×	×	×	×	×	×	×	×	×	×	×	×			
Meixner et al. (2025a, b)		×	××	×		×		×		×	×	×	×	×	×										
Fischer et al. (2025)			×					×		×	×	×	×	×	×	×									
Keller & Wahl (2025)			×	×		×		×		×	×	×	×	×	×	×									
Sablain et al. (2025)*			×			×		×		×	×	×	×	×	×	×									
Haase et al. (2025)			×	×		×				×		×		×		×			×						
Clark & Macdermid (2025)#			××			×		×		×	×	×	…												
Micke et al. (2025)			×			(×)		(×)		×	(×)	×	(×)	×	(×)	×	(×)								

	A	WU	pre	0	0.5	1	1.5	2	2.5	3	4	5	6	7	8	9	10	11	12	13	14	15	20	25	30
Recommended (×)/optional (×)	×	×	**××**×	×		**×**		**×**		**×**	**×**	**×**	**×**	**×**	**×**	**×**	**×**	…							

Recommended (n = 12) and optional (n = 4) sampling occasions are highlighted in bold and underline, respectively

*A* at arrival, *pre* number of pre-exercise blood sampling (average), *WU* immediately after the warm-up

^*^Blood samples were taken from the fingertip (instead of earlobe)

^#^A portable analyser was used

…Until a decline in post-exercise lactate concentration was observed

^+^Blood samples were collected every 30 s up to 9 min, every minute up to the 15 min, every 2 min until 21 min and every 3 min up to 30 min to model lactate kinetics

Post-exercise lactate concentration was most frequently determined every minute for 8–10 min starting immediately or 1 min after performing the all-out sprint, whereas (n = 9) studies collected blood samples every 2 min (Sperlich et al. 2010; Dunst et al. 2023a, b; Grassi et al. 1995; Teixeira et al. 2022; Harnish & Miller 2023; Harnish et al. 2024; Haase et al. 2025; Micke et al. 2025). Some studies (n = 5) terminated blood samples when a decrease in lactate concentration was observed (Schünemann et al. 2023; Mavroudi et al. 2023; Reinpõld et al. 2024; Sengoku et al. 2024; Clark & Macdermid 2025; Harnish & Miller 2023; Harnish et al. 2024). In contrast, some studies collected blood samples for as long as 15 (Held et al. 2023; Langley et al. 2024; Porter & Langley 2025; Teixeira et al. 2022) or even 30 min post-exercise (Dunst et al. 2023a, b; Haase et al. 2024), which might be applied for modelling lactate kinetics. In that regard, Haase et al. (2024) performed the most extensive analysis with a total of *30 post-exercise blood samples*, which may require additional ethical approval.

Just recently, traditional blood sampling (every minute) was compared against a poliomial interpolation (based on samples every other minute) with a single blood sample collected 5 min post-exercise in both cycling and rowing (Micke et al. 2025). A strong correlation between methods and a significant underestimation of the 5-min approach (− 0.02 ± 0.02) was observed whereas the polynomial approach demonstrated no significant difference to the traditional approach (Micke et al. 2025). However, given the rather large limits of agreement (± 0.04 and ± 0.06 mmol/l/s, respectively), these alternative approaches should be applied with caution and still need to be analysed in terms of reliability.

*In summary,* pre- and post-exercise blood sampling for determining ${\dot{\mathrm{c}}}$La_max_ should be performed with respect to the aims of the investigation. Even though *stationary* analysers are recommended, averaging the results of two portable analysers may resolve in sufficient reliability. Whereas *arrival* and *warm-up* measurements appear optimal, collecting *2–3 blood samples for La*_*pre*_ is recommended. Since the time to reach La_max_ was found to range from 1 to 9 min (Mavroudi et al. 2023; Held et al. 2023; Harnish et al. 2023), a sampling rate of (at least) *one blood sample per minute until 10 min* is recommended. In highly glycolytic athletes (who might demonstrate an extended time to reach La_max_), researchers may continue blood sampling *until a decline in lactate concentration is observed*. Future studies may explore the time to reach La_max_ in various sports and athletes.

### Calculation

Aside from differing test characteristics, previous research developed different calculation approaches to determine ${\dot{\mathrm{c}}}$La_max_ that mainly focus on its (by far) most discussed component—the method used to define t_PCr_ (Eq. 1). Since a change in t_PCr_ by 1 s can alter ${\dot{\mathrm{c}}}$La_max_ from a 15-s all-out sprint by as much as *26%* (Dunst et al. 2023a), it is *important to consider* this—admittedly controversial—parameter. As highlighted in Table 1, empirical studies used different methods for t_PCr_ ranging from 0 to 6.7 ± 2.0 s (Mavroudi et al. 2023; Nitzsche et al. 2018b). These methods could be characterised as *individual, fixed or interpolated*. The most frequently applied *individual* methods to define t_PCr_ are the *time when power output decreased by 3.5%* (t_P3.5%_, n = 16) or t_Pmax_ (n = 12). Especially in exercise modalities that do *not* allow for simultaneous power measurements, *fixed* and *interpolated* methods might be handy and typically refer to previous simulation approaches assuming a linear relationship between t_test_ and t_PCr_ (Heck et al. 2003). Accordingly, t_PCr_ can be *interpolated* (t_inter_) by the rather *simplified* (Eq. 2):

2 $${\mathrm{t}}_{{{\mathrm{PCr}}}} {\text{ = t}}_{{{\mathrm{inter}}}} \approx \frac{{{\mathrm{t}}_{{{\mathrm{test}}}} }}{10} + { 2,}$$

whereas t_inter_ = interpolated phosphagenous time; t_PCr_ = time equivalent to account for energy resynthesis from phosphocreatine (phosphagenous time); t_test_ = duration of the all-out sprint test (should be ∼ 10–12 s). This equation results in estimates of t_PCr_ between 3.0 and 3.5 s for t_test_ between 10 and 15 s, respectively.

To date, a *fixed/interpolated* t_PCr_ has become the most frequently applied method (n = 25) especially aside from cycling. Importantly, the reliability of ${\dot{\mathrm{c}}}$La_max_ was found to be *affected* by the method used to define t_PCr_ when comparing t_inter_ (ICC = 0.911), t_Pmax_ (ICC = 0.866) and t_P3.5%_ (ICC = 0.794) in cycling (Meixner et al. 2024a). In contrast, Adam et al. (2015) demonstrated an excellent reliability for ${\dot{\mathrm{c}}}$La_max_ (ICC = 0.904) when using t_P3.5%_ (Adam et al. 2015). When using fingertip blood samples and a portable analyser, fixed approaches demonstrated better reliability (ICC = 0.64) when compared to t_Pmax_ (ICC = 0.47) (Harnish et al. 2024).

In running, the reliability of ${\dot{\mathrm{c}}}$La_max_ was found to be even higher when using t_inter_ (ICC = 0.960) in comparison to t_P3.5%_ (ICC = 0.928) while resulting in similar/excellent outcomes (Quittmann et al. 2020). In swimming, three versions of t_PCr_, namely t_inter_, 1.5 × t_inter_ and t_PCr_ = 0, resulted in significant differences in ${\dot{\mathrm{c}}}$La_max_ (${\dot{\mathrm{c}}}$La_peak_) attained in 25-, 35- and 50-m sprints (Mavroudi et al. 2023). When using t_PCr_ = 0, correlations to swimming speed over 25 and 35 m as well as between ${\dot{\mathrm{c}}}$La_max_ in these three sprints remained non-significant. However, the authors argued that the *“results were qualitatively the same”* and *“reasonable variations in [t*_*PCr*_*] do not impact considerably on the value of [*$\dot{c}$*La*_*max*_*]”* (Mavroudi et al. 2023).

Oxidative energy contribution during 15-s all-out cycling (∼ 3%) was accounted for by colleagues from South Korea who developed an adjusted formula (Yang et al. 2023). They compared ${\dot{\mathrm{c}}}$La_max_ derived from t_P3.5%_ (0.97 ± 0.18 mmol/l/s) and t_Pmax_ (0.85 ± 0.12 mmol/l/s) to—what they called—‘pure’ ${\dot{\mathrm{c}}}$La_max_ (0.88 ± 0.13 mmol/l/s) by adding an oxidative time equivalent (3.13 ± 1.61%) to t_PCr_ and demonstrated significant differences (Yang et al. 2023). Since ‘pure’ ${\dot{\mathrm{c}}}$La_max_ was highly correlated with ${\dot{\mathrm{c}}}$La_max_ derived from t_Pmax_ (r = 0.99, p < 0.001) and the latter demonstrating the highest relationship with mean power output (r = 0.48, p = 0.008), the authors concluded that oxidative contribution can be accounted for, but using t_Pmax_ *“might be recommended for a faster analysis of the practical approach in the field”* (Yang et al. 2023). Since oxidative energy contribution was found to by higher in 100-m sprints (> 10%), it would be interesting to replicate this study in running as differences in ${\dot{\mathrm{c}}}$La_max_ are likely to be higher between t_PCr_ approaches (Park et al. 2021).

A novel approach to determine t_PCr_ was introduced by Anna Katharina Dunst et al. (2023a) who combined force–velocity and power–velocity profiling with calculations of post-exercise lactate kinetics. They argued that the use of t_Pmax_ and/or t_P3.5%_ as indicators of a beginning *‘fatigue’* (phosphagenous metabolism already declining) seems reasonable at first, but may lack validity, as these times highly depend on the test conditions (e. g. resistance and pedalling frequency) (Dunst et al. 2023a). For example, a *low* initial resistance results in a *short* t_Pmax_, whereas a *high* resistance *delays* t_Pmax_ (Martin et al. 1997). To quote: *“Defining the end of the fatigue-free period on [t*_*Pmax*_*] may result in either underestimation or overestimation”* (Dunst et al. 2023a). In order to overcome this issue, the authors proposed the *first systemic deviation from a (fatigue-free) force–velocity profile (t*_*Ff*_*)* to be a more appropriate option. Force velocity profiles derived from 3 maximal isokinetic (120 rpm) sprints (3, 8 and 12 s) that were separated by ∼ 2 h as well as a 6-s maximal rate (≥ 160 rpm) low-resistance test that was performed 30 min afterwards (Dunst et al. 2023a). Dunst et al. (2023a) found that t_Ff_ (< 3 s) was significantly lower than t_Pmax_ (≥ 3 s, d = − 2.11, p < 0.001). In contrast to t_Pmax_, t_Ff_ correlated negatively with ΔLa following a 3-s all-out (r = − 0.732, p < 0.007) indicating its sensitivity for changes in lactate production—even at *very short* durations (Dunst et al. 2023a). The authors recommended that protocols to determine ${\dot{\mathrm{c}}}$La_max_ should include a short (≤ 6 s) sprint test with very low resistance and a maximal sprint (≤ 12 s) associated with a linear reduction in mean pedal force over time (Dunst et al. 2023a). Even though the reliability of this approach has to be assessed in future studies, it seems *promising* for professional cyclists who aim to apply force–velocity profiles *aside* from determining ${\dot{\mathrm{c}}}$La_max_.

*In conclusion,* the method used to define t_PCr_ matters with respect to the *reliability, applicability and interpretability* of ${\dot{\mathrm{c}}}$La_max_. As individual method, *t*_*Pmax*_* should be preferred over t*_*P3.5%*_ as it demonstrates *superior* reliability and correlations to peak power and is *not* related to the (in fact inaccurately cited) measurement error of a certain ergometer. Fixed/interpolated methods typically result in more reliable outcomes of ${\dot{\mathrm{c}}}$La_max_ and are recommended in exercise modalities that do *not* rely on power measures. Depending on the test mode (fixed-duration vs. -distance) t_inter_ can be calculated according to Eq. 2. However, given that phosphagenous contribution can only *roughly* be approximated (Hirvonen et al. 1987; Gastin 2001) and lactate accumulation starts *immediately* after starting exercise (Gastin 2001; Brooks et al. 2021), future research should consider to *refrain* from t_PCr_ and simply divide ΔLa by t_test_ in empirical settings. According to a previous review, this calculation should reduce values for ${\dot{\mathrm{c}}}$La_max_ by ∼ 28% (Wackerhage et al. 2025).

## Reliability

Previous reviews on the reliability of ${\dot{\mathrm{c}}}$La_max_ found that this parameter demonstrates poor/moderate to excellent reliability even though only n = 5 (Fernandez-Jarillo and Lomero-Arenas 2025) and n = 8 articles (Langley et al. 2025) were included in these investigations. However, reliability of ${\dot{\mathrm{c}}}$La_max_ has already been assessed in a total of n = 11 articles focussing on *(hand-)cycling* (Adam et al. 2015; Harnish et al. 2023, 2024; Meixner et al. 2024a; Quittmann et al. 2021a, 2021b; Sablain et al. 2025), running (Quittmann et al. 2020, 2021b; Wawer et al. 2020), *rowing* (Held et al. 2023), *swimming* (Sengoku et al. 2024) and *isokinetic force tests* (Nitzsche et al. 2018a). According to previous guidelines, reliability in terms of intra-class correlation coefficient (ICC) are classified as *excellent* (≥ 0.90), *good* (0.75–0.90), *moderate* (0.50–0.75) and *poor* (< 0.50) (Koo & Li 2016).

Jennifer Adam et al. (2015) were the first to test the reliability of ${\dot{\mathrm{c}}}$La_max_ (and its components) in n = 23 amateur cyclists who performed 3 tests that were 3–6 days apart. The ${\dot{\mathrm{c}}}$La_max_ demonstrated *excellent* reliability (ICC = 0.904) which was still lower compared to the reproducibility of ${\dot{\mathrm{V}}\mathrm{O}}$_2max_ (ICC = 0.987) (Adam et al. 2015). Limits of agreement were ± 0.12 mmol/l/s. Interestingly, they demonstrated differences in reliability between ΔLa (ICC = 0.891), La_max_ (ICC = 0.856) and La_pre_ (ICC = 0.804). The latter demonstrated the by far highest variability (18.8%) of all ${\dot{\mathrm{c}}}$La_max_ components. Similar results were found in handcycling (ICC = 0.828) and cycling (ICC = 0.872) when two tests each were separated by 1 week in a group of n = 18 competitive triathletes (t_PCr_ = t_Pmax_) (Quittmann et al. 2021a). The corresponding limits of agreements for handcycling and cycling were ± 0.11 and ± 0.14 mmol/l/s, respectively.

These findings were replicated in an even larger group (n = 50) of experienced cyclists (Meixner et al. 2024a). Cyclists performed three trials (one as familiarisation) within 2 weeks that were at least 2 days apart. Depending on the method to define t_PCr_, the reliability of ${\dot{\mathrm{c}}}$La_max_ was found to be *excellent* for t_PCr_ = 3.5 s (ICC = 0.911) and *good* to *moderate* when time to attain peak power or the drop in power by 3.5% was used (ICCs = 0.866 and 0.794, respectively). In accordance with these findings, repeating 15-s all-out sprints on four consecutive days in n = 13 physically active males, reliability was observed to be *good* for ${\dot{\mathrm{c}}}$La_max_ (ICC = 0.80, CV = 7%), *moderate* for La_pre_ (ICC = 0.52, CV = 20%) and *poor* for the time when power dropped by 3.5% (ICC = 0.25, CV = 6%) (Sablain et al. 2025). Calculated power output at MLSS attained *excellent* reliability in amateur cyclists (ICC = 0.985, CV = 4%) and physically active males (ICC = 0.99, CV = 1.3%) (Adam et al. 2015; Sablain et al. 2025).

In contrast to these *promising* findings in cycling, colleagues from the United States of America demonstrated a substantially lower (*moderate*) reliability of ${\dot{\mathrm{c}}}$La_max_ (ICC = 0.66, variability = 18.6%) in n = 30 healthy participants (Harnish et al. 2023). They reported limits of agreement as high as ± *0.36* mmol/l/s. Similarily, these authors demonstrated poor to moderate reliability in a subsequent study when using t_Pmax_ (ICC = 0.47) or a fixed t_PCr_ = 5 s (ICC = 0.64) (Harnish et al. 2024). There are three reasons that probably explain the discrepancy between the studies. Firstly, Harnish and colleagues used a *portable* analyser that demonstrates an accuracy of approximately ± 0.4 mmol/l (Mentzoni et al. 2024). Secondly, they collected blood samples from the *fingertip* (instead of using the earlobe) which results in higher and less reliable measures of lactate concentration (Zhong et al. 2024). Lastly, blood samples for recording post-exercise lactate concentration were collected *every 2* min (after 1, 3, 5 and 7 min) until the first decline was observed (in contrast to every minute for 10 min) (Harnish et al. 2023). Since the time to reach peak post-exercise lactate concentration ranged from 1 to 9 min and tends to differ between this study (4.1 ± 1.5 min) and other exercise modalities like rowing (5.0 ± 0.3 min) and swimming (∼ 2 min, ranging from 1 to 10 min), we argue that a *higher* sample rate provides a *more robust* estimate of ${\dot{\mathrm{c}}}$La_max_ (Held et al. 2023; Mavroudi et al. 2023). However, the effect and reliability of sample rate has to be investigated in future studies. *In summary,* ${\dot{\mathrm{c}}}$La_max_ seems to by *highly reliable* in cycling as long as sample *site* (earlobe), blood *sampling* (every minute), and *analyser* (stationary) are chosen *appropriately*.

In running, ${\dot{\mathrm{c}}}$La_max_ attained *good* to *excellent* reliability when derived from a 100-m sprint test in competitive runners (n = 16) and triathletes (n = 18) (Quittmann et al. 2020, 2021b). Depending on the method used to derive t_PCr_, the initial reliability of tests that were 2 days apart was *good* (ICC = 0.868, time when power dropped by 3.5%) to *excellent* (ICC > 0.90 with t_inter_) (Quittmann et al. 2020). After familiarisation, ICCs further increased by 0.021–0.057. Since limits of agreement reduced from ± 0.15 to ± 0.10 mmol/l/s, we concluded that ${\dot{\mathrm{c}}}$La_max_ can be used *without* familiarisation (in recreational runners), but that it is *beneficial* to provide familiarisation—if possible. The high reliability of the 100-m sprint was replicated in a follow-up study when tests were 1 week apart (ICC = 0.868) (Quittmann et al. 2021b). In contrast to the *fixed-distance* approach, Corinna Wawer et al. (2020) conducted an extensive examination (n = 73 sport students) including several experiments of a *fixed-duration* approach in running. On the track, reliability was compared between an 8-s (ICC = 0.89), 10-s (ICC = 0.82), 12-s (ICC = 0.92) and 14-s all-out (ICC = 0.84) with only the 12-s sprint attaining *excellent* reproducibility (Wawer et al. 2020). Since a total of 3 sprints were performed *per day* for several days, participants might have reached their own tolerance for blood sampling. Whereas track testing attain *good* to *excellent* reliability, only *moderate* ICCs were found when using non-motorised treadmills in a 10-s and 12-s all-out (ICC = 0.76 and 0.79, respectively) (Wawer et al. 2020). Hence, field-based track sprinting is preferred over the use of non-motorised treadmills.

In rowing, ${\dot{\mathrm{c}}}$La_max_ derived from an 20-s all-out sprint attained *good* reliability (ICC = 0.85) in n = 17 competitive rowers that was considerably lower compared to mean power output (ICC = 0.98) when measurement were 1 week apart (Held et al. 2023). In this study, limits of agreement were ± 0.09 mmol/l/s. In swimming, colleagues from Japan demonstrated *excellent* reliability of ${\dot{\mathrm{c}}}$La_max_ derived from an 20-m all-out sprint (ICC = 0.913) within 4 days in n = 17 competitive swimmers (Sengoku et al. 2024). This was somehow surprising since blood samples were examined by using a *portable* analyser. Averaging the outcomes of two blood samples might at least in part) have coped to lower precision (± 0.4 mmol/l). Lastly, isokinetic (unilateral) leg extension exercise demonstrated *moderate* reliability when 8 or 16 maximal repetitions were performed at 210°/s in n = 32 trained participants (Nitzsche et al. 2018a). In both conditions, limits of agreement were ± 0.11 mmol/l/s which might be affected by the method to define t_PCr_ (time when power dropped by 3.5%).

*In conclusion,* ${\dot{\mathrm{c}}}$La_max_ demonstrates *good* to *excellent* reliability in various sports/modalities which represents a *necessity* for application in science and practice. However, examiners and practitioners have to ensure proper standardisation during the tests, that includes the *preparation, equipment, test characteristics and calculation approach.* Regarding the latter, *fixed* and *interpolated* approaches to define t_PCr_ seem to demonstrate *superior* reliability. According to previous studies, *averaging 2–3 blood samples that are collected in close temporal proximity* immediately before the start of the sprint is recommended to increase the reliability of La_pre_ (Poffé et al. 2024; Hauser et al. 2014; Adam et al. 2015).

## Specificity

The *specificity* of ${\dot{\mathrm{c}}}$La_max_ has been examined in several studies in terms of exercise *modality* (Nitzsche et al. 2018b; Wawer et al. 2020; Quittmann et al. 2021a, b, 2022b; Meixner et al. 2025a; Micke et al. 2025) and *sex* (Quittmann et al. 2022a; Held et al. 2023; Harnish et al. 2023; Thron et al. 2024b; Poffé et al. 2024; Meixner et al. 2024b, 2025a; Archacki et al. 2024; Micke et al. 2025). Differences observed in ${\dot{\mathrm{c}}}$La_max_ are likely affected by differences in (active) muscle mass, muscle perfusion, (passive) lactate distribution space and muscle fiber typology (Mader 2003; Nuzzo 2023). The findings of these studies highlight that ${\dot{\mathrm{c}}}$La_max_ is highly *specific* to the (amount of) involved muscle groups and/or movement as well as the individual’s sex. As such, practitioners who seek to quantify the ${\dot{\mathrm{c}}}$La_max_ are advised to apply *sport-specific* sprint tests. In sports that have not yet been investigated in terms of ${\dot{\mathrm{c}}}$La_max_, we encourage to develop new sport-specific (field-)tests to provide applicable measures. The following sub-chapters elaborate more on differences and correlations found between modalities as well as between (biological) females and males. However, the authors acknowledges that there are more than two (biological) sexes and several categories of gender identity, that are welcomed in terms of diversity but are beyond the scope of this review (Green et al. 2025).

### Exercise modality

As the first study to highlight the specificity of ${\dot{\mathrm{c}}}$La_max_, Nitzsche et al. (2018b) demonstrated that (ergometer) cycling results in a significantly *higher* ${\dot{\mathrm{c}}}$La_max_ (p < 0.001), when compared to (unilateral) isokinetic force tests (0.81 ± 0.09 vs. 0.28 ± 0.09 mmol/l/s, respectively) in n = 14 trained participants. Even more striking, ${\dot{\mathrm{c}}}$La_max_ was *not* significantly correlated between exercise modalities (r = 0.42, p > 0.05), which indicates that participants with a (relatively) high ${\dot{\mathrm{c}}}$La_max_ in cycling will *not* necessarily demonstrate a (relatively) high ${\dot{\mathrm{c}}}$La_max_ in force tests and vice versa (Nitzsche et al. 2018b). Similar findings were found when (conventional) cycling was compared to handcycling (Quittmann et al. 2021a) and running (Quittmann et al. 2021b) in a group of n = 18 competitive triathletes. While ${\dot{\mathrm{c}}}$La_max_ was found to be significantly *lower* in handcycling (d = –1.62, p ≤ 0.001) compared to cycling (0.32 ± 0.10 vs. 0.52 ± 0.14 mmol/l/s, respectively), running attained significantly *higher* (d = 0.709, p = 0.016) measures of ${\dot{\mathrm{c}}}$La_max_ compared to cycling (0.71 ± 0.16 vs. 0.60 ± 0.15 mmol/l/s, respectively). The ${\dot{\mathrm{c}}}$La_max_ observed in cycling did *not* (significantly) correlate to ${\dot{\mathrm{c}}}$La_max_ in handcycling (r = 0.455, p = 0.058) and running (r = 0.418, p = 0.084). We concluded that ${\dot{\mathrm{c}}}$La_max_ is *specific* in terms of the exercising *extremity* (upper vs. lower body) as well exercise *modality*. Hence, using ${\dot{\mathrm{c}}}$La_max_ derived from sprint tests in cycling to predict/simulate running performance seems to be problematic (Ji et al. 2021). This is of particular interest for *triathletes* who might demonstrate different metabolic profiles (in terms of ${\dot{\mathrm{V}}\mathrm{O}}$_2max_ and ${\dot{\mathrm{c}}}$La_max_) when tested in the three different disciplines.

Accordingly, in a triathlete with spinal cord injury, anecdotal evidence demonstrated that ${\dot{\mathrm{c}}}$La_max_ was found to be *highest* in 25-m all-out swimming, when compared to 15-s all-out handcycling and 110-m wheelchair racing (*lowest)* (Quittmann et al. 2022b). This indicates that even in wheelchair athletes who are restricted to (mainly) use their upper extremities, differences exist between exercise modalities. However, this finding has to be validated in a group of several (paralympic) athletes. Just recently, a subsample competitive of cyclists (n = 57) demonstrated a significantly higher ${\dot{\mathrm{c}}}$La_max_ when compared to competitive rowers (n = 95) (0.62 ± 0.12 vs. 0.30 ± 0.11 mmoll/l/s, respectively) (Micke et al. 2025). Similarly, cyclists attaining a high P_max_ (> 14.09 W/kg) demonstrated a significantly higher ${\dot{\mathrm{c}}}$La_max_ when compared to a lower power group (0.83 ± 0.10 vs. 0.75 ± 0.09 mmol/l/s, respectively) (Haase et al. 2025). These two studies indicate that specific/deliberate training adaptations result in different glycolytic abilities as can be observed in ${\dot{\mathrm{c}}}$La_max_. However, comparisons between subgroups are not valid as within-participants comparisons between exercise modalities.

Comparisons of ${\dot{\mathrm{c}}}$La_max_ within the same exercise modality between field and laboratory testing remain inconclusive. Whereas ${\dot{\mathrm{c}}}$La_max_ attained in 10-s all-out running was *similar* and *highly correlated* (ICC = 0.96) between track and non-motorised treadmill (0.74 ± 0.21 vs. 0.71 ± 0.20, respectively), kayaking on-water was found to result in a significantly *higher* ${\dot{\mathrm{c}}}$La_max_ when compared to kayak ergometry (0.51 ± 0.19 vs. 0.40 ± 0.16 mmol/l/s, respectively) that was *moderately* correlated between conditions (r = 0.68) (Wawer et al. 2020; Meixner et al. 2025a). It seems that standardizing test *duration* increases the transferability of ${\dot{\mathrm{c}}}$La_max_—at least in running.

### Sex

The data of two German *diploma theses* were the first to indicate differences in ${\dot{\mathrm{c}}}$La_max_ between female (n = 10) and male (n = 19) cyclists (0.50 ± 0.15 vs. 0.59 mmol/l/s, respectively) (Poffé et al. 2024). Similar results were found in a group of N = 50 (n = 20 females) experienced cyclists even though another method to define t_PCr_ was used in this study (0.47 ± 0.09 vs. 0.58 ± 0.16 mmol/l/s, respectively). Since the average difference was fairly comparable (∼ 0.10 mmol/l/s), the comparison between females and males did *not* attain statistical significance (0.62 ± 0.15 vs. 0.71 ± 0.26 mmol/l/s, respectively) in another investigation (Harnish et al. 2023). This is likely due to the higher standard deviation that was probably caused by fingertip blood sampling every 2 min and/or using a portable analyser (Zhong et al. 2024; Mentzoni et al. 2024). Similarly, differences between females and males were found to be similar in endurance (0.38 ± 0.14 vs. 0.45 ± 0.07 mmol/l/s, respectively) and speed-power athletes (0.52 ± 0.08 vs. 0.58 ± 0.09, respectively) (Archacki et al. 2024). The authors concluded that *“if the ongoing long-term adaptation exists, the sex differences […] tend to decrease.”*

In 100-m all-out running, females demonstrated a significantly *lower* ${\dot{\mathrm{c}}}$La_max_ when compared to males (Quittmann et al. 2022a; Thron et al. 2024b). These comparisons might be affected by test duration that was (on average) ∼ 2 s higher in females which might result in lower values on ${\dot{\mathrm{c}}}$La_max_ (Wawer et al. 2020; Langley et al. 2024; Porter & Langley 2025). Whereas an average difference of ∼ 0.10 mmol/l/s (d = − 1.34, p < 0.001) was observed in competitive (long-distance) runners (0.65 ± 0.13 vs. 0.74 ± 0.14 mmol/l/s, respectively) (Quittmann et al. 2022a), a considerably *higher* difference was observed in *adolescent* sprinters and middle-distance runners (0.73 ± 0.13 vs. 0.88 ± 0.19 mmol/l/s, respectively) (Thron et al. 2024b). This discrepancy might be related to the even higher reliance on glycolysis in shorter events making sex differences more evident (Hargreaves & Spriet 2020). Another reason could be that sex differences in body structure, physiology and function usually increase dramatically after the onset of puberty due to hormonal (testosterone) perturbations (Joyner et al. 2025).

In sports with dominant upper-body involvement, the trend for females demonstrating lower values of ${\dot{\mathrm{c}}}$La_max_ was even *more* pronounced (Held et al. 2023; Meixner et al. 2025a). Competitive rowers demonstrated an average difference of 0.12 mmol/l/s when performing a 20-s all-out test on the ergometer (0.23 ± 0.06 vs. 0.35 ± 0.10 mmol/l/s, respectively). Accordingly, differences between females and males were more pronounced in a subsamples of n = 95 rowers (0.25 ± 0.05 vs. 0.36 ± 0.12 mmol/l/s, respectively) when compared to a subsample of n = 57 competitive cyclists (0.59 ± 0.14 vs. 0.63 ± 0.11 mmol/l/s, respectively) (Micke et al. 2025). To date, the highest difference was observed in on-water kayaking of national U21 canoe polo players with females demonstrating a ${\dot{\mathrm{c}}}$La_max_ that was only half compared to their male counterparts (0.35 ± 0.04 vs. 0.71 ± 0.13 mmol/l/s, respectively). Given the high glycolytic demands of canoe polo (Zwingmann et al. 2020), it serves as another argument that *sex differences increase with higher glycolytic energy contribution*. Additionally, previous studies found that *males* have a higher adaptability of upper-body strength and power development (Lu & Duan 2024).

*In summary,* various studies in different sports demonstrate that *females* (on average) demonstrate a significantly *lower* ${\dot{\mathrm{c}}}$La_max_ compared to males which might be due to differences in body/muscle mass, muscle fiber typology and/or haematology (Nuzzo 2023; Joyner et al. 2025; Milic et al. 2010). It seems that differences between females and males are higher in (a) *shorter* events and (b) *upper-body* sports that seems to rely more on glycolysis and lactate metabolism (Hargreaves & Spriet 2020; Lovell et al. 2013). Future studies need to *expand* this comparison and match for certain influential characteristics (e. g. ${\dot{\mathrm{V}}\mathrm{O}}$_2max_, performance or fat free mass).

## Relationships to performance and physiology

Given the *diverse nature* of exercise performance and physiology, this chapter summarises the assessed relationships in 5 sub-chapters focussing on *sprint performance* (e. g. maximal power output), *time trial performance* (≥ 1 min), *physiological parameters* (e. g. ${\dot{\mathrm{V}}\mathrm{O}}$_2max_ or lactate threshold), *simulation approaches* (calculated vs. measured MLSS) and *oxygen (de-)saturation* (via near-infrared spectroscopy). By doing so, readers (hopefully) access the information of interest more straightforward.

Given that ATP resynthesis is the fundamental goal of all kinds of energy supply, there is a physiological reasoning behind correlating lactate concentrations/accumulation and exercise performance. Assuming a certain amount of energy per mM lactate, it seems reasonable to expect a relationship to glycolytic-demanding tasks. Especially in terms of sprint performance, significant correlations can be explained by the higher glycolytic demands necessary to achieve a certain power. However, all of the following correlation analyses do not necessarily imply causation. This is particularly important for durations > 1 min and simulation approaches, that seem to result in rather inconclusive findings.

### Sprint performance

Since ${\dot{\mathrm{c}}}$La_max_ testing requires some type of all-out sprint that can be quantified externally by means of *power, time and/or distance*, previous studies have extensively assessed their relationship to ${\dot{\mathrm{c}}}$La_max_ and mostly reported *strong correlations to sprint performance*. Since sprint performance is typically mediated by the amount of (fat-free) body mass, relative power output demonstrates a higher associated with ${\dot{\mathrm{c}}}$La_max_. Even though studies vary in terms of exercise modality, test characteristics, calculations and the participants’ performance level, various studies highlight that $\dot{c}$*La*_*max*_* is associated with parameters of sprinting*—especially in terms of mean power output or (analogously) the amount of work performed during the all-out test. Relationships to peak or maximal power outputs seem to depend on the cohort with more specifically trained participants demonstrating *weaker* relationships. This might be due to deliberately training *phosphagenous* metabolism that has an ATP turnover (power) > 4 times higher compared to glycolysis (Gastin 2001).

In cycling, the work performed per kilogram fat-free mass during a 15-s all-out test explains *∼ 85%* of the variance in ΔLa (Meixner et al. 2024b). Accordingly, an increase in post-exercise lactate concentration of 1 mmol/l equivalents to ∼ 12 J/kg (fat-free mass) more work, which was found to be *similar* in females and males (Meixner et al. 2024b). Accordingly, normalising peak/mean power to body weight demonstrated (slightly) *higher* correlations with ${\dot{\mathrm{c}}}$La_max_ in (n = 11) (inter-)nationally competitive cyclists (Clark & Macdermid 2025). While *absolute* (r = 0.75, p = 0.007) and *relative* peak power output (r = 0.80, p = 0.003) already correlated *strongly* with ${\dot{\mathrm{c}}}$La_max_, *mean* power output was even *higher* associated with ${\dot{\mathrm{c}}}$La_max_ (r = 0.83, p = 0.002 and r = 0.88, p < 0.001, respectively) (Clark & Macdermid 2025). Depending on the method to estimate t_PCr_, mean power correlated significantly with ${\dot{\mathrm{c}}}$La_max_ (r = 0.43–0.48, p = 0.008–0.017) while using t_Pmax_ demonstrated the *strongest* relationship (Yang et al. 2023).

With respect to all-out sprints between 10 and 30 s, ${\dot{\mathrm{c}}}$La_max_ and mean power output were *strongly* associated for *longer* durations (Langley et al. 2024). In the 10-s all-out, mean *absolute* (r = 0.141, p = 0.631) and *relative* power output (r = 0.423, p = 0.120) did *not* correlate significantly with ${\dot{\mathrm{c}}}$La_max_ (Langley et al. 2024). Similar findings were observed in the 15-s all-out (r = − 0.021, p = 0.931 and r = 0.309, p = 0.265, respectively). In the 30-s all-out, mean relative power output correlated significantly with ${\dot{\mathrm{c}}}$La_peak_ (r = 0.727, p = 0.003), whereas mean absolute power did *not* (r = − 0.051, p = 0.840). Peak power output (absolute as well as relative) was *not* found to significantly correlate with ${\dot{\mathrm{c}}}$La_max_ in this study (Langley et al. 2024). In contrast, (absolute) *peak* power output significantly correlated with ${\dot{\mathrm{c}}}$La_max_ when using t_Pmax_ (r = 0.710, p = 0.001) or t_P3.5%_ (r = 0.719, p = 0.001) in a mixed-sex group of (n = 18) competitive triathletes (Quittmann et al. 2021a, b). La_Δ_ exhibited significant correlations with 10-s all-out relative mean (r = 0.70, p < 0.001) and peak power (r = 0.65, p < 0.01) in n = 22 trained male cyclists (Haase et al. 2025). In handcycling, (absolute) peak power was significantly correlated with ${\dot{\mathrm{c}}}$La_max_ in (n = 12) male (r = 0.604, p = 0.037) and (n = 18) mixed-sex (r = 0.729, p = 0.001) able-bodied triathletes (Quittmann et al. 2018, 2021a). However, future studies need to validate these finding in (inter-)nationally competitive handcyclists.

In running, ${\dot{\mathrm{c}}}$La_max_ significantly correlated with 100-m all-out sprint time (r = − 0.812, p < 0.001) as well as maximal velocity (r = 0.815, p < 0.001) and power (r = 0.735, p < 0.01) (Quittmann et al. 2020). Since these relationships might be *overestimated* by applying a fixed-distance, correlations were also assessed for ΔLa which demonstrated similar yet lower correlations (r = − 0.629, p < 0.01, r = 0.060, p < 0.01 and r = 0.581, p < 0.05, respectively) that explained around *40%* of the respective variance (Quittmann et al. 2020). These relationships for maximal power (r = 0.719, p < 0.001) could be replicated during a follow-up study in (n = 18) competitive triathletes (Quittmann et al. 2021b). Thron et al. (2024b) found a significantly positive relationship between ${\dot{\mathrm{c}}}$La_max_ and maximal sprinting speed (r = 0.74, p < 0.01) in (n = 34) adolescent/young sprinters and middle-distance runners.

In swimming, ${\dot{\mathrm{c}}}$La_max_ significantly correlated with 50-m front crawl time (24.6 ± 0.7 s, r = − 0.546, p < 0.05) in n = 17 competitive (male) swimmers (Sengoku et al. 2024). Furthermore, correlations between ${\dot{\mathrm{c}}}$La_max_ and 15-m split times *decreased* from 0 to 15 (r = − 0.627, p < 0.01) to 30–45 m (r = − 0.465, p > 0.05). However, ${\dot{\mathrm{c}}}$La_max_ did *not* correlate with estimated maximal velocity (r = 0.224, p > 0.05) when derived from semi-tethered swimming (Sengoku et al. 2024). When determining ${\dot{\mathrm{c}}}$La_max_ (or probably ${\dot{\mathrm{c}}}$La_peak_) from 25-, 35- and 50-m sprints, correlations between the corresponding (average) speed were observed to be significantly positive in every trial (r = 0.541, p = 0.046; r = 0.587, p = 0.027 and r = 0.839, p < 0.001, respectively) with *stronger* relationships found in *longer* distances (Mavroudi et al. 2023). However, the results of this study might be affected by using a *portable* analyser and *fingertip* blood sampling (Mentzoni et al. 2024; Zhong et al. 2024). In adolescent female swimmers, significantly positive correlations (r ≥ 0.44) were observed between ${\dot{\mathrm{c}}}$La_peak_ (20-s all-out) and (average) 50- and 100-m velocity (Keller & Wahl 2025).

In kayaking, 15-s all-out peak and mean power output demonstrated *moderate* correlations (r ≈ 0.60 and 0.70, respectively) with ${\dot{\mathrm{c}}}$La_max_ in (n = 8) elite canoe polo payers (Zwingmann et al. 2020). Similarly, significant correlations between ${\dot{\mathrm{c}}}$La_max_ and on-water sprinting velocity over 40 and 50 m (r = 0.72) as well as normalised mean power in 15-s all-out ergometer testing were demonstrated in (n = 15) U21 players (Meixner et al. 2025a). In rowing, mean power in 20-s all-out ergometry was significantly related to ${\dot{\mathrm{c}}}$La_peak_ (r = 0.81, p < 0.001) in (n = 17) competitive rowers (Held et al. 2023). La_max_ also demonstrated a highly significant positive correlation with mean power output (r = 0.74, p < 0.001). In isokinetic force testing, a significant correlation between ${\dot{\mathrm{c}}}$La_max_ and maximal power output was observed (r = 0.716, p = 0.02) (Nitzsche et al. 2020). In a following 6-week resistance exercise intervention, the increase in maximal power output was significantly correlated with the difference in ${\dot{\mathrm{c}}}$La_max_ (r = 0.502, p = 0.012). This is a *very intriguing finding* as it points towards a *causal relationship between an increased *$\dot{c}$*La*_*max*_* and improvements in sprint performance.* However, this finding must be replicated/validated by future research and over an extended time period.

### Time trial performance

Several studies examined the relationship between ${\dot{\mathrm{c}}}$La_max_ and sport-specific (time trial) performance in *running* (Quittmann et al. 2022a; Thron et al. 2024b), *cycling* (Clark and Macdermid 2025), *alpine ski mountaineering (Skimo)* (Wagner et al. 2024), *kayaking* (Zwingmann et al. 2020) and *rowing* (Schünemann et al. 2023). In running, augmenting the traditional Joyner model (Joyner 1991) of ${\dot{\mathrm{V}}\mathrm{O}}$_2max_, %${\dot{\mathrm{V}}\mathrm{O}}$_2max_ and running economy by ${\dot{\mathrm{c}}}$La_max_ led to a significant (yet minor) improvement in 5000-m time trial performance (R^2^ + 4.4%) in a mixed-sex group of N = 44 trained runner/triathletes (Quittmann et al. 2022a). This indicated that—when controlled for the other parameters of the Joyner model—participants with *higher* ${\dot{\mathrm{c}}}$La_max_ demonstrated *better* 5000-m time trial performance (shorter time). However, performing step-wise linear regression exclusively for males (n = 29), ${\dot{\mathrm{c}}}$La_max_ was *not* included. Hence, the significant augmentation seems to be *affected by sex* (Chapter 5.2). Interestingly, the relative increase in velocity over the last 200 m (‘finishing kick’) demonstrated a significantly positive correlation with ${\dot{\mathrm{c}}}$La_max_ (r = 0.389, p < 0.010) (Quittmann et al. 2022a). In 400-m sprinters (n = 11), Maximiliane Thron et al. (2024b) demonstrated a significant correlation between ${\dot{\mathrm{c}}}$La_max_ and World Athletic scores (r = 0.68, p < 0.05).

In cycling, ${\dot{\mathrm{c}}}$La_max_ did *not* significantly correlate with absolute (r = 0.52, p = 0.098) and relative (r = 0.29, p = 0.393) maximal 1-min power output in n = 11 (inter-)national endurance cyclists (Clark & Macdermid 2025). However, combining ${\dot{\mathrm{c}}}$La_max_ and ${\dot{\mathrm{V}}\mathrm{O}}$_2max_ in multiple regression explained R^2^ = 52.4% of the variance in 1-min power (Clark & Macdermid 2025). It has to be mentioned that the measurements of this study might be affected by the use of a *portable* lactate analyser (Mentzoni et al. 2024). Skimo performance (lasting 3:35 ± 46 s) was *not* significantly correlated with ${\dot{\mathrm{c}}}$La_max_ (r = 0.22, p > 0.05), even though this correlation appeared to (slightly) increase in the finals (r = 0.32, p < 0.05) (Wagner et al. 2024). Given the high *specificity* of ${\dot{\mathrm{c}}}$La_max_, these relationships might be affected by using an 80-m sprint test in running.

In kayaking, 7- and 15-min time trial performance demonstrated *moderate* to *weak* relationships (r ≈ 0.40) with ${\dot{\mathrm{c}}}$La_max_ (Zwingmann et al. 2020). In rowing, 2000-m time trial performance correlated significantly with ${\dot{\mathrm{c}}}$La_max_ (r = − 0.67, p = 0.083) indicating that a *higher* ${\dot{\mathrm{c}}}$La_max_ allows for *lower* times (*better* performance) (Schünemann et al. 2023). In terms of *pacing*, power output over the first 30 m was significantly associated with ${\dot{\mathrm{c}}}$La_max_ (r = 0.65, p = 0.049) whereas the final 300-m power output was *not* (r = 0.44, p = 0.204). The authors concluded that *“future studies must show under which conditions (e. g. *$\dot{V}O$_*2peak*_*) an increase or decrease in [*$\dot{c}$*La*_*max*_*] may increase rowing-specific performance”*(Schünemann et al. 2023). This seems to be relevant as ‘anaerobic’ energy contribution in 2000-m rowing competitions is assumed to be ∼ 12–33% (Treff et al. 2021) whereas a lower contribution of 11.5 ± 2.8% was observed in this study (Schünemann et al. 2023).

*In summary,* linear relationships between ${\dot{\mathrm{c}}}$La_max_ and efforts lasting ≥ 1 min are way *less strong* when compared to associations with sprit performances. This might be due to the fact that *oxidative* energy contribution substantially increases in these type of activities (Gastin 2001; Barclay 2017; Hargreaves & Spriet 2020). Another reason could be that these relationships are (in fact) *non-linear*, which is why the assumption of *linearity* causes false-negative results. Since some athletes with a *high* ${\dot{\mathrm{c}}}$La_max_ perform *well* in endurance events, whereas others do not, it seems that *singular correlations might be misleading*. Instead, ${\dot{\mathrm{c}}}$La_max_ should be integrated in a *multifarious* metabolic profile and account for other important (physiological) characteristics. Hence, a method worth exploring in future research is the application of *directed acyclic graphs* (a tool of *‘causal interference’*) since other (physiological) parameters might act as *confounders, colliders or mediators* (Williamson et al. 2014; Peng et al. 2024).

### Physiological parameters

In swimming, ${\dot{\mathrm{c}}}$La_max_ correlated significantly *positive* with dryland upper- and lower-body strength parameters (r ≈ − 0.60, p < 0.01), significantly *negative* with lactate threshold (r ≈ − 0.55, p < 0.01) and was *not* (significantly) associated with ${\dot{\mathrm{V}}\mathrm{O}}$_2peak_ (r ≈ 0.15, p > 0.05) in n = 24 national-level female athletes (Keller & Wahl 2025). However, the close associations to strength parameters might be *overestimated* by allowing foot contact to the pool wall during ${\dot{\mathrm{c}}}$La_max_ testing (Keller & Wahl 2025). However, similar findings in swimming demonstrated *high* correlations (r > 0.80, p < 0.01) between ${\dot{\mathrm{c}}}$La_max_ and parameters of load-velocity profiling (Sengoku et al. 2024). In cycling, there was *no* significantly positive correlation between absolute ${\dot{\mathrm{V}}\mathrm{O}}$_2peak_ and ${\dot{\mathrm{c}}}$La_max_ in a group of N = 21 (n = 8 females) sports students (r = 0.40, p = 0.07) (Pohl et al. 2024) An opposing trend indicating a *negative* (yet *moderate*) relationship between ${\dot{\mathrm{V}}\mathrm{O}}$_2max_ and ${\dot{\mathrm{c}}}$La_max_ was found in physically active males (r = − 0.19, p = 0.527) (Sablain et al. 2025) and adolescent/young sprinters and middle-distance runners (Thron et al. 2024b). In running, the fractional utilization of ${\dot{\mathrm{V}}\mathrm{O}}$_2max_ at the onset of blood lactate accumulation (%${\dot{\mathrm{V}}\mathrm{O}}$_2max_) correlated *moderately* with ${\dot{\mathrm{c}}}$La_max_ (r = − 0.439, p = 0.003) in N = 44 (n = 15 females) trained runners/triathletes (Quittmann et al. 2022a). In handcycling, ${\dot{\mathrm{c}}}$La_max_ correlated significantly *negative* with the maximal power output attained in a graded exercise test (r = − 0.646, p = 0.023), whereas the power output corresponding to 4 mmol/l lactate concentration was *not* significantly associated with ${\dot{\mathrm{c}}}$La_max_ (r = − 0.415, p > 0.05) in a group of n = 12 able-bodied triathletes (Quittmann et al. 2018). The ${\dot{\mathrm{c}}}$La_max_ did *not* significantly correlate with lactate threshold (r = − 0.42, p = 0.149) and calculated MLSS (r = − 0.16, p = 0.593) in cycling (Sablain et al. 2025). Similarily, ${\dot{\mathrm{c}}}$La_max_ did not improve multiple regressions to predict lactate threshold in n = 83 cyclists and triathletes (Fischer et al. 2025). In running, anaerobic speed reserve, representing the difference between maximal sprinting speed and maximal aerobic velocity, was significantly correlated with ${\dot{\mathrm{c}}}$La_max_ in a group of n = 44 trained endurance runners/triathletes (r = 0.644, p < 0.001) and n = 34 adolescent sprinters and middle-distance runners (r = 0.74, p < 0.01) even though different calculations of this parameter were applied (Quittmann et al. 2022a; Thron et al. 2024b). Similar relationships were observed for the speed reserve ratio (r = 0.56, p < 0.01) which reflects the ratio between maximal sprinting speed and maximal aerobic speed (Thron et al. 2024b).

*In summary,* aside from significant relationships to strength parameters, ${\dot{\mathrm{c}}}$La_max_ demonstrates (at highest) *moderate* relationships to other physiological parameters. It seems that ${\dot{\mathrm{c}}}$La_max_ represents a physiological characteristic of its own and might be a *promising augmentation* to the toolset of exercise testing. However, the *moderate* relationships might be due to the fact that *matching* for certain parameters (e. g. ${\dot{\mathrm{V}}\mathrm{O}}$_2max_ or %${\dot{\mathrm{V}}\mathrm{O}}$_2max_) is challenging in (well-)trained athletes who generally demonstrate highly *individual* profiles. Future studies need to (re-)assess these relationships by applying standardised procedures in a larger and probably more homogeneous cohort. Aside from that, ${\dot{\mathrm{c}}}$La_max_ should be *validated* by assessing its correlation to *glycolytic enzyme activity and/or muscle fiber typology*. Just recently, type II muscle fiber percentage demonstrated—out of several exercise tests—the *strongest* association with lactate concentration 3 min after performing a 30-s all-out sprint test (r = 0.67, p < 0.001) (Van de Casteele 2025). This seems *promising* for future studies that should replicate this study including ${\dot{\mathrm{c}}}$La_max_, which might result in even *higher* correlations. Furthermore, the empirical relationships between ${\dot{\mathrm{c}}}$La_max_ and other physiological parameters are less obvious, as indicated in earlier simulation approaches (Mader & Heck 1986).

### Simulation approaches

Simulation approaches to estimate MLSS in cycling by means of ${\dot{\mathrm{V}}\mathrm{O}}$_2max_ and ${\dot{\mathrm{c}}}$La_max_ have been applied in several studies (Poffé et al. 2024; Hauser et al. 2014; Wahl et al. 2017; Ji et al. 2021; Sablain et al. 2025). Deviations to MLSS or surrogate measures in cycling are usually *quite wide on the individual level* and were found to average at + 5 W (range from − 16 to + 25 W) (Poffé et al. 2024), + 12 ± 20 W (Hauser et al. 2014), + 1 ± 14 W (Wahl et al. 2017) and − 5 ± 17 W) (Sablain et al. 2025). The latter study did *not* determine MLSS by several constant load trials and used lactate threshold and ${\dot{\mathrm{V}}\mathrm{O}}$_2peak_ from an incremental test (80 + 40 W every 3 min) which might explain their *underestimation* (Sablain et al. 2025). It has to be mentioned that these studies still differ with respect to the applied experimental procedures as well as the used formulas and constants.

In contrast to the studies that *transparently* reported the used formulas and constants, Poffé et al. (2024) only references to the *“default values”* of the software used (version 2.0, INCYD GmbH, Salenstein Switzerland). Aside from *insufficient* reporting in the underlying diploma theses, the article of Poffé et al. (2024) does *not* comply with proper scientific standards in terms of *transparency* and *independency*, which is why conclusions derived from this article should be interpreted with *great caution*. Hauser et al. (2014) observed that calculated and experimentally measured MLSS highly correlate (r = 0.92, p < 0.001) but demonstrate *very high limits of agreement* (− 20 to + 50 W). Similar findings (r = 0.96, − 30 to + 20 W) were found in a group of N = 19 (n = 4 females) triathletes/cyclists (Wahl et al. 2017). In this study, calculated MLSS was rounded to 10 W in order to match the accuracy of experimentally derived MLSS which affects the examination of correlation and deviation.

Movement velocity or cycling cadence has recently been highlighted in a well-crafted invited review on endurance performance prediction (Dunst et al. 2025). The main takeaway of this work is that velocity/cadence affects movement efficiency (oxygen demand per Watt) which is one of the major input parameters of Mader’s simulation approach. Neglecting this aspect may lead to variations in metabolic kinetics and MLSS by up to 100 W (Dunst et al. 2025). Hence, future studies should be aware of these relationships and consider the dynamic changes in oxidative and glycolytic metabolism across different velocities/cadences when examining Mader’s simulation approach in cycling.

In running, Ji et al. (2021) simulated MLSS by using ${\dot{\mathrm{V}}\mathrm{O}}$_2peak_ from incremental running and ${\dot{\mathrm{c}}}$La_peak_ from all-out cycling to compare/correlate it with running performance and other measures of lactate threshold in n = 10 sub-elite middle- and long- distance runners. Calculated MLSS indicated *moderate* to *good* agreement with other threshold concepts like the velocity corresponding to a lactate concentration of 4 mmol/l (ICC = 0.74) and modified maximal deviation (ICC = 0.87) that demonstrated *similar* correlations (r = 0.61–0.76) with running performance (Ji et al. 2021). However, the calculations in this study are *flawed* in three ways since (1) a graded exercise test was used for measuring oxygen uptake (Sperlich et al. 2015), (2) a 30-s all-out was used to determine ${\dot{\mathrm{c}}}$La_peak_ (Porter & Langley 2025; Langley et al. 2024) and (3) a sprint test in cycling was used to predict running performance (Quittmann et al. 2021b). Hence, future studies need to feed running-based simulation approaches with *proper* and *sport-specific* experimental data to compare calculated MLSS with actual running performance. *In summary,* studies that applied simulation approaches in cycling and running do *not* yet provide sufficient evidence for recommending their (and especially the corresponding software) in order to predict MLSS in a concrete/individual athlete.

### Oxygen (de-)saturation

Given that lactate metabolism seems to represent a link between *glycolytic* and *oxidative* energy supply (Brooks et al. 2021), 3 studies examined the relationship between ${\dot{\mathrm{c}}}$La_max_ and muscle oxygen (de-)saturation of M. vastus lateralis as measured by near-infrared spectroscopy (Dunst et al. 2023b; Reinpõld et al. 2024; Porter & Langley 2025). During a 60-s all-out test, Anna Katharina Dunst et al. (2023b) demonstrated a significant correlation between the time constant of oxygen desaturation and ${\dot{\mathrm{c}}}$La_max_ (r = 0.768, p < 0.017) and t_PCr_ (r = 0.822, p < 0.001) in n = 9 elite (male) track cyclists. A *higher* time constant implies that it takes *longer* for the muscle to desaturate. The authors highlighted that a *high* ${\dot{\mathrm{c}}}$La_max_ represents a *higher* rate of phosphocreatine replenishment via *glycolysis* which results in a *reduced* need for oxidative (re-)phosphorylation and consequently *lower* desaturation rate (Dunst et al. 2023b).

During a 30-s all-out sprint N = 32 male cyclists (n = 16 juniors and seniors, respectively), ${\dot{\mathrm{c}}}$La_peak_ correlated significantly with the mean response time (r = − 0.44, p < 0.05) and time delay (r = − 0.39, p < 0.05) but *not* time constant (r = − 0.25, p > 0.05) of muscle oxygen desaturation (Reinpõld et al. 2024). Compared to the findings of Dunst et al. (2023b), *slower* oxygen desaturation kinetics were observed that might be due to their (endurance) training background and *lower* lactic power (0.42 ± 0.09 vs. 0.95 ± 0.18 mmol/l/s, respectively). However, given that Reinpõld et al. (2024) assessed ${\dot{\mathrm{c}}}$La_peak_ in a 30-s all-out and used a *portable* lactate analyser (Diaglobal DP110 Lactate Photometer Plus), the results of this study should be interpreted with *caution*.

The relationship between ${\dot{\mathrm{c}}}$La_max_ and muscle oxygen (de-)saturation was assessed across various sprint durations of 10, 15 and 30 s in a group of n = 13 male trained cyclists (Porter & Langley 2025). The time spent desaturated differed significantly between sprint durations and demonstrated a significant inverse relationship with ${\dot{\mathrm{c}}}$La_max_ (r = − 0.673, p < 0.001). However, the amplitude of muscle oxygen desaturation did *not* correlate with ${\dot{\mathrm{c}}}$La_max_ across test durations. A very strong *negative* correlation (r = − 0.994, p < 0.001) was observed between the modelled kinetics of lactate and muscle oxygen (de-)saturation (Porter & Langley 2025). On average, the (modelled) time to attain ${\dot{\mathrm{c}}}$La_max_ (8.92 ± 0.77 s) aligned with the nadir of oxygen desaturation (8.47 ± 1.50 s), but were *not* significantly correlated (r = 0.508, p = 0.11). Porter and Langley (2025) hypothesised that *“monitoring the individuals’ [muscle oxygen] desaturation profile during all-out sprint cycle ergometry may help to identify the optimal test duration to determine”* ${\dot{\mathrm{c}}}$La_max_.

*In summary,* muscle oxygen (de-)saturation (kinetics) of M. vastus lateralis seem to be related to ${\dot{\mathrm{c}}}$La_max_ in all-out sprints in cycling lasting 10–60 s. Future studies need to replicate these findings and expand the test spectrum by *longer test durations* (e. g. 3-min all-out), *muscles* (e. g. M. vastus medialis or M. gastrocnemius) and *various exercise modalities* (e. g. running or rowing) in order to gain more insights in the interaction between ${\dot{\mathrm{c}}}$La_max_ glycolytic energy contribution and oxygen (de-)saturation in *multi-faceted* exercise scenarios.

## Adaptability

Adaptations/alterations of ${\dot{\mathrm{c}}}$La_max_ over time have been reported in (only) *five* articles (Sperlich et al. 2010; Manunzio et al. 2016; Hommel et al. 2019; Nitzsche et al. 2020; Quittmann et al. 2022b) while a previous review only included two of these studies due to their specific criteria (Langley et al. 2025). Sperlich et al. (2010) performed a randomised crossover intervention in n = 26 competitive (youth) swimmers who performed 2 × 5 weeks of low-intensity training (LIT) at high volume or HIIT, respectively. Since post-exercise lactate concentrations were collected after a 100-m time trial (t_test_ = 86 ± 10 s), this measure is referred to as ${\dot{\mathrm{c}}}$La_peak_. Whereas 5 weeks of HIIT *increased* ${\dot{\mathrm{c}}}$La_peak_ significantly (d = 0.43, p < 0.01) by + 20%, a significant *decline* (d = − 0.51, p < 0.01) of –30% was observed after 5 weeks of LIT (Sperlich et al. 2010). Hence, HIIT might be an adequate stimulus for glycolytic pathways to increase enzyme activities and other processes that result in a *higher* glycolytic/lactic power.

In contrast, Jennifer Hommel (formerly Adam) et al. (2019) demonstrated a significant *reduction* of ${\dot{\mathrm{c}}}$La_max_ by − 0.08 ± 0.05 mmol/l/s after only *two* weeks of sprit interval training (SIT). The surprising reduction of ${\dot{\mathrm{c}}}$La_max_ following SIT was discussed to result from the considerably high lactate (and simultaneous H^+^) concentrations during the sessions that might have initiated an elevated decomposition of lactate during the mitochondria (Hommel et al. 2019). However, there is an *urgent need* for future research to assess the effect of HIIT and SIT on ${\dot{\mathrm{c}}}$La_max_ and cellular processes in terms of enzyme and transporter activities. During the whole intervention, the training groups performed specific sessions 3 times per week for 6 weeks (Hommel et al. 2019). While the SIT group performed 4–6 × 30-s sprints followed by 4 ½ min at 30 W, the LIT group performed 1 h at a lactate concentration of 1.5–2.5 mmol/l that was controlled for every 10 min (Hommel et al. 2019). Even though 1.5–2.5 mmol/l seems to *comply* with previous notations of the recently popular *‘Zone 2’* (Seiler 2010), it may be argued that this is quite a *wide* range for LIT and might *not* align with Fat_max_ (Achten & Jeukendrup 2004; Alkhatib 2022). Despite this argument, ${\dot{\mathrm{c}}}$La_max_ did *not* change in the LIT group, which was discussed to be due to the *short* (and probably familiar) exposure to this regime.

A considerably *longer* exposure of 6 months was observed in a team of 4–5 team riders that finished 2nd in the race across America (Manunzio et al. 2016). Cyclists followed a *pyramidal* training intensity distribution (63 ± 16%/28 ± 13%/9 ± 4%) at an average volume of about 8 h/w. Within the preparation period, ${\dot{\mathrm{c}}}$La_max_ decreased by 16.3 ± 8.1% (p = 0.03). At closer, visual inspection, a slight *increase* after the general preparation (mostly Zone 1) was followed by a more pronounced *decrease* after specific preparation (more Zone 2/3). It would have been interesting to assess the relationship between the individuals’ training intensity distribution and alterations in ${\dot{\mathrm{c}}}$La_max_ at several points in time to gain preliminary insights on the adaptability of ${\dot{\mathrm{c}}}$La_max_ in elite athletes.

The development of ${\dot{\mathrm{c}}}$La_max_ over the course of *six years* has been documented in an internationally-competitive triathlete with spinal cord injury, that competes in *swimming*, *handcycling* and *wheelchair racing* (Quittmann et al. 2022b). Increasing training volume from 414 to 604 h/year and shifting intensity distribution towards more LIT (from 77-17-6 to 88-8-4%) coincided with a *decrease* in ${\dot{\mathrm{c}}}$La_max_ from 0.56 to 0.36 mmol/l/s withintwo years. More interestingly, alterations in ${\dot{\mathrm{c}}}$La_max_ were displayed in conjunction with changes in lactate threshold (4 mmol/l) and maximal/peak oxygen uptake (Quittmann et al. 2022b). In certain periods, alterations of ${\dot{\mathrm{c}}}$La_max_, lactate threshold and oxygen uptake seemed to *align* with the hypothesised model (Appendix 1). For example, a drop in lactate threshold (− 6 W) was observed despite a rather constant oxygen uptake (+ 0.4 ml/min/kg). In the same time, ${\dot{\mathrm{c}}}$La_max_ increased from 0.42 to 0.61 mmol/l/s (Quittmann et al. 2022b). On another occasion, lactate threshold increased from 141 to 146 W, despite a decrease in peak oxygen uptake (− 1.7 ml/min/kg) that might be influenced by a substantial decrease in ${\dot{\mathrm{c}}}$La_max_ (− 0.18 mmol/l/s) (Quittmann et al. 2022b). Even though these are rather anecdotal examples in a single athlete, future studies could build up on this and assess the interplay between these three measures in certain training periods.

Resistance exercise seems to *increase* ${\dot{\mathrm{c}}}$La_max_ significantly (d = 0.974, p = 0.032) if performed 3 times a week for 6 weeks (Nitzsche et al. 2020). Nitzsche et al. (2020) examined the effect of two types of resistance training in N = 24 strength-trained males with three lower-extremity exercises (leg *press*, leg *extension* and leg *flexor*) and 5 sets each. The *low-load* (high-volume) group performed 5 sets at 50% of their 1-repetition maximum up to muscle failure, whereas the *high-load* (low-volume) group performed 5 × 10 repetitions at 70%. Even though the average increase was *higher* in the high-load group (+ 0.42 vs. + 0.27 mmol/l/s), a significant *increase* in ${\dot{\mathrm{c}}}$La_max_ was only found in the low-load group (d = 0.384, p = 0.233 vs. d = 0.406, p = 0.022). This in contrary to previous research hypothesizing that high-load resistance training *“may induce preferential growth of type II muscle fibers”* (Grgic & Schoenfeld 2018) that have a *higher* levels of glycolytic enzymes (Schiaffino & Reggiani 2011). Aside from a mere increase in Type II fiber size, skeletal muscle hypertrophy seems to *interact* with (glycolytic) energy metabolism (Baumert et al. 2024) that explains its effect on ${\dot{\mathrm{c}}}$La_max_. Hence, resistance exercise seems to *increase* ${\dot{\mathrm{c}}}$La_max_ even in a short period of time. Future studies need to *differentiate* the effects of different modes (in terms of *intensity, repetitions and failure*) and periodization models (*linear, undulating and blocked*) on ${\dot{\mathrm{c}}}$La_max_.

*In summary,* the (fairly sparse) body of research indicates that (high volumes of) LIT tends to *decrease* ${\dot{\mathrm{c}}}$La_max_, whereas resistance exercise seems to *increase* this parameter. Aside from the need to replicate these findings, there is still *uncertainty* on how ${\dot{\mathrm{c}}}$La_max_ is affected by different types of HIIT and SIT. Future studies need to carefully design properly matched interventions to examine their effect. It is recommended to study the whole spectrum of HIIT covering (traditional) long intervals, short intervals, repeated sprint training and SIT (Buchheit and Laursen 2013).

## Future directions

Even though there has been a *growing* body of knowledge on ${\dot{\mathrm{c}}}$La_max_ with articles currently published on a *monthly* basis, there are still a lot of *open* questions that future research can address in the upcoming years. Out of the total number of participants that have been examined in the included articles (N = 1125), the proportion of *females* is *25%* (n = 285). Hence, the is an *urgent* need to assess glycolytic metabolism by means of ${\dot{\mathrm{c}}}$La_max_ in females—especially in *trained* athletes. Shining examples can be seen in competitive *swimming*, as one study examined sexes *equally* (Sperlich et al. 2010), while another study focused *exclusively* on females (Keller & Wahl 2025). Furthermore, studies aiming to compare ${\dot{\mathrm{c}}}$La_max_ between females and males should *expand* the existing literature and consider to *match* for certain influential characteristics (e. g. ${\dot{\mathrm{V}}\mathrm{O}}$_2max_, performance and/or fat free mass). However, ${\dot{\mathrm{c}}}$La_max_ still needs to be *validated* by means of *glycolytic enzyme activity and/or muscle fiber typology* to check if current assumption on its relevance in exercise physiology hold true.

The *adaptability* of ${\dot{\mathrm{c}}}$La_max_ is still sparsely explored (Langley et al. 2025). Hence, colleagues in exercise science are *encouraged* to augment the fairly time-efficient ${\dot{\mathrm{c}}}$La_max_ testing in the pre- and post-measurements of their training interventions in several sports. Different types of HIIT like (traditional) long intervals, short intervals, repeated sprint training and SIT are of particular interest for ${\dot{\mathrm{c}}}$La_max_ adaptation (Buchheit & Laursen 2013). However, studies need to carefully design properly *matched* interventions to examine their effect and take the participants prior training history into consideration. This might be performed as a high-intensity block training intervention (Mølmen et al. 2019). During training interventions, the *interplay* between ${\dot{\mathrm{c}}}$La_max_ and other physiological parameters measures (e. g. ${\dot{\mathrm{V}}\mathrm{O}}$_2max_ and/or %${\dot{\mathrm{V}}\mathrm{O}}$_2max_) should be assessed. In terms of resistance and concurrent training, the effect of different modes (in terms of *intensity, repetitions and failure*) and periodization models (*linear, undulating and blocked*) on ${\dot{\mathrm{c}}}$La_max_ should be explored. It is assumed that the *increasing effect of resistance training and decreasing effect of LIT* might result in an almost unchanged ${\dot{\mathrm{c}}}$La_max_ in *concurrent* training interventions. Potentially, the augmentation of ${\dot{\mathrm{c}}}$La_max_ can help to individualise training prescription in constant and intermittent endurance sports (Wackerhage et al. 2025).

Since relationships between ${\dot{\mathrm{c}}}$La_max_ and parameters of performance (≥ 1 min) and physiology are still *inconclusive*, a method worth exploring in future research is the application of *directed acyclic graphs* (a tool of ‘causal interference’) since other (physiological) parameters might act as *confounders, colliders or mediators* (Williamson et al. 2014; Peng et al. 2024). Furthermore, these relationships should be (re-)assessed in a *larger* and probably more *homogeneous* cohort of (highly) *trained* athletes which may highlight the need for *collaborative multi-center studies*. The same accounts to *simulation approaches* in cycling and running that require *proper* and *sport-specific* experimental data to compare calculated MLSS with actual performance in various sports and should take movement velocity/frequency into consideration (Dunst et al. 2025).

In terms of oxygen (de-)saturation, research needs to expand the test spectrum by *longer test durations* (e. g. 3-min all-out), *other muscles* (e. g. M. vastus medialis or M. gastrocnemius) and *various exercise modalities* (e. g. running or rowing) in order to gain more insights in the *interaction* between ${\dot{\mathrm{c}}}$La_max_ glycolytic energy contribution and oxygen (de-)saturation in multi-faceted exercise scenarios. It might be interesting to examine ${\dot{\mathrm{c}}}$La_max_ in the—recently en vogue—field of *‘durability’* (Jones 2023). Since glycolytic Type II fibers are typically more prone to fatigue, it seems likely that ${\dot{\mathrm{c}}}$La_max_ *correlates negatively* with ‘*physiological resilience’*. However, this assumption needs to be *verified*.

Even the *procedure*s to determine ${\dot{\mathrm{c}}}$La_max_ have some open research questions to offer. Future studies may assess the effect of *different training regimes* within the days preceding the test, several *ergogenic aids* like caffein, bicarbonate and nitrate, different *force transmissions* (cleated shoes vs. straps), different *warm-up regimes* and different *initial resistances* and *pedalling frequencies*. Furthermore, research may explore the *time to reach La*_*max*_ in various sports and athletes. Empirical research should *consider to refrain from t*_*PCr*_ and simply divide ΔLa by t_test_, as phosphagenous contribution can hardly be approximated.

## Recommendations

Aside from *future directions* for research to be conducted on ${\dot{\mathrm{c}}}$La_max_, this section summarises the *recommendations for exercise testing and training* that were derived from the included N = 60 Journal articles. It is *evident* that ${\dot{\mathrm{c}}}$La_max_ has gained increased scientific attention *and started to spread across the globe*. In terms of *terminology*, a differentiation between the experimentally-derived *maximal lactate accumulation rate* (${\dot{\mathrm{c}}}$La_max_) and the theoretical *maximal rate of glycolysis (dLa/dt*_*max*_*)* is recommended, while the latter may also be called *glycolytic/lactic power*. Analogous to the distinction between *maximal* and *peak* oxygen uptake, we could use the term *peak* lactate accumulation rate (${\dot{\mathrm{c}}}$La_peak_) if there is reason to believe that the test procedure was *not* appropriate to determine ${\dot{\mathrm{c}}}$La_max_. The time equivalent to account for energy resynthesis from phosphocreatine (t_PCr_) is also recommended for science and practice (instead of the formally t_alac_).

The ${\dot{\mathrm{c}}}$La_max_ demonstrates *good* to *excellent* reliability in various sports/modalities which represents a *necessity* for application in science and practice. However, examiners and practitioners have to *ensure proper standardisation* during the tests, that includes the *preparation, equipment, test characteristics and calculation approach.* In the days preceding ${\dot{\mathrm{c}}}$La_max_ testing, athletes should follow their *normal* (or slightly *carbohydrate-rich*) diet and *avoid* the use of *ergogenic supplements* (e. g. creatine monohydrate). Also, acute carbohydrate intake within ∼ 30 min preceding the test should be *avoided* due its highly individual response. Ergometers that allow *isokinetic* mode are *preferred* over ergometers with linear resistances as these are more likely to attain the targeted cadences (≥ 130 rpm). To ensure *immediate* force transmission on the pedals, the use of *cycling-specific cleated* shoes is recommended over the mere use of straps. *Stationary* rather than *portable* lactate analyser should be used in conjunction with ${\dot{\mathrm{c}}}$La_max_ tests, as the latter typically demonstrate a precision of ± 0.4 mmol/l. However, averaging the values of two portable analysers may (to a certain extent) cope with this inaccuracy.

For warm-up, we recommend 10 min of low-intensity (e. g. 1–2 W/kg in cycling) that are either interspersed by a several short (< 5–10 s) accelerations (up to ∼ 5 times the basic load) or followed by 2–4 starts (e. g. 10–15 m in running). However, since body weight merely correlates with performance (especially in cycling), an individualised intensity (e. g. 50% of lactate threshold or critical power) could be applied. After the warm-up, *passive* rest should be applied between 5 and 10 min that should be extended (by active or passive rest) until La_pre_ ≤ 1.5 mmol/l. To increase reliability, La_pre_ should be determined as the average of 2–3 blood samples that are collected in close temporal proximity immediately before performing the sprint test.

Test duration (or distance) should be rather short to *avoid an inflated oxidative contribution and/or pH-dependent PFK suppression*. Recent findings indicate that ${\dot{\mathrm{c}}}$La_max_ should be derived from all-out sprint tests lasting *10–12 s* by using a *fixed-duration or -distance approach*. Performing sprint tests ≥ 15 s is *not* recommended and should be accompanied by the label ${\dot{\mathrm{c}}}$La_peak_. Depending on the respective exercise *modality*, this offers a *variety* of possibilities. In order to improve standardisation between participants and investigations, *fixed-time approaches are preferred over fixed-distance approaches,* even though the latter might be of particular interest in sports that do *not* rely on power measurements. In *isokinetic* cycling, studies should select a *pedalling frequency* ≥ *130* rpm and *report* the initial resistance and the start of the test/measurement.

Appropriate blood sampling is *crucial* for ${\dot{\mathrm{c}}}$La_max_ as it is mainly derived from post-exercise lactate measurements. Blood samples should be collected *at the earlobe and not on the fingertip,* as the latter is more sensitive, prone to sweat contamination and seems result in higher and less reliable measures of lactate concentration. However, fingertip blood sampling might be applied if athletes need to test *themselves*. Since the time to reach La_max_ was found to range from 1 to 9 min, a sampling rate of (at least) *one blood sample per minute* until 10 min is recommended. In highly *glycolytic* athletes (who might demonstrate an extended time to reach La_max_), researchers may continue blood sampling until a decline in lactate concentration is observed. For modelling post-exercise lactate kinetics, sampling duration might be extended to 15 (or even 30) min.

In terms of ${\dot{\mathrm{c}}}$La_max_ calculation, the method used to define t_PCr_ matters with respect to its *reliability, applicability and interpretability.* As an *individual* method, *t*_*Pmax*_* should be preferred over t*_*P3.5%*_ as it demonstrates *superior* reliability, *stronger* correlations to peak power and is *not* related to the (inaccurate) measurement error of a certain ergometer. *Fixed/interpolated* methods for t_PCr_ (t_inter_) typically result in more reliable outcomes of ${\dot{\mathrm{c}}}$La_max_ and are recommended in exercise modalities that do *not* rely on power measures. Depending on the test mode (fixed-duration vs. -distance) t_inter_ can be calculated according to Eq. 2.

Aside from *sprint performance* and *strength parameters* that seem to be strongly associated with ${\dot{\mathrm{c}}}$La_max_, its relationships to other measures of performance and/or physiology are fairly *inconclusive*. Studies that applied simulation approaches in cycling and running do *not* yet provide sufficient evidence for recommending their use in order to predict MLSS in a concrete/individual athlete. The few training interventions on the *adaptability of* ${\dot{\mathrm{c}}}$La_max_ indicate that (high volume of) LIT tends to *decrease* ${\dot{\mathrm{c}}}$La_max_, whereas resistance exercise seems to *increase* this parameter with contradictory findings in HIIT and SIT.

## Supplementary Information

Below is the link to the electronic supplementary material.

Supplementary file1 Apendix 1 Simplified framework of how maximal rate of glycolysis (blue) and maximal oxygen uptake (red) interact. Based on the theoretical concept to explain the metabolic origin of ‘anaerobic threshold’ (Mader & Heck 1986). The dashed line (grey) represents the simulated ‘lack of lactate (pyruvate) formation, that is covered by fatty acid formation’ (lactate disappearance > ‘gross’ lactate formation). It was assumed that higher maximal rates of glycolysis result in lower outcomes of maximal fat oxidation rate (MFO and Fat_max_, orange) and maximal lactate steady state (MLSS, purple), as long as maximal oxygen uptake (${\dot{\mathrm{V}}\mathrm{O}}$_2max_) is the same. This would (theoretically) result in a leftward shift in Fat_max_ and MLSS (JPG 271 KB)

Supplementary file2 Appendix 2 Terminology and descriptions among peer-reviewed articles in English language. Articles are listed in chronological order based on their acceptance. Most important quotations describing maximum rate of glycolysis and/or maximal lactate accumulation rate are also listed. Abbr. = Abbreviation; ATP = Adenosine triphosphate; MLSS = maximal lactate steady-state (DOCX 43 KB)

Supplementary file3 Appendix 3 Warm-Up specifications in the experimental studies (quotations) (DOCX 35 KB)

## Abbreviations

ATP: Adenosine triphosphate
${\dot{\mathrm{c}}}$La_max_: Maximal lactate accumulation rate (empirical settings/whole-body level)
${\dot{\mathrm{c}}}$La_peak_: Peak lactate accumulation rate (when procedures do not allow for a maximal rate, ≥ 15 s)
CV: Coefficient of variability
d: Cohen’s d (effect size)
dLa/dt_max_: Maximal rate of glycolysis (simulation-based settings/muscle level)
Fat_max_: Intensity corresponding to maximal fat oxidation rate
HIIT: High-intensity interval training
La_max_: Maximal post-exercise lactate concentration
La_pre_: Lactate concentration immediately before the start (average of 2–3 samples, ≤ 1.5 mmol l^−^^1^)
LIT: Low-intensity training (mostly at high-volume)
ΔLa: Difference between maximal post-exercise lactate concentration and pre-exercise values
ICC: Intra-class correlation coefficient
MFO: Maximal fat oxidation rate
MLSS: Maximal lactate steady-state
PFK: Phosphofructokinase
SIT: Sprint interval training
t_Ff_: Time span up to the first systematic deviation from fatigue-free force–velocity profile
t_inter_: Interpolated phosphagenous time
t_lac_: Time equivalent to account energy resynthesis derived from lactate accumulation (glycolysis)
t_P3.5%_: Time when power output decreased by 3.5%
t_PCr_: Time equivalent to account for energy resynthesis from phosphocreatine (phosphagenous time)
t_Pmax_: Time to attain peak power output
t_test_: Duration of the all-out sprint test (should be ∼ 10–12 s)
${\dot{\mathrm{V}}\mathrm{O}}$_2max_: Maximal oxygen uptake
${\dot{\mathrm{V}}\mathrm{O}}$_2peak_: Peak oxygen uptake (when procedures do not allow for a maximal rate)
%${\dot{\mathrm{V}}\mathrm{O}}$_2max_: Fractional utilization of maximal oxygen uptake at lactate threshold
