# Supplementary figures and images for: Maximal lactate accumulation rate (c˙Lamax): Current evidence and future directions for exercise testing and training
Source: Eur J Appl Physiol. 2025 Oct 31;126(1):1–36. doi: 10.1007/s00421-025-06022-7 (PMC12881007; doi:10.1007/s00421-025-06022-7)

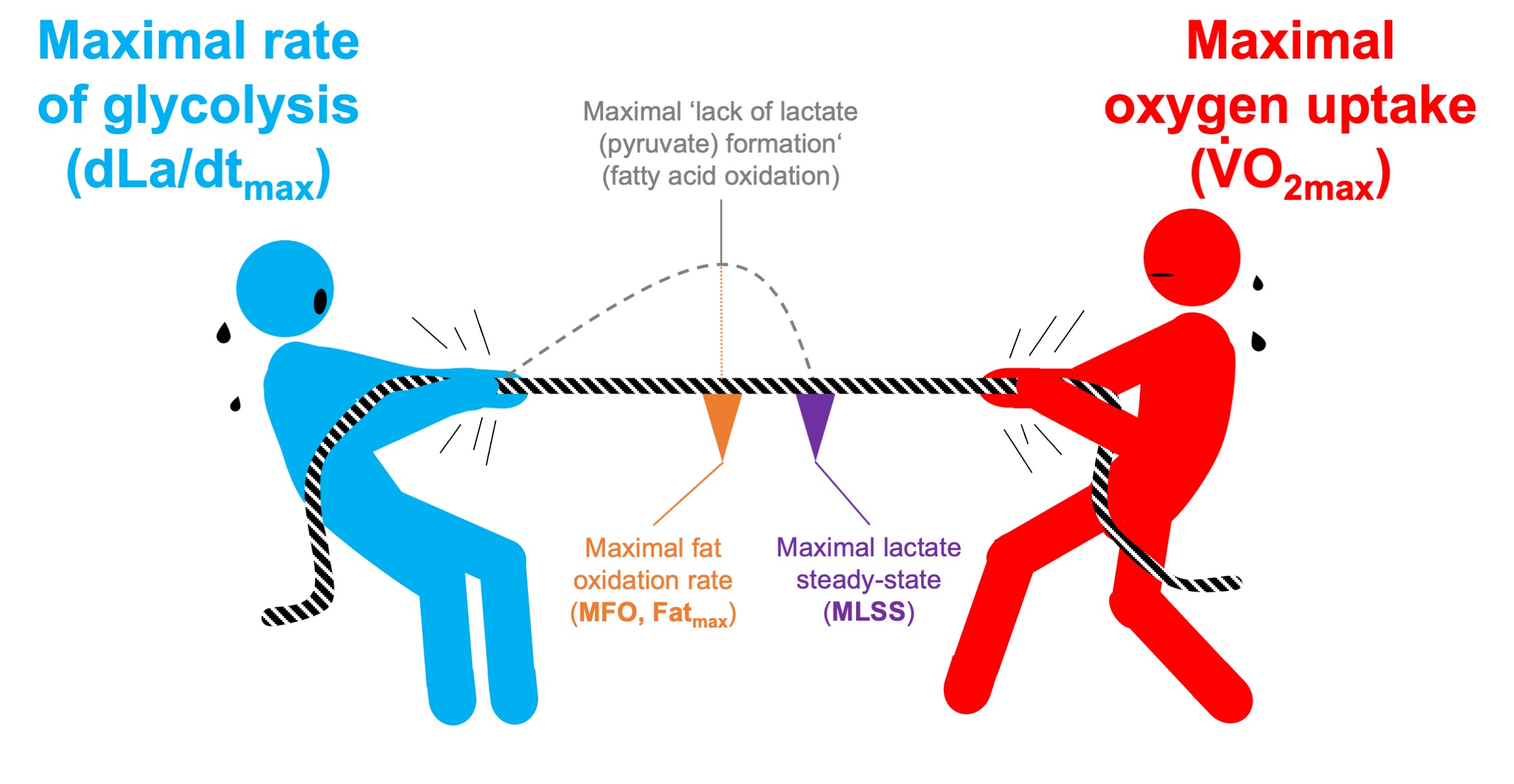

Supplement: Supplementary file 1 — Supplementary file1 Apendix 1 Simplified framework of how maximal rate of glycolysis (blue) and maximal oxygen uptake (red) interact. Based on the theoretical concept to explain the metabolic origin of ‘anaerobic threshold’ (Mader & Heck 1986). The dashed line (grey) represents the simulated ‘lack of lactate (pyruvate) formation, that is covered by fatty acid formation’ (lactate disappearance > ‘gross’ lactate formation). It was assumed that higher maximal rates of glycolysis result in lower outcomes of maximal fat oxidation rate (MFO and Fatmax, orange) and maximal lactate steady state (MLSS, purple), as long as maximal oxygen uptake (${\dot{\mathrm{V}}\mathrm{O}}$V˙O2max) is the same. This would (theoretically) result in a leftward shift in Fatmax and MLSS (JPG 271 KB) [file 421_2025_6022_MOESM1_ESM.jpg]
