# Supplementary material for: Maximal lactate accumulation rate (c˙Lamax): Current evidence and future directions for exercise testing and training
Source: Eur J Appl Physiol. 2025 Oct 31;126(1):1–36. doi: 10.1007/s00421-025-06022-7 (PMC12881007; doi:10.1007/s00421-025-06022-7)
Supplement: Supplementary file 3 — Supplementary file3 Appendix 3 Warm-Up specifications in the experimental studies (quotations) (DOCX 35 KB) [file 421_2025_6022_MOESM3_ESM.docx]

Supplementary Material for the article *“Maximal lactate accumulation rate (ċLa_max_): Current evidence and future directions for exercise testing and training”* by Quittmann OJ (*Eur J Appl Physiol)*

**Appendix – Warm-Up specifications in the experimental studies (quotations)**

**(Hand-)Cycling**

Grassi et al. (1995):

*(not specified)*

Poffé et al. (2024):

*“The subjects performed a* ***12 min*** *warm-up on the bicycle ergometer* ***at 1.5 W·kg^−1^*** *[study of Kleinschmidt (2004)]* ***or 2 W·kg^−1^*** *[study of Weber (2003)]. This workload corresponded to the easy/moderate intensity domain. The subjects were free to use their preferred cadence to minimize peripheral fatigue (21). After the warm-up, the subjects* ***rested for 10 min*** *by sitting on a chair.”*

Hauser et al. (2014):

*“In order to detect V̇La_max_ the subjects performed a sprint-test lasting 15 s which consisted of a* ***12 min*** *warm-up period with a constant load set* ***at 1.5 times*** *of the individual* ***body weight****, followed by a* ***second exercise bout*** *with a constant load* ***of*** ***50 W for ten minutes****.”*

Adam et al. (2015):

*“Subjects were sitting on the cycle ergometer and had a* ***warming up of 12 minutes*** *pedaling at a power of* ***1.5 fold of body weight****.* ***In the middle*** *of that time* ***a short sprint attempt of five seconds*** *was interposed. The warm-up was* ***followed by 10 min cycling at 50 W****.”*

Manunzio et al. (2016):

*“After an initial warm-up at* ***2 W·kg^−1^ for 10 min****, followed by* ***5 min of passive rest****, a 15 s all-out sprint test was performed […].”*

Wahl et al (2017):

*(not specified)*

Nitzsche et al. (2018a):

*“Local warm-up of the leg muscles (****moderate stretching****) was performed prior to each test.”*

Quittmann et al. (2018):

*“To familiarise the participants with handcycling propulsion and sport-specific load, an* ***incremental familiarisation protocol with decreasing stage durations*** *was performed at their first visit to the laboratory (see Fig. 1a). […] Afterwards, participants performed an active* ***recovery of 5 min at 20 W****. […] The active recovery was* ***followed by 5 min of passive recovery*** *before performing the 15-s All-Out sprint test.”*

Hommel et al. (2019):

*“Participants started with a warm-up session consisting of* ***12 min*** *pedalling* ***at a power of 1.5 W∙kg^-1^*** *body mass on an electronically braked cycle ergometer […].* ***In the middle*** *of this period,* ***a short sprint attempt of 5 s*** *was interposed. It was followed by* ***10 min*** *cycling,* ***at 50 W****, to mitigate the increased lactate concentration.”*

Quittmann et al. (2021a):

*“The participants performed a standardised low-intensity warm-up of* ***ten minutes*** *including* ***three acceleration bursts*** *(Ozkaya, 2013). The basic load of the warm-up for [handcycling] and [cycling] was* ***30 and 100 W****, respectively (Coso & Mora-Rodríguez, 2006; Weber, Chia, & Inbar, 2006). The acceleration bursts were applied for* ***ten seconds*** *each and power output was increased* ***up to five times the basic load*** *(****150 W in [handcycling]*** *and* ***500 W in [cycling]****) (see Appendix 2). After the warm-up, the participants* ***rested for five minutes*** *in a sitting position.”*

Ji et al. (2021):

*“The participants first performed a* ***10 min*** *standardized warm-up* ***at 1.5 W kg^−1^*** *body mass. After an additional* ***passive rest for 5 min****, a 30 s sprint test was performed […].”*

Dunst et al. (2023a,b):

*“The participants warmed up before each sprint with* ***6 minutes*** *of low-intensity cycling (****1–1.5 W·kg^−1^*** *body weight),* ***followed by a 3-s maximal sprint****. Participants* ***rested passively for 10 minutes*** *between warm-up and testing.”*

Yang et al. (2023):

*“An initial warm-up was performed for* ***10 min at 2 W·kg^−1^****. They further* ***rested passively*** *in the sitting position on the ergometer was* ***for 5 min*** *(Figure 1).”*

Harnish & Miller (2023):

*“Participants completed three repeated sprint sessions consisting of a standard* ***10-min easy*** *warm-up* ***at ~100 W****.”*

Harnish et al. (2023):

*“Each session consisted of a standard* ***10 min self-chosen easy*** *(RPE 3 on 10 pt scale) warm up of* ***~30–100 W****. Following the warm up, the subjects were asked to complete a* ***1 min rest*** *period.”*

Haase et al. (2024):

*“Participants performed a* ***10-minute*** *warm-up session prior to each sprint test. The warm-up consisted of cycling at a workload of* ***0.5*** *Watts per kilogram (****W/kg****) while maintaining a cadence range of* ***60 to 80 rpm****. Following the warm-up, a* ***short break of at least one minute*** *was provided to the participants.”*

Langley et al. (2024):

*“Prior to the V̇La_max_ test participants conducted a warm-up consisting of cycling for* ***12-min at*** *a normalised power output of* ***1.5 W.kg^−1^*** *on a […] cycle ergometer […], a* ***6 s sprint*** *was conducted at a normalised power output of* ***7.5 W.kg^−1^ after 6-min*** *of the warm-up (Tomaras and MacIntosh 2011). Following the warm-up, participants completed an* ***active recovery*** *cycle at a standardised power output of* ***50 W for 10-min*** *to aid lactate clearance.”*

Meixner et al. (2024a,b):

*“For all three visits, all cyclists warmed up for* ***10 min*** *cycling* ***at 1.5 W/kg*** *body mass and* ***resting for 3 min*** *[…].”*

Archaki et al. (2024):

*“The test began with a* ***5-min warm-up****, wherein participants pedaled at their own pace with resistance loads of* ***25 and 50 W****, interspersed with* ***brief sprints lasting up to 5 s****.”*

Reinpõld et al. (2024):

*“At least a 20 min active recovery period [following an incremental test] was included between the experimental phases, consisting of a* ***10 min ride at 100 W*** *and* ***at least a 10 min*** *ride at* ***80%*** *of the power output at the first ventilatory threshold (****VT1****). When capillary blood lactate level was lowered to* ***less than two mmol/L****, then the 30 s sprint cycling test was conducted.”*

Harnish et al. (2024):

*“Prior to each sprint, subjects completed an easy 10 min warm-up as previously described (Harnish et al., 2023).”*

Porter & Langley (2025):

*“Participants subsequently undertook a warm‐up comprising of* ***12‐min*** *cycling at a power output of* ***1.5 W·kg^−1^*** *on a static cycle ergometer […]. A* ***5‐s sprint*** *was conducted at a power output of* ***7.5 W·kg^−1^ after*** *the initial* ***6 min*** *to prime the physiological systems for the subsequent maximal cycle test (Tomaras & MacIntosh, 2011). Following 12‐min warm‐up, participants performed an* ***active recovery****, cycling at* ***50 W for 10 min*** *to aid a reduction of hyperlactataemia.”*

Fischer et al. (2025):

*“Following a* ***10-min warm-up at 2 W⋅kg^–1^****, the sprint test was performed […].”*

Sablain et al. (2025):

*“Each sprint test was preceded by a* ***10-min*** *warm-up, with a resistance set* ***at 1.5 × body mass****, during which three* ***short (<3 s) accelerations*** *were performed* ***at minutes 4, 5 and 6****.”*

Haase et al. (2025):

*“Prior to the sprint test, the participants performed a* ***10-min*** *warm-up, cycling at a workload corresponding to* ***0.5 Watt per kilogram bodyweight*** *while maintaining a pedaling frequency of* ***60–80 rpm****.”*

Clark & Macdermid (2025):

*“A standardized warm-up was performed including* ***12 minutes*** *of cycling at a power output corresponding to* ***1.5 times body weight*** *(kg), followed by a* ***5 s of all out isokinetic sprint*** *at 130 rpm and then* ***10 min of cycling at 50 W*** *(Adam et al., 2015; Hauser et al., 2014).”*

Meixner et al. (2025a):

*“During all 4 visits, cyclists warmed up for* ***10 min*** *by cycling* ***at 1.5 W/kg*** *BM, followed by a* ***3-min rest*** *[…].”*

Micke et al. (2025):

*“Each session began with a* ***10-minute*** *warm-up at* ***low intensity*** *of* ***less then 2 mmol/L*** *blood lactate concentration. […] In all cases, lactate levels were ensured to be* ***below 1.5 mmol/L****. If the threshold was exceeded, the* ***warm-up was extended****, and the sprint was delayed* ***until*** *the* ***resting lactate dropped*** *below this value.”*

**Running**

Quittmann et al. (2020):

*“As a standardised warm-up, every session started with* ***five minutes of low-intensity jogging*** *at a self-paced velocity. Afterwards, the participants performed* ***five minutes of running drills*** *with five exercises, including ankle drills, skipping, high knee running, high knee bounce skips and B skips. Every exercise was performed* ***twice for 10 to 15 m starting every 30 s****. At the end of the warm-up, the participants performed* ***three almost maximal starts for 10–15 m*** *starting* ***every 90 s****. Immediately before the 100-m sprint, the participants* ***rested for another five minutes*** *in a sitting position.”*

Wawer et al. (2020):

*“Before sprint running, subjects conducted a standardized warm-up:* ***ten minutes moderate*** *running (****70% at 4 mmol ·L^-1^ T****), an* ***individual stretching-program****, and* ***three increased speed*** *ending at an intensity of* ***80-90% of sprint running****. […] To minimize effects of warm-up on maximal lactate level after exercise, the resting level had to be low* ***(≤ 2.0 mmol·L^-1^****).*

Quittmann et al. (2021b):

*“In running, the participants performed a 100-m sprint test on an indoor track, including a* ***standardized warm-up of 15 min as recently published*** *[Quittmann et al. 2020].”*

Quittmann et al. (2022b):

“The participants performed the 100-m all-out sprint test and the **standardised warm-up of 15 minutes** **including technical drills and starts** **as described previously** *[Quittmann et al. 2020, 2021b]*.”

Thron et al. (2024):

*“After a* ***standardized warm-up****, each athlete conducted two 100-m sprints […]. Before the first sprint, the subjects* ***rested for 10 minutes*** *and 20 μL of capillary blood was collected from the right earlobe immediately before the sprint to assess resting lactate (La_pre_).”*

Wagner et al. (2024):

*“As part of a standardized warm-up, participants engaged in* ***5 min of low-intensity jogging*** *at a self-paced velocity. Following the warm-up, participants performed* ***two almost maximal starts for 5–10 m****, starting* ***every 90 s.*** *Immediately before the 80 m sprint, participants* ***rested*** ***for*** *an additional* ***5 min*** *in a sitting position.”*

Pohl et al. (2024):

*“Then, the [Running Sprint testing (RST)] started with a* ***standardized 10-min warmup*** *that included* ***mobilization****,* ***activation*** *of the muscles of the lower limbs* ***and acceleration runs*** *to prepare for a following maximal sprint. The specific conditions of all warmup exercises are dis-*

*played in Table 2. After performing each exercise for one time (Table 2, column Exercise), the participants returned the covered distance (Table 2, column Distance) to the start with a low intense run and continued immediately with the next exercise. Each exercise was demonstrated by the investigator. Intensities (Table 2, column Intensity) were instructed by the investigator and applied individually by the participants. After the warmup, participants followed a* ***time of walking and sitting until*** *the desired La_rest_ of the particular condition (at baseline and lactate+) was reached. In the baseline RST, participants attained La_rest_ of* ***≤1.5 mmol·L^−1^****, in lactate+ La_rest_ of ≥2.5 mmol·L^−1^ was attained. The participants only remained seated during the lactate+ RST procedure immediately after the warmup and did not have a predetermined walking time as in the other RSTs. In CHO−, CHO+ and acuteCHO RST, the participants followed the same walking and sitting procedure as in baseline.”*

**Swimming**

Sperlich et al. (2010):

*(not specified)*

Teixeira et al. (2023):

*“[…] the participants had an* ***individual*** *warm-up of* ***15-20 min*** *of* ***self-paced intensity*** *following* ***5 min of passive rest****.”*

Mavroudi et al. (2023):

*“After a* ***light 10 min warm-up*** *at self-selected pace, each athlete completed 3 swimming sprints […].”*

Sengoku et al. (2024):

*“Participants were instructed to conduct a self-selected* ***warm-up mimicking their competition routine****. This was followed by* ***5 min of passive rest*** *while seated. The duration of the rest period was in accordance with previous research [Quittmann et al. 2021a,b; Held et al. 2024].”*

Keller & Wahl (2025):

*“After an* ***individualized warm-up*** ***on land and in water (~ 15 min)****, athletes first performed the 20 s sprint test […].”*

**Rowing**

Schünemann et al. (2023):

*“In case, the resting lactate values were higher than 2.0 mmol·L^−1^, participants were advised to* ***continue rowing with a low intensity until*** *blood lactate levels were* ***below 2.0 mmol·L^−1^****. The intensity of the warm-up program was kept low (****100 W****) to avoid effects on maximum post-exercise lactate.”*

Held et al. (2023):

*“Participants completed a standardized low-intensity warm-up of* ***10 minutes at*** *a low intensity/ heart rate (corresponding to a blood lactate concentration of* ***2 mmol/L****) before the 20-second all-out tests, followed by* ***five minutes of passive rest*** *while seated.”*

Micke et al. (2025):

*“Each session began with a* ***10-minute*** *warm-up at* ***low intensity*** *of* ***less then 2 mmol/L*** *blood lactate concentration. […] In all cases, lactate levels were ensured to be* ***below 1.5 mmol/L****. If the threshold was exceeded, the* ***warm-up was extended****, and the sprint was delayed* ***until*** *the* ***resting lactate dropped*** *below this value.”*

**Kayaking**

Zwingmann et al. (2020):

*“Before each [time trial], athletes warmed up for* ***10 min at*** *an individual but low intensity (≤****70% of maximal heart rate*** ***or*** ***rate of perceived exertion*** *[1-10]****≤4****).”*

Meixner et al. (2025b):

*“The athletes conducted a* ***5 min*** *warm-up on the ergometer* ***at a low speed (6 – 8 km/h)*** *aiming to not induce a* ***raise in*** *capillary blood* ***lactate****.”*

**Isokinetic force tests**

Nitzsche et al. (2018a):

*“Local warm-up of the leg muscles (****moderate stretching****) was performed prior to each test.”*

Nitzsche et al. (2018b):

*(not specified)*

Nitzsche et al. (2020):

*(not specified)*
