# Supplementary material for: Maximal lactate accumulation rate (c˙Lamax): Current evidence and future directions for exercise testing and training
Source: Eur J Appl Physiol. 2025 Oct 31;126(1):1–36. doi: 10.1007/s00421-025-06022-7 (PMC12881007; doi:10.1007/s00421-025-06022-7)
Supplement: Supplementary file 2 — Supplementary file2 Appendix 2 Terminology and descriptions among peer-reviewed articles in English language. Articles are listed in chronological order based on their acceptance. Most important quotations describing maximum rate of glycolysis and/or maximal lactate accumulation rate are also listed. Abbr. = Abbreviation; ATP = Adenosine triphosphate; MLSS = maximal lactate steady-state (DOCX 43 KB) [file 421_2025_6022_MOESM2_ESM.docx]

**Appendix 2 Terminology and descriptions among peer-reviewed articles in English language**

| **Article** | **Terminology** | **Abbr.** | **Description (Quotations)** |
| --- | --- | --- | --- |
| Mader & Heck (1986)  (Supplement) | – maximum rate of glycolysis  – maximal lactate formation rate | dLa/dt max  $\frac{\text{dLa}}{\text{dt}}$max | […] expressed as rate of lactic acid formation.  […] between 1.3 and 0.1 mmol/s·l. |
| Grassi et al. (1995)  (Original article) | – maximal rate of lactate accumulation in blood | Δ[L̇a_b_]_max_ | […] an indirect index of the maximal glycolytic flux […].  […] could represent a valuable index for comparing, in vivo and noninvasively, the maximal glycolytic flux […] in humans. |
| Mader (2003)  (Review) | – maximal rate of glycolysis | ν_la,max_  υ_la,max_  VLa_max_ | […] expressed as rate of lactic acid formation.  […] function of the concentration of glycolytic enzymes.  […] is assumed to be about 1.0 mmol·s^-1^·kg^-1^ […] during maximal activation of glycolysis […]. |
| Heck et al. (2003)  (Review) | – maximal lactate formation / production rate  – maximal glycolytic rate  – lactic power | dLa/dt_max_ | […] an indicator for lactic power [that] cannot be measured directly with simple methods.  […] show a reduction with increasing exercise duration. |
| Sperlich et al. (2010)  (Training intervention) | – maximum rate of lactate accumulation | Lac_max_ | […] assumed to be an indicator for anaerobic performance skills.  […] higher rates […] indicate increased anaerobic energy yielding. |
| Olbrecht (2011)  (Review) | – anaerobic or glycolytic capacity | VLa_max_ | […] calculated based on their maximal metabolic activity rate. |
| Hauser et al. (2014)  (Original article) | – maximal glycolytic rate  – maximal lactate production rate | V̇La_max_ | […] test procedures of V̇La_max_ must receive greater focus in future investigations [and] the biological variability of V̇La_max_ still remains unknown.  […] can be used for explaining the origin of MLSS and therefore the metabolic response. |
| Adam et al. (2015)  (Reliability analysis) | – maximal lactate production rate  – maximal glycolytic rate  – maximum glycolytic metabolic performance | V̇La_max_ | […] represents the highest performance of the glycolysis.  […] is not established within sports medicine performance analysis. |
| Manunzio et al. (2016)  (Training intervention) | – maximal rate of lactate accumulation | dLa/dt_max_ | […] has been neglected and appears to be an underestimated parameter in terms of the origin and interpretation of MLSS. |
| Wahl et al. (2017)  (Original article) | – maximal lactate production rate | VLa_max_ | […] the 15-second sprint test generates additional information about the anaerobic capacity of an athlete like […] VLa_max_. |
| Nitzsche et al. (2018a)  (Original article) | – maximum rate of lactate production  – maximal lactate formation rate  (ergometer vs. load) | V̇La_max_ | […] determined from the maximum lactate difference before and after exercise and the difference between exercise time and alactic time span.  […] in dependence on time used. |
| Quittmann et al. (2018)  (Original article) | – maximal lactate accumulation rate  – lactic power | V̇La_max_ | […] is discussed as another important parameter […] that affects endurance performance […].  […] allows for a wider interpretation of the physiological profile of the athlete […].  […] promotes anaerobic and somehow limits aerobic performance measures.  […] allows for new insights into applied exercise physiology. |
| Hommel et al. (2019)  (Training intervention) | – maximal lactate production rate | V̇La_max_ | […] representing the maximal performance of the anaerobic lactic system.  […] allows for an approximation of maximal anaerobic lactacid performance […]. |
| Nitzsche et al. (2018b)  (Reliability analysis) | – maximum blood lactate concentration rate  – maximum lactate concentration rate  (reliability isokinetic) | V̇La_max_ | […] increases with accelerations in movement velocity under isokinetic force loads.  […] appears to depend on the duration of an anaerobic test […].  […] dependent on coordinative, motivational, and metabolic factors. |
| Quittmann et al. (2020)  (Reliability analysis) | – maximal lactate accumulation rate  – glycolytic power  (reliability in running) | V̇La_max_ | […] as a measure of anaerobic (lactic) power.  […] is applied to (1) characterise the athletes’ anaerobic capabilities, (2) to define individual training needs and (3) to design appropriate exercises for optimising improvements in performance […].  […] already demonstrated its sensitivity to certain training regimes […].  […] is a promising parameter of anaerobic (lactic) power in running and can be used for exercise testing of endurance athletes. |
| Nitzsche et al. (2020)  (Training intervention) | – maximal / maximum glycolysis rate  – rate of lactate accumulation | νla_max_ | […] regarded as an indirect measure of glycolysis activity […].  […] describes the efficiency of the anaerobic energy metabolism […].  […] could be an important parameter of anaerobic performance in resistance training.  […] a physiological measure of anaerobic energy metabolism [that] seems to be a significant performance parameter. |
| Quittmann et al. (2021a)  (Reliability analysis) | – maximal lactate accumulation rate | V̇La_max_ | […] a measure of glycolytic ATP production […].  […] allows for additional information concerning the physiological profile. |
| Zwingmann et al. (2020)  (Original article) | – maximal rate of lactate accumulation  – maximal anaerobic capacity | VLa_max_ | […] measurements of VLa_max_ with experienced athletes are rare in international literature.  […] ranges from 0.46 to 0.54 mmol·l^-1^·s^-1^ in cyclists and from 0.76 to 0.79 mmol·l^-1^·s^-1^ in middle-distance runners.to compliment athletic anaerobic performance. |
| Wawer et al. (2020)  (Reliability analysis) | – maximum lactate production rate  – maximal (anaerobic) glycolytic capacity | LPR_max_ | […] is deemed to be the indicator for anaerobic lactic capacity and is dependent on duration of glycolysis […].  […] is defined as the most important parameter for anaerobic capacity for sprint running […].  […] used to assess the maximal flow rate of glycolysis […].  […] might be a useful diagnostic tool to compliment athletic anaerobic performance. |
| Quittmann et al. (2021b)  (Reliability analysis) | – maximal lactate accumulation rate | V̇La_max_ | […] a promising parameter to describe the power of the glycolytic metabolism […].  […] is derived from sprint exercises lasting approximately 10 to 15 s […].  […] should be determined sport-specifically […]. |
| Treff et al. (2021)  (Review) | – maximum lactate accumulation rate  – anaerobic lactic power | V̇Lamax | […] has recently been increasingly discussed again […].  […] its interpretation is based on several theoretical assumptions […]. |
| Ji et al. (2021)  (Original article) | – maximal lactate production rate  – anaerobic (glycolytic) capacity | VLa_max_ | […] allows a differentiated interpretation of the athlete’s performance […].  […] up to now no established running-specific test procedure for VLa_max_ determination exists. |
| Quittmann et al. (2022a)  (Single case study) | – maximal lactate accumulation rate  (case report paratriathlon) | V̇La_max_ | […] as a measure of glycolytic capabilities in all disciplines […].  […] as a measure of glycolytic metabolism.  […] is derived from short sprint tests […].  […] is affected by exercise modality, highest in swimming and lowest in wheelchair racing […]. |
| Wackerhage et al. (2022)  (Review) | – maximal rate of lactate formation  – maximal glycolytic rate  maximal rate of glycolysis | ν_la.max_ | […] as an important variable when analysing the metabolism of an endurance athlete […].  […] high values of ν_la.max_ (i.e. > 1.0 mmol·l^-1^·s^-1^) are typical for sprinters.  […] measuring the ν_la.max_ is challenging. |
| Teixeira et al. (2022)  (Original article) | – maximal lactate production rate | VLa_max_ | […] represents the highest glycolysis demand ad predicts anaerobic power.  […] one of the most important physiological parameters to describe the conditioning profile in swimming […]. |
| Quittmann et al. (2022b)  (Original article) | – maximal lactate accumulation rate  – maximal / maximum rate of glycolysis | ċLa_max_ | […] testing is a time-efficient procedure and does not restrict the athletes’ training schedule […].  […] might help understand how metabolic processes interact during exercise […].  […] allows for significant (yet minor) improvements in […] performance prediction […]. |
| Dunst et al. (2023a)  (Original article) | – maximal blood lactate accumulation rate | v̇La_max_ | […] as an indirect parameter of maximal glycolytic activity.  […] reflecting the maximal glycolytic energy flux. |
| Dunst et al. (2023b)  (Original article) | – maximal rate of (blood) lactate accumulation | v̇La_max_ | […] as an indirect but practical indicator of the maximal rate of glycolysis.  […] requires valid determination of the initial interval of maximal energy production without significant contribution from glycolysis. |
| Schünemann et al. (2023)  (Original article) | – maximal lactate accumulation rate  – anaerobic glycolytic performance | ^ν^_La.max_ | […] may serve as a measure for the individual rate of anaerobic glycolytic energy metabolism […].  […] displays a wide spectrum of individual differences in rowers.  […] contributes to an individual energetic performance profile in rowing. |
| Yang et al. (2023)  (Original article) | – maximal lactate accumulation rate  – maximal rate of lactate accumulation  – maximal glycolytic rate | ^ν^_La.max_  P^ν^_La.max_ | […] allows for a more detailed input of individual characteristics of energy metabolism […].  […] oxidative metabolism should be considered when aiming to analyse a pure glycolytic rate.  […] athletes in sprint sports can maximally produce […] > 1.0 mmol·l^-1^·s^-1^. |
| Mavroudi et al. (2023)  (Original article) | – maximal lactate accumulation rate | VLa_max_ | […] is a promising index of anaerobic lactic power that can be rather easily determined in sprints lasting about 10 to 30 s.  […] can be used to estimate anaerobic power, determine individual training needs, and design appropriate exercises to improve performance […]. |
| Harnish & Miller (2023)  (Original article) | – glycolytic capacity | VLa_Max_ | […] the ability to perform repeated sprint efforts lasting between 15 - 30-sec, as well as the anaerobic energy contribution and buffering ability can play an important role in the outcome of a competition. |
| Held et al. (2023)  (Reliability analysis) | – maximal lactate accumulation rate  – anaerobic lactic power | VLamax | […] has gained scientific attention within sprint testing […].  […] has been linked to lactic anaerobic performance.  […] might be a promising approach for assessing anaerobic parameters in rowing.  […] a robust tool for diagnosing adaptations in glycolytic anaerobic performance. |
| Harnish et al. (2023)  (Reliability analysis) | – maximum lactate accumulation rate  – glycolytic rate  – glycolytic capacity | VLamax | […] has been proposed as an estimate of maximum anaerobic energy contribution […].  […] has been used to improve training and performance programming […].  […] remains our best estimate of glycolytic rate.  […] may not be precise enough to judge specific training responses or make detailed training recommendations. |
| Haase et al. (2024)  (Original article) | – maximal lactate formation rate  – lactic power | υLa_max_ | […] an input variable to characterize an athlete’s anaerobic power during endurance events […].  […] results contribute to the evaluation of current methodologies in anaerobic diagnostics […].  […] pedalling frequencies of at least 130 rpm […] are necessary to ream a maximal lactate formation rate. |
| Thron et al. (2024)  (Original article) | – maximal lactate accumulation rate  **–** anaerobic power | ċLa_max_ | […] the power of substrate level phosphorylation (glycolysis) […].  […] is considered a parameter that can describe anaerobic capabilities […]. |
| Wagner et al. (2024)  (Original article) | – maximal glycolytic capacity  – anaerobic capacity | VLa_max_ | […] substantial role of anaerobic capacity, constituting approximately 20-30% of total energy expenditure during maximal efforts […]. |
| Langley et al. (2024)  (Original article) | – maximal lactate formation rate  – maximal lactate accumulation rate  – glycolytic power | V̇La_max_ | […] has gained traction in both academic literature and applied settings.  […] purposes to determine the maximal glycolytic rate of the athlete indirectly via the maximal rate of blood lactate formation.  […] offers an attractive alternative from current test procedures due to its practical and minimally invasive methodological approach.  […] the optimal sample time to determine peak blood lactate was 5 ± 2 min. |
| Poffé et al. (2024)  (Original article) | – maximal lactate formation rate | VLa_max_ | […] calculated as the ratio of lactate accumulation over the time of lactate production […]. |
| Yang et al. (2024)  (Letter to the editor) | – maximal accumulation of blood lactate | νLa_max_ | […] testing is usually conducted in isokinetic mode set to 120-130 [rpm…]. |
| Meixner et al. (2024a)  (Reliability analysis) | – rate of capillary blood lactate concentration  – peak accumulation | νLa_max_ | […] has been widely discussed among athletes and within the coaching community as a feasible indicator to calculate the glycolytic capacity […]. |
| Reinpõld et al. (2024)  (Original article) | – lactate accumulation rate  – glycolytic power | V_LAmax_  VLa_max_ | […] endurance-adapted road cyclists […] have lower V_LAmax_ values of 0.43 mmol/L/l, compared to 0.95 mmol/L/s in elite track sprinters. |
| Meixner et al. (2024b) (Original article) | – maximal rate of change in capillary blood lactate concentration  – maximal rate of capillary blood lactate accumulation | νLa_max_ | […] is a crucial element within Mader’s theoretical model designed to estimate the interplay between aerobic and anaerobic energy pathways at the cellular level.  […] potential applications for interpretation of sprint testing, redefining simulation models […] and performance-enhancing interventions […]. |
| Archacki et al (2024)  (Original article) | (– lactate concentration at peak) | (LA_PEAK_) | […] athletic groups presented almost equal small effect sizes I the glycolytic energy expenditure.  […] if the ongoing long-term adaptation exists, the sex differences in the contribution of energy systems tend to decrease. |
| Pohl et al. (2024)  (Pilot study) | – maximal glycolytic rate | νLa.max | […] an emerging parameter in performance diagnostics.  […] has recently resurrected as a surrogate of anaerobic glycolytic exercise performance.  […] gained popularity in the field of athletic testing, as it is frequently discussed […].  […] influences endurance performance because […it] results in a lower […MLSS].  […] is initiated by a maximal sprint performance during which skeletal muscle glycolysis is maximally stimulated […]. |
| Sengoku et al. (2024)  (Reliability analysis) | – maximal lactate accumulation rate  – glycolytic power | ċLa_max_  ċLamax | […] is also an index to access anaerobic capacity.  […] is an extremity and movement-specific parameter.  […] has potential applications in predicting individual pacing strategies.  […] is a good indicator of front crawl sprint performance for well-trained male swimmers. |
| Dunst et al. (2025)  (Review article) | – maximal blood lactate accumulation rate  – maximal lactate accumulation rate | νLa_max_ | […] evaluates anaerobic performance by reflecting the highest rate at which lactate accumulates in the blood during short maximal exercise […].  […] a surrogate for exercise-specific maximal glycolytic flux.  […] may ot accurately reflect true changes in maximal glycolytic flux, but rather differences in lactate kinetics.  […] may peak at significantly higher cadences (> 170 rpm) tha those typically used in testing protocols (120-130 rpm) […]. |
| Harnish et al. (2024)  (Reliability analysis) | – maximum rate of lactate accumulation | V̇Lamax  VLamax | […] has been suggested to estimate of the maximum aaerobic energy cotributio to exercise […].  […] there is no consensus on what demarcates low, moderate, or high […].  […] has been use to optimize training and performance. |
| Porter & Langley (2025) (Original article) | – maximal blood lactate accumulation rate  – maximal lactate accumulation rate | vLa_max_  vLamax  V̇Lamax | […] has gained traction as a non-invasive method to determine the glycolytic contribution of exercise.  […] the academic community has questioned its validity at determining the glycolytic metabolic contribution.  […] literature has identified a 10-s test duration is significant at eliciting the highest values […]. |
| Meixner et al. (2025a)  (Original Article) | – maximal glycolytic rate | νLa_max_ | […] is considered a surrogate marker for the maximal glycolytic rate.  […] has gained recognition as an indicator of metabolic performance […].  […] assist to understand how quickly an athlete produces glycolytic energy […].  […] offering valuable insights into […] glycolytic power and overall metabolic profile.  […] aids in tailoring training and nutritional strategies to optimize performance by balancing anaerobic and aerobic demands. |
| Fischer et al. (2025)  (Original article) | – maximal lactate accumulation rate | ċLa_max_ | […] is theoretically attributed a direct influence on power output at [MLSS].  […] a marker of glycolytic power […]. |
| Keller & Wahl (2025) (Original article) | – maximal lactate accumulation  – glycolytic power | ċLa_max_ | […] reflecting the importance of anaerobic glycolytic energy supply for short exercise times. |
| Sablain et al. (2025)  (Reliability analysis) | – maximal rate of lactate accumulation  – maximal glycolytic flux rate | VLa_max_ | […] has become popular within modern sports testing and training practice.  […] is based on two assumptions that may be contentious.  […] might still be a valuable metric for monitoring the dynamic interplay between [lactate] accumulation and elimination over time in athletes. |
| Haase et al. (2025)  (Original article) | – maximal rate of blood lactate accumulation | νLa_max_ | […] serves as an indicator of the maximal glycolytic flux specific to the exercise performed.  […] depends largely on the availability of glycolytic enzymes, particularly phosphofructokinase.  […] is used for example for metabolic profiling or to predict performance in cycling.  […] |
| Fernandez-Jarillo & Lomero-Arenas (2025)  (Review) | – maximal lactate accumulation rate | VLa_max_ | […] has received increased attention as an estimator of maximal glycolytic power.  […] could enhance physiological profiling by aiding the athlete assessment, training prescription, ad talent identification.  […] estimates maximal glycolytic power with moderate to excellent reliability […].  […] further research is needed to better understand this metric. |
| Clark & Macdermid (2025)  (Original Article) | – maximum / maximal glycolytic rate  – maximal-glycolytic rate | V̇La_max_ | […] has been referred to in the literature as the maximum lactate accumulation rate as opposed to the maximum glycolytic rate […].  […] is a useful marker of maximal glycolytic rate and power production.  […] provides minimal extra information over using the power output from the isokinetic test alone.  […] can indeed be related to performance in glycolytic demanding efforts.  […] currently lacks scientific validation […]. |
| Meixner et al. (2025b)  (Original article) | – maximal glycolytic rate  – maximum capillary blood lactate accumulation rate | νLa_max_  V̇Lamax | […] an estimate of glycolytic power.  […] is a reliable measure of anaerobic power.  […] a predictor for anerobic performance in many sports.  […] is assessed in a laboratory setting.  […] serves as an indirect assessment of sport-specific performance […] and holds value in performance diagnostics. |
| Wackerhage et al. (2025)  (Review) | – maximal rate of lactate accumulation  – peak rate of lactate accumulation | *v*Lamax  *v*Lamax_muscle_  *v*Lamax_blood_  *v*Lapeak | […] a variable for glycolytic power was proposed over 40 years ago […].  […] is the peak rate of glycolysis measured as the maximal rise of the lactate concentration […].  […] there is currently no universally accepted protocol to measure [it…].  […] values typically range between ∼0.2 to 1.0 mmol/L/s […]. |
| Langley et al. (2025)  (Review) | – rate of maximal blood lactate accumulation  – maximal glycolytic power | νLa_max_ | […] an indirect measure of glycolytic flux […].  […] has gained popularity amongst researchers and practitioners […].  […] as an indirect method to determine the maximal glycolytic rate. |
| Micke et al. (2025)  (Reliability analysis) | – maximal lactate accumulation rate | *v*Lamax | […] offers an understanding of the athlete’s maximum glycolytic power.  […] has emerged as a critical parameter, representing the maximal glycolytic flux […].  […] reflects the power of glycolytic energy provision rather than performance itself. |

Articles are listed in chronological order based on their acceptance. Abbr. = Abbreviation; ATP = Adenosine Triphosphate; MLSS = maximal lactate steady-state.
